# Selective α,δ-hydrocarboxylation of conjugated dienes utilizing CO_2_ and electrosynthesis†

**DOI:** 10.1039/d0sc03148h

**Published:** 2020-07-20

**Authors:** Ahmed M. Sheta, Mohammad A. Mashaly, Samy B. Said, Saad S. Elmorsy, Andrei V. Malkov, Benjamin R. Buckley

**Affiliations:** Department of Chemistry Loughborough University Ashby Road Loughborough Leicestershire LE11 3TU UK b.r.buckley@lboro.ac.uk; Department of Chemistry, Damietta University Damietta El-Gadeeda City, Kafr Saad Damietta Governorate 34511 Egypt; Department of Chemistry, Mansoura University 25 El Gomhouria St Dakahlia Governorate 35516 Egypt

## Abstract

To date the majority of diene carboxylation processes afford the α,δ-dicarboxylated product, the selective mono-carboxylation of dienes is a significant challenge and the major product reported under transition metal catalysis arises from carboxylation at the α-carbon. Herein we report a new electrosynthetic approach, that does not rely on a sacrificial electrode, the reported method allows unprecedented direct access to carboxylic acids derived from dienes at the δ-position. In addition, the α,δ-dicarboxylic acid or the α,δ-reduced alkene can be easily accessed by simple modification of the reaction conditions.

## Abstract 2

Selective electrosynthetic α,δ-hydrocarboxylation of 1,3-dienes is reported, utilising non-sacrificial electrodes that provide access to the previously challenging δ-carboxylated regioisomer.

## Graphical abstract

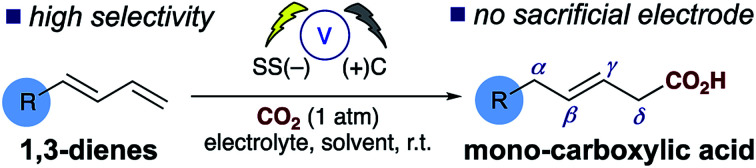

## Introduction

**Scheme 1:**
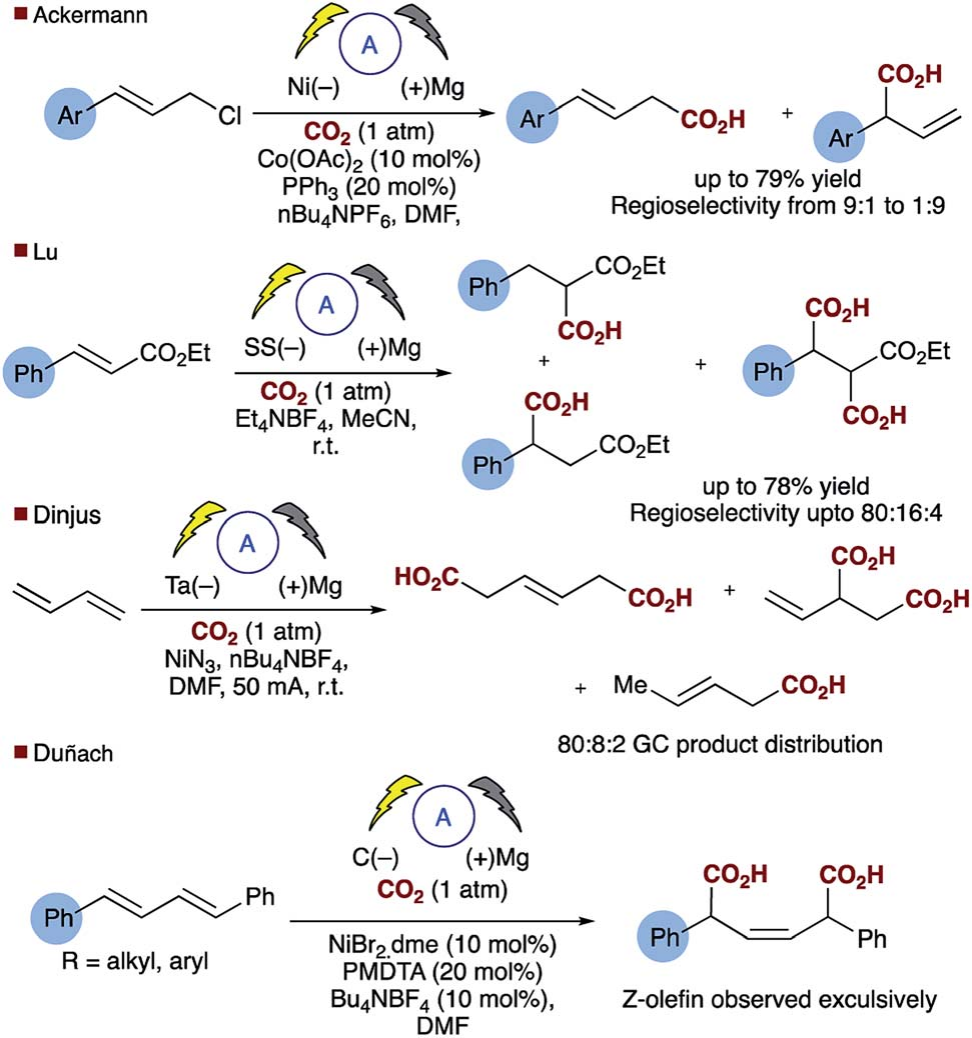
Representative attempts at selective electrocarboxylation.

**Scheme 2:**
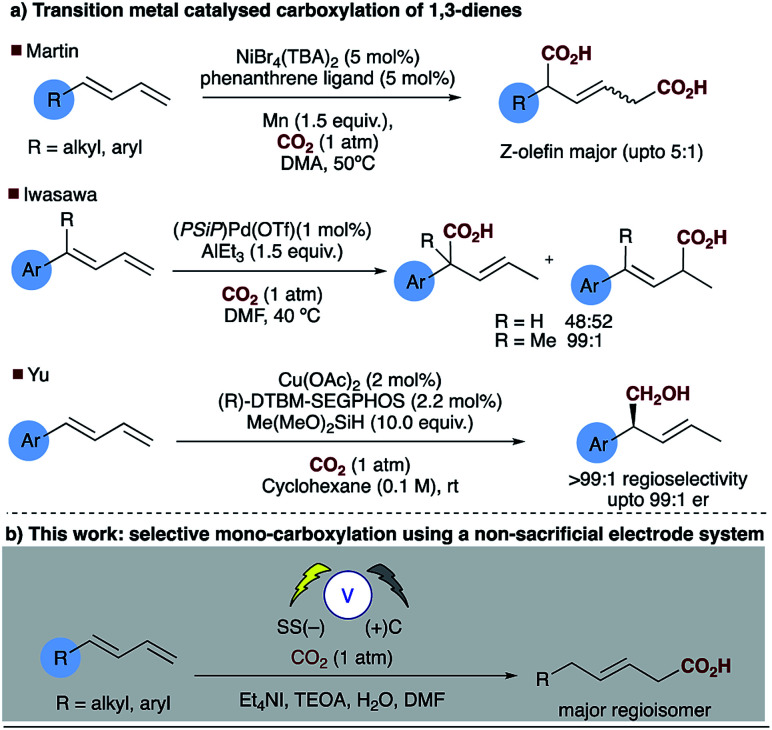
Attempts at selective carboxylation of 1,3-dienes, (a) transition metal catalysed and (b) this work.

Direct carboxylation of low value olefin feedstocks utilising carbon dioxide is regarded as a “dream reaction” owing to the desire to utilise this low value, high volume waste gas.^1–3^ However, selectivity in these, and related carboxylation reactions remains a significant challenge (see for example Scheme 1). Electrochemical approaches to carboxylation have been known for some time and recently Ackermann and others have examined the electro–reductive transition metal carboxylation of allyl chlorides with a sacrificial electrode system and up to 9 : 1 regioselectivity being observed.^4,5^ Lu has described a sacrificial magnesium anode system in which the carboxylation of α,β-unsaturated esters leads to a mixture of mono- and di-carboxylic acids.^6^ Electrochemical approaches to α,δ-dicarboxylic acids from dienes have been known for some time, early reports in the patent literature by Loveland describe the electrolytic production of acyclic carboxylic acids from olefins.^7^ However, several drawbacks to the system, such as the use of a mercury electrode, prompted further research in this area.^8^ Dinjus reported the transition metal assisted electrocarboxylation of 1,3-butadiene to afford predominantly the straight chain dicarboxylic acid, again utilising a sacrificial magnesium anode.^9^ Duñach and Perichon later described a sacrificial magnesium/nickel catalysed system with exclusive dicarboxylic acid formation and good *Z*-olefin selectivity.^10^ Aside from the electrochemical literature Mori originally reported the stoichiometric nickel dicarboxylation of 1,3-dienes and more recently Martin has described a catalytic approach to the α,δ-dicarboxylation of diene feedstocks.^11,12^ However, hydrocarboxylation of 1,3-dienes have scarcely been reported, perhaps due to the inherent selectivity issues associated with the site of carboxylation. Some selectivity has been observed for mono-carboxylation at the α-carbon by Iwasawa but this tends to depend on the substrate employed (Scheme 2a).^13^ Yu has reported a particularly impressive catalytic asymmetric approach to α-hydroxymethylation of 1,3-dienes utilising CO_2_.^14^ Only one report of selective δ-carboxylation, reported by Walther and Schonecker in the early 1990's, utilizing stoichiometric nickel complexes on a steroidal substrate is known.^15^

The electrosynthesis of carboxylates from dienes, like those from alkenes readily rely on the use of a sacrificial electrode to enable successful carboxylation and in some cases regiocontrol. As we and others have expressed previously from a practical and sustainability point of view, a non-sacrificial metal system would naturally be more desirable.^16,17^ We have recently reported the selective hydrocarboxylation of substituted aromatic alkenes utilising electrosynthesis and carbon dioxide.^16^ This novel carbon–carbon bond forming process provides unprecedented access to all carbon quaternary centres through the carboxylation of β,β-substituted olefins. Unlike the majority of previously reported transition metal catalysed approaches this chemistry selectively affords the β-carboxylated product. Herein, we present a new practical electrosynthetic approach to highly regioselective 1,4-hydrocarboxylation of dienes (Scheme 2b, Table 1, entries 1 and 2).

**Table 1:** Optimization Studies^a^

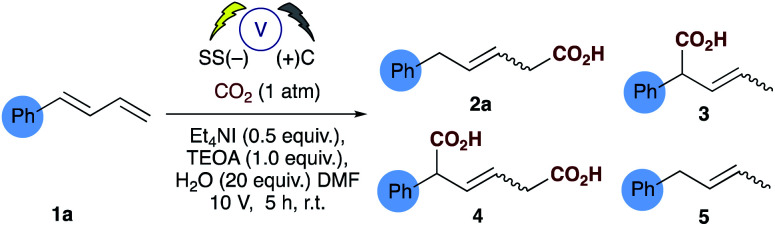						
Entry	Deviation from standard conditions	Ratio^b^				Conv. **1a**^c^ (%)
		**2a**	**3**	**4**	**5**	
1	None	9	1	0.5	0.7	99 (57)
2	No TEOA	9	1	0.5	0.7	99
3	C (−), C (+), no H_2_O	1.7	1	1	1	99
4	Ni (−), C (+), no H_2_O	0	0	0	1	80
5	Cu (−), C (+), no H_2_O	3.8	1	1	2	99
6	SS (−), C (+), no H_2_O	9	1	1	1	99
7	SS (−), SS (+), no H_2_O	3.1	1	1	0	83
8	H_2_O (5.0 equiv.)	9	1	0.82	0.7	99
9	H_2_O (10.0 equiv.)	9	1	0.74	0.7	99
10	No TEOA or H_2_O	0	0	1	0	99

a General conditions: CO_2_ (1 atm), stainless steel cathode, carbon anode, Et_4_NI (0.5 equiv.), TEOA (1.0 equiv.), DMF, single compartment cell, 10 V (60–100 mA), 5 h rt.

b Ratios determined by GC-MS analysis, all products displayed approximately a 3 : 1 *E* : *Z* ratio.

c Conversion evaluated by GC-MS analysis, numbers in parenthesis indicate isolated yield of **2a**.

## Results and discussion

Investigations commenced by utilising our previously reported conditions for alkene hydrocarboxylation, using carbon cathode and anodes (Table 1, entry 3). Disappointingly this resulted in the formation of almost a statistical mixture of the δ-monocarboxylated alkene **2a**, the α-monocarboxylated alkene **3**, the α,δ-dicarboxylated alkene **4** and the α,δ-reduced alkene **5**. Initially we screened a range of electrode pairs; Ni/C resulted in selective formation of **5** with no sign of any carboxylation by GC/MS analysis (Table 1, entry 4).^18^ Utilising Cu/C couple resulted in higher selectivity towards the δ-monocarboxylated alkene **2a** but significant amounts of the reduced diene **5** were observed. Switching to stainless steel (SS) and carbon provided a significant improvement in the selectivity towards **2a** (Table 1, entry 6) and further optimization through the addition of water (Table 1, entries 8–10) provided our standard conditions in which the side products **3**, **4** and **5** have been significantly reduced (Table 1, entry 1). Interestingly the use of stainless steel as cathode and anode eliminated the reduced product **5** but showed no improvement in selectivity towards **2a** (Table 1, entry 7). In the absence of TEOA we observed little change in the selectivity of the reaction, but more unidentifiable by-products (by GCMS) were observed (unlike the corresponding reaction carried out with alkenes^16^), however, in the absence of a suitable proton source (*e.g.* both TEOA and water) the dicarboxylic acid **4** was the sole product in up to 50% unoptimised isolated yield (Table 1, entry 10).

With the optimized conditions in hand, initial examination of the scope of the reaction was carried out with a variety of aryl and aliphatic dienes (**1a–m**, Table 2), good selectivity of the 1,4-hydrocarboxylated products were obtained and, in some cases (**2f–h**), the δ-substituted carboxylic acid was obtained exclusively. The thiophene substrate **1g** resulted in exclusive formation of **2g**. Interestingly substitution of the diene in the 4-position with a methyl group **1h** also afforded exclusive formation of the 4-substituted acid **2h**. The symmetrical diene **1f** afforded **2f** in good yield, however, for reasons which are unclear at present, its isomer **1i** did not incorporate carbon dioxide and the corresponding reduced alkene **5i** was the only detectable product by GC-MS analysis. Anthracene **1j** proved to be a good substrate and the corresponding mono-carboxylate **2j** was isolated in 65% yield. We then turned our attention to several challenging aliphatic dienes: for reasons which are unclear **1k** and **1l** did not incorporate CO_2_ under our optimised conditions.

**Table 2:** Substrate Scope^a^^,^^b^

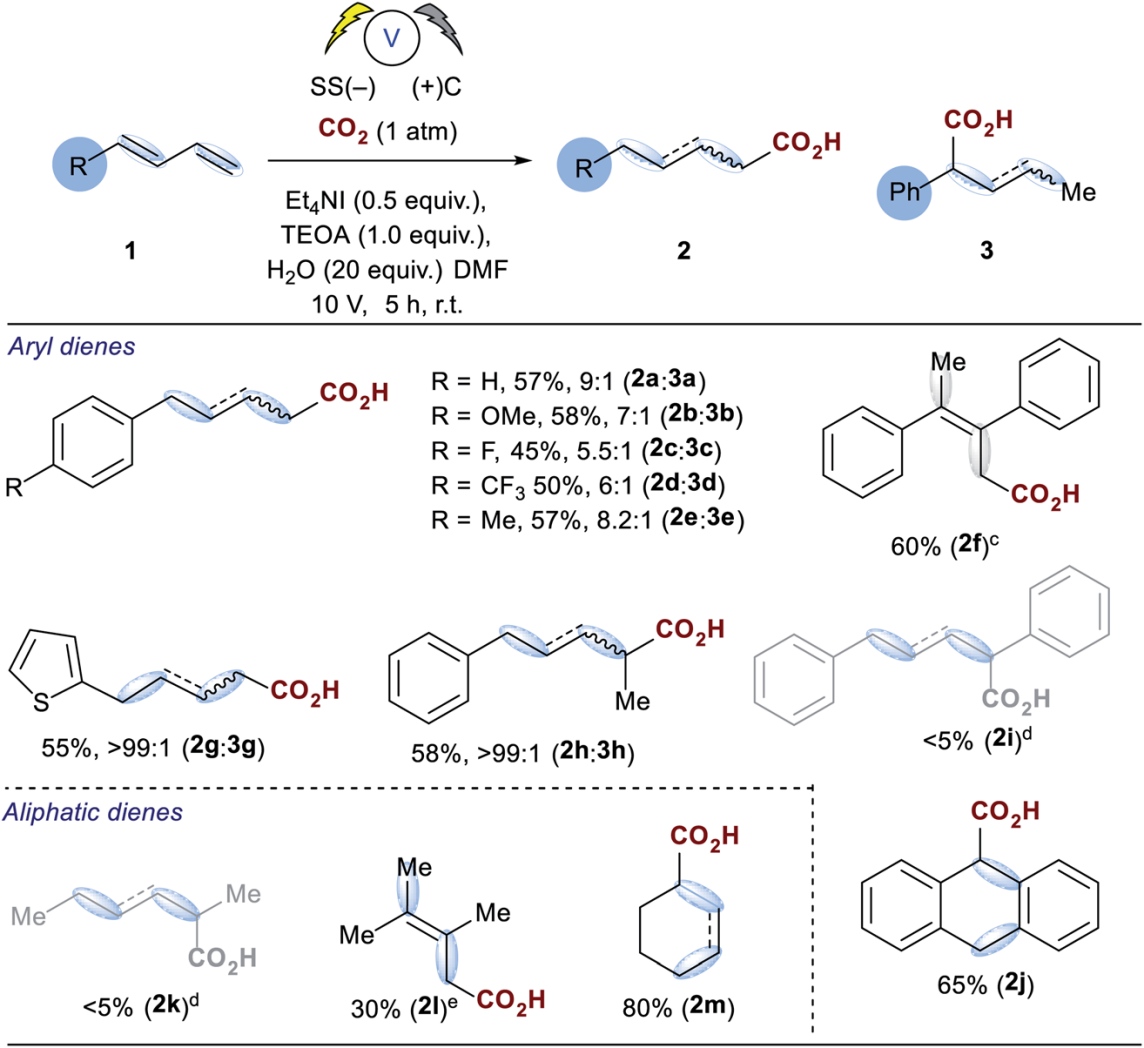

a General conditions: CO_2_ (1 atm), stainless steel cathode, carbon anode, Et_4_NI (0.5 equiv.), TEOA (1.0 equiv.), DMF, single compartment cell, 10 V (60–100 mA), 5 h rt. Products were isolated as the corresponding methyl esters and **2**/**3** ratios determined after hydrogenation of the double bond. Products **2**/**3a–e** and **2**/**3g** & **h** displayed approximately a 3 : 1 *E* : *Z* ratio.

b Ratios determined by GC-MS analysis.

c Isolated as the alkene.

d The reduced diene was the major product by GC-MS analysis.

e Conditions from Table 1, entry 6 employed.

In order to form low yields of **2l** we had to employ the non-aqueous conditions from Table 1 (entry 6). However, cyclohexadiene **1m** did react under our optimised conditions to afford a mixture of alkenes from direct and conjugate addition in excellent yield (80%).

**Fig. 1:**
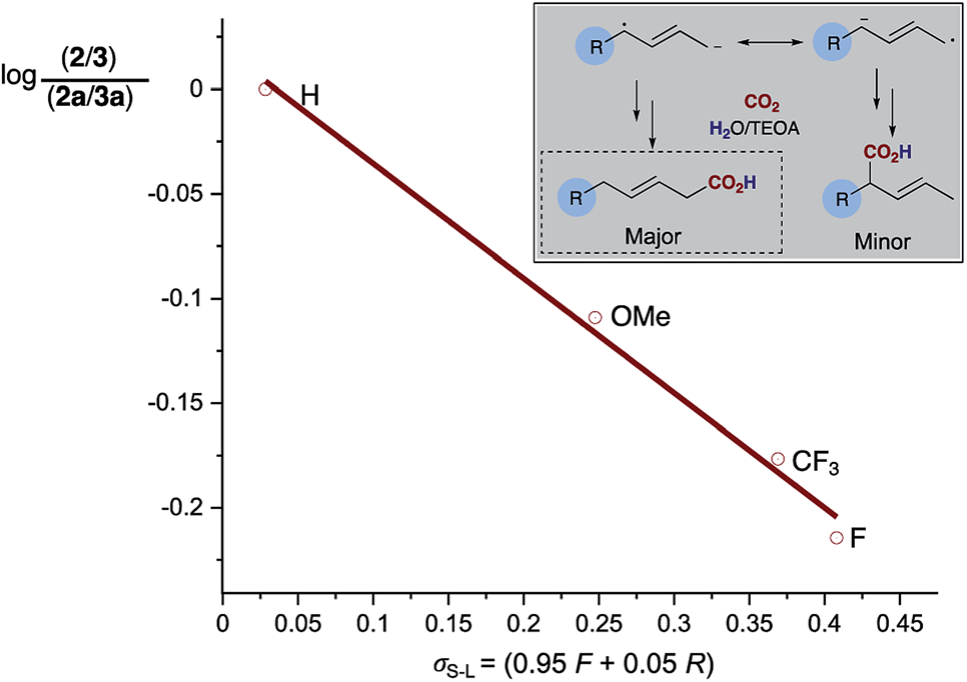
Swain–Lupton correlation of regioselectivity of carboxylation for the 4-aryl substituted 1,3-dienes (**2**/**3**).

**Scheme 3:**
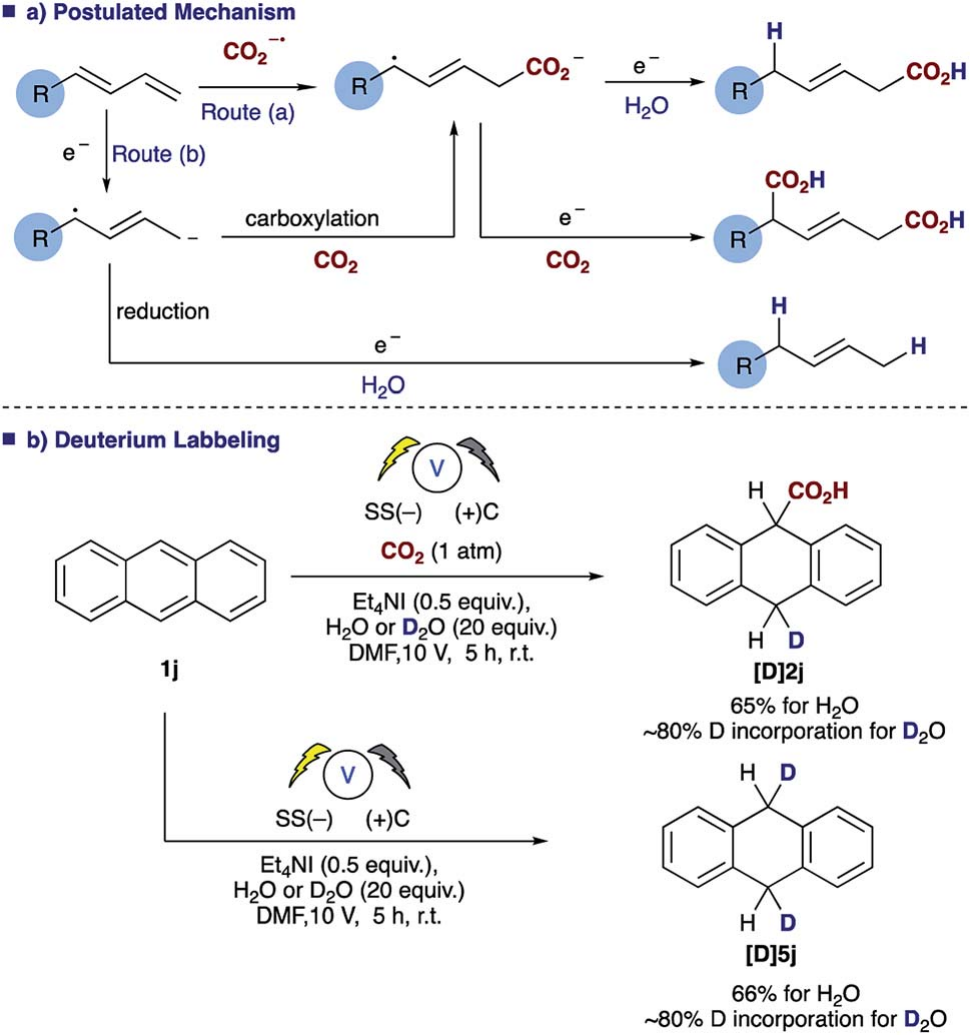
Postulated mechanism (a) and deuterium labelling studies (b).

An examination of the aryl substituent (**1a–e**) reveals that selectivity of the hydrocarboxylation (**2***vs.***3**) is in some way related to the electron density of the aryl ring. Electron withdrawing groups (4-F, CF_3_) resulted in a slightly poorer ratio when compared to more electron rich aryl substituents (4-H, Me, OMe). Initial Hammett correlation using *σ*, or the modified *σ*^+^ or *σ*^−^ values revealed no obvious correlation. However, when we plotted the regioselectivity of (**2a–d**/**3a–d**) over the modified Swain–Lupton parameters (*σ*_S–L_)^19^ a correlation (*r*^2^ = 0.992) was observed (Fig. 1).^20^ Addition of the intermediate radical anion to CO_2_ is accompanied by decrease in negative charge. Electronic effects of aryl substituents should influence benzylic radical to a lesser extent compared to benzylic anion. Therefore, a modest slope of the line (*ρ* = −0.55) with *F* (field-inductive constant) dominant over *R* (resonance constant) appears to be in line with the preferential formation of linear isomer **2**. The 4-F substituted 1,3-diene **1c** produces **2**/**3** with lower regioselectivity *vs.* 4-H, OMe and CF_3_ due to its increased ability to inductively destabilise the formation of positive character at the benzylic position. In addition, this provides further evidence that in these particular cases reduction of the diene occurs in preference to that of CO_2_, thus route b shown in Scheme 3a likely dominates.

**Fig. 2:**
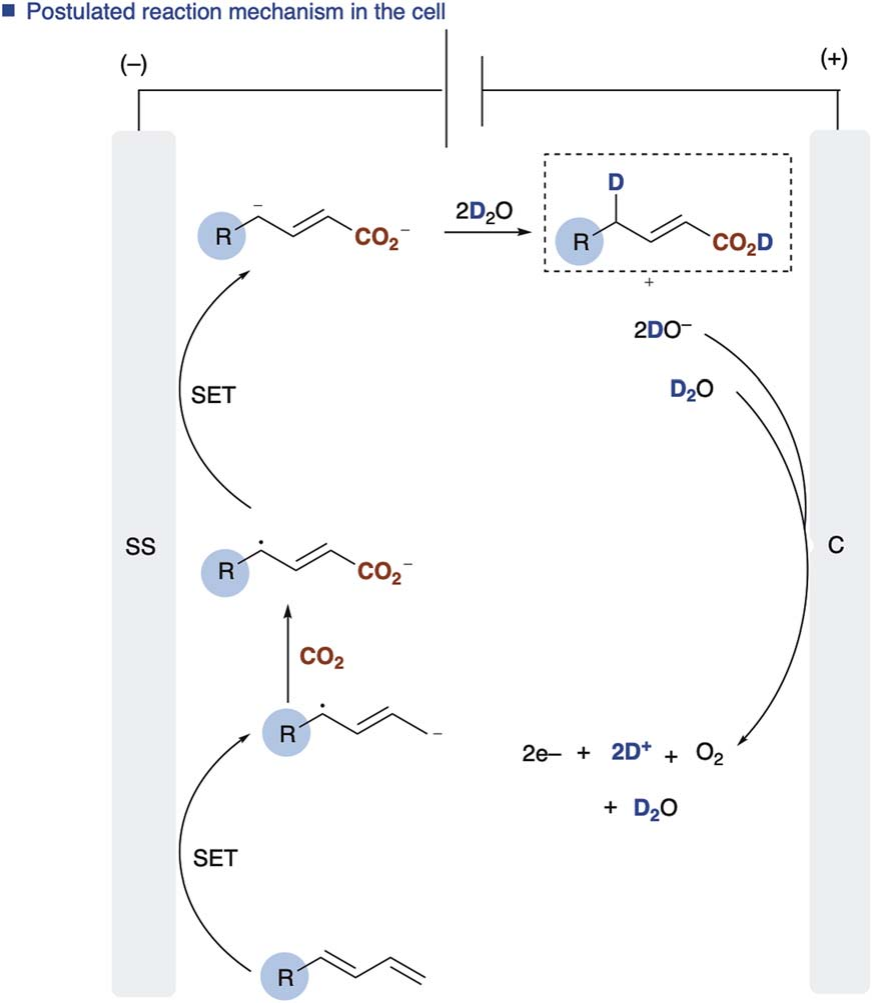
Overall processes at both the anode and cathode.

The role of water in the reaction was confirmed through deuterium labelling studies of anthracene **1j** employing D_2_O which resulted in formation of **[D]2j** or in the absence of CO_2_**[D]5j** both with ∼80% D incorporation (Scheme 3b). This leads us to propose the mechanism highlighted [Scheme 3a, route (b)] in which electron transfer to the diene proceeds to form the adsorbed radical anion of the diene, subsequent carboxylation and further electron transfer and protonation from water affords the final α,δ-monocarboxylated product. The reaction at the counter electrode has been eloquently described by Chang and co-workers in their study of deuteration reactions employing electrosynthesis and D_2_O.^21^ In the absence of water the dicarboxylated product dominates and in the absence of CO_2_ the α,δ-reduction of the diene occurs (Fig. 2).

**Scheme 4:**
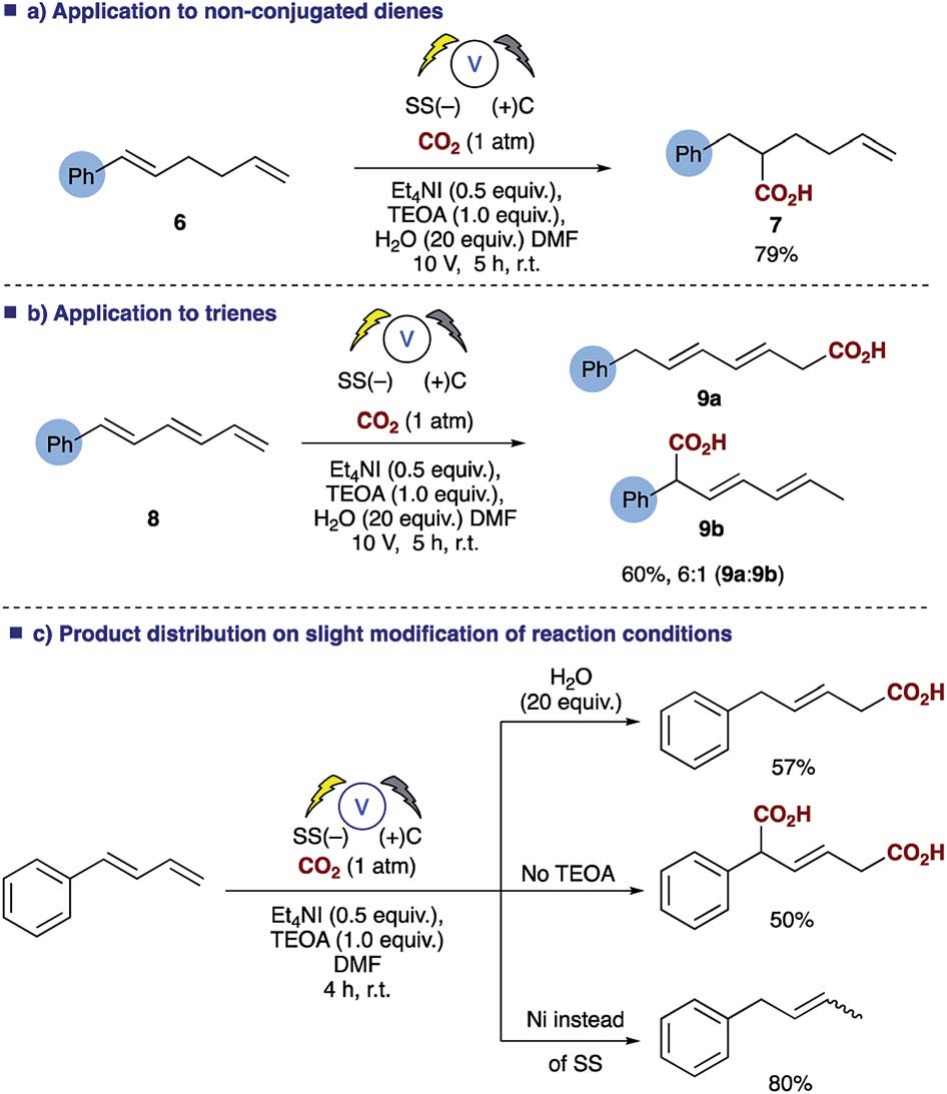
Summary of the versatility of the electrosynthetic conditions to selectively afford 3 different products.

Probing the substrates a little further revealed that selective carboxylation of non-conjugated alkenes, such as **6**, is possible under the reaction conditions affording the α,β-hydrocarboxylated product **7** (Scheme 4a), thus demonstrating that conjugation of the diene is essential for successful carboxylation to occur, in a similar fashion to the reported styrene hydrocarboxylation.^16^ The methodology could also be extended to trienes such as **8** with the α,ϕ-hydrocarboxylated product **9a** predominating when using the conditions employed for dienes in Table 2 (Scheme 4b). In addition, depending on the reaction conditions employed one can choose the product distribution required (Scheme 4c); addition of H_2_O to reaction provides the α,δ-monocarboxylated product; removal of any proton source affords the solely the α,δ-dicarboxylated product and switching the electrode system results in α,δ-reduction of the diene.

## Conclusions

In summary, a highly regioselective hydrocarboxylation process that enables the direct formation of carboxylic acids from dienes giving access to α,δ-hydrocarboxylation products has been reported. A wide variety of substrates have been tolerated under these electrosynthetic conditions; Thus, this approach is complimentary to the current literature in which α-addition and dicraboxylation dominates. Preliminary mechanistic studies suggest that field inductive effects are dominant over resonance effects in the transition state. Thus lower regioselectivties of **2***vs.***3** are observed when the 4-substituent is inductively withdrawing. The current process goes beyond these state-of-the-art systems enabling the selective mono-carboxylation of 1,3-dienes, non-conjugated dienes and trienes.

## Conflicts of interest

There are no conflicts to declare.

## Supplementary Material

SC-011-D0SC03148H-s001
