# Supplementary material for: Selective α,δ-hydrocarboxylation of conjugated dienes utilizing CO2 and electrosynthesis
Source: Chem Sci. 2020 Jul 20;11(34):9109–14. doi: 10.1039/d0sc03148h (PMC8163448; doi:10.1039/d0sc03148h)
Supplement: SC-011-D0SC03148H-s001 [file SC-011-D0SC03148H-s001.pdf]

Electronic Supplementary Material (ESI) for Chemical Science.
This journal is © The Royal Society of Chemistry 2020

Supporting Information

Selective α,δ-hydrocarboxylation of conjugated dienes utilizing CO_2 and electrosynthesis

1.0 General Experimental

Reagents: Commercially available materials (electrolytes, reducing agents, dienes) were used without further purification. Anthracene (**1j**), *trans,trans*-1,4-diphenyl-1,3-butadiene (**1i**), hexa-2,4-diene (**1k**), 2,3-dimethylbutadiene (**1l**) and cyclohexadiene (**1m**) were purchased from commercial sources and used as received. Anhydrous N,N-dimethylformamide and THF were purchased from Sigma-Aldrich and dried over 3Å molecular sieves prior to use. Tetraethylammonium iodide was also purchased from Sigma-Aldrich. Deuterated solvents were purchased from Fluorochem UK Ltd.

Analytical Methods: All infrared spectra were obtained using a Perkin-Elmer Spectrum 65 FT-IR spectrophotometer; thin film spectra were acquired using sodium chloride plates. All ^{1}H and ^{13}C NMR spectra were measured at 400 and 100 MHz using a Bruker Avance 400 MHz spectrometer, a Jeol ECS 400 MHz spectrometer or at 500 and 125 MHz on a Jeol ECZ 500 MHz spectrometer. The solvent used for NMR spectroscopy was $CDCl_3$ (unless stated otherwise) using TMS (tetramethylsilane) as the internal reference. Chemical shifts are given in parts per million (ppm) and J values are given in Hertz (Hz).

Analysis by GCMS utilised a Shimadzu QP2020, GC-2010 Plus, using a 15 m x 0.25 mm DB-5 column and an electron impact low resolution mass spectrometer, acids were converted to their corresponding methyl esters using TMSdiazomethane prior to sampling. Melting points were recorded using a Stuart Scientific melting point apparatus SMP3 and are uncorrected. All chromatographic manipulations used silica gel as the adsorbent. Reactions were monitored by GCMS or using thin layer chromatography (TLC) on aluminium backed plates with Merck Kiesel 60 F254 silica gel. TLC visualised by UV radiation at a wavelength of 254 nm. Purification by column chromatography used Apollo Scientific 60 40-63μm silica gel.

Electrode electrochemical reactions were carried out using a 10mL reaction vial using a carbon anode and a stainless steel cathode supplied by IKA and the current was supplied from an IKA ElectraSyn 2.0

2.0 Procedure for preparing 1,3-dienes via Wittig reaction

General Procedure I: A suspension of allyltriphenylphosphonium bromide (10 mmol) in 50 mL of THF under inert atmosphere was cooled at 0 °C with an ice bath. Then, n-BuLi (10 mmol, 4 mL, 2.5M in hexanes) was added dropwise. The reaction was stirred for 1hr at 0 °C and then the corresponding aldehyde (8 mmol dissolved in 5 mL of THF) was added dropwise. The reaction was stirred for 12 hours at room temperature. After this time, it was quenched with 10 mL of methanol and 10 g silica gel was added. The solvent was removed under reduced pressure and the crude product was purified by column chromatography using hexane on silica gel.

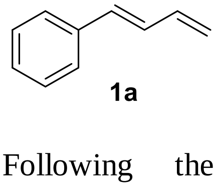

1a

(*E*)-buta-1,3-dien-1-ylbenzene (1a). Following the general procedure I, but using methyltriphenylphosphonium bromide (3.58 g, 10 mmol) and *trans*-cinnamaldehyde (1.0mL, 8 mmol) to give **1a** (0.9 g, 86.5% yield). Colorless liquid.

^{1}H NMR (400 MHZ, $CDCl_3$) δ 7.33-7.31 (m, 2H), 7.25-7.21 (m, 2H), 7.16-7.12 (m, 1H), 6.71 (ddt, *J* = 15.5, 10.5, 0.8 Hz, 1H), 6.50 – 6.38 (m, 2H), 5.25 (dd, *J* = 17.2, 0.6 Hz, 1H), 5.10 (dd, *J* = 10.4, 0.6).

^{13}C NMR (101 MHz, $CDCl_3$) δ 137.3, 137.2, 132.9, 129.7, 128.7, 127.7, 126.5, 117.7 ppm.

Spectral data is in agreement with the literature.[1]

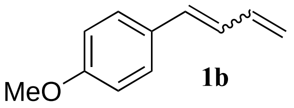

1b

(*E*,*Z*)-1-(buta-1,3-dien-1-yl)-4-methoxybenzene (1b). Following the general procedure I using 4-methoxybenzaldehyde (0.97 mL, 8 mmol), affording **1b** (1.1 g, 86% yield) as a mixture of isomers (*E:Z* = 1.6:1). Yellow liquid

^{1}H NMR (400 MHZ, $CDCl_3$) δ 7.23 (d, *J* = 8.7 Hz, 2H, *E* isomer), 7.18 (d, *J* = 8.5 Hz, 2H, *Z* isomer), 6.83 (d, *J* = 8.8 Hz, 2H, *Z* isomer), 6.78 (d, *J* = 8.7 Hz, 2H, *E* isomer), 6.57 (d, *J* = 15.7 Hz, 1H, *E* isomer), 6.38 (dt, J=16.8, 10.3 Hz, 1H, *E* isomer), 6.09 (d, *J* = 11.5 Hz, 1H, *Z* isomer), 5.27 (ddt, *J* = 16.9, 1.8, 0.8 Hz, 1H, *Z* isomer), 5.18 (dd, *J* = 16.0, 0.8 Hz, 1H, *E* isomer), 5.10 (dddd, *J* = 10.2, 2.1, 1.4, 0.9 Hz, 1H, *Z* isomer), 5.00 (d, *J* = 9.3 Hz, 1H, *E* isomer), 3.70 (s, 3H, *E* isomer), 3.69 (s, 3H, *Z* isomer) ppm.

^{13}C NMR (101 MHz, $CDCl_3$) δ 159.4 (*E* isomer), 158.8 (*Z* isomer), 137.5 (*E* isomer), 133.5 (*Z* isomer), 132.5 (*E* isomer), 130.4 (*Z* isomer), 130.3 (*Z* isomer), 130.2 (*Z* isomer), 130.1 (*E* isomer), 129.5 (*Z* isomer), 119.1 (*Z* isomer), 127.9 (*E* isomer), 127.8 (*E* isomer), 116.6 (*E* isomer), 114.2 (*E* isomer), 113.8 (*Z* isomer), 55.3 (*Z* isomer), 55.3 (*E* isomer) ppm.

Spectral data is in agreement with the literature.[2]

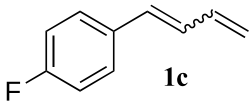

1c

(*E*,*Z*)-1-(buta-1,3-dien-1-yl)-4-fluorobenzene (1c). Following the general procedure I using 4-fluorobenzaldehyde (0.85 mL, 8 mmol), affording **1c** (0.9 g, 76% yield) as a mixture of isomers (*E:Z* = 1:1.5). Yellow liquid.

^{1}H NMR (400 MHZ, $CDCl_3$) δ 7.38-7.34 (m, 2H, *E* isomer), 7.28 (dddd, *J* = 8.6, 5.4, 2.7, 1.6 Hz, 2H, *Z* isomer), 7.06-6.98 (m, 2H), 6.83 (dddd, *J* = 16.8, 11.2, 10.1, 1.1 Hz, 1H, *Z* isomer), 6.73-6.67 (m, 1H, *E* isomer), 6.52 (d, *J* = 15.6 Hz, 1H, *E* isomer), 6.48 (dt, *J* = 16.9, 10.3 Hz, 1H, *E* isomer), 6.40 (d, *J* = 11.5 Hz, 1H, *Z* isomer), 6.25 (t, *J* = 11.3 Hz, 1H, *Z* isomer), 5.35 (dd, *J* = 16.9, 1.8, 1H, *Z* isomer), 5.25-5.23 (m, 1H), 5.17 (dd, *J* = 9.6, 1.2 Hz, 1H, *E* isomer) ppm.

^{13}C NMR (101 MHz, $CDCl_3$) δ 162.2 (d, *J* = 240.0 Hz, *E* isomer), 159.7 (d, *J* = 194.4 Hz, *Z* isomer), 135.9 (*E* isomer), 132.3 (d, *J* = 3.5 Hz, *E* isomer), 132.2 (d, *J* = 3.5 Hz, *Z* isomer), 131.7 (*Z* isomer), 130.5 (*E* isomer), 129.5 (d, *J* = 1.3 Hz, *Z* isomer), 129.4 (d, *J* = 7.9 Hz, *Z* isomer), 128.3 (d, *J* = 7.6 Hz, *E* isomer), 128.1 (*Z* isomer), 126.8 (d, *J* = 34.4 Hz, *E* isomer), 118.8 (*Z* isomer), 116.6 (*E* isomer), 114.4 (d, *J* = 37.6 Hz, *E* isomer), 114.0 (d, *J* = 32.5 Hz, *Z* isomer) ppm.

^{19}F NMR (376 MHz, $CDCl_3$) δ -114.0 (*E* isomer), -114.6 (*Z* isomer) ppm.

Spectral data is in agreement with the literature.[2]

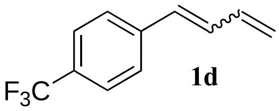

1d

(*E*,*Z*)-1-(buta-1,3-dien-1-yl)-4-(trifluoromethyl)benzene (1d). Following the general procedure I using 4-trifluoromethylbenzaldehyde (1.09 mL, 8 mmol), affording **1d** (0.95 g, 60% yield) as a mixture of isomers (*E*:*Z* = 1:1). Yellow liquid.

^{1}H NMR (400 MHZ, $CDCl_3$) δ 7.52 (d, *J* = 8.2 Hz, 2H, *Z* isomer), 7.49 (d, *J* = 8.8 Hz, 2H, *E* isomer), 7.41(d, *J* = 8.5 Hz, 2H, *E* isomer), 7.34 (d, *J* = 8.5 Hz, 2H, *Z* isomer), 6.82-6.69 (m, 1H), 6.50 (d, *J* = 15.7 Hz, 1H, *E* isomer), 6.47 (dt, *J* = 16.9, 9.9 Hz, 1H, *E* isomer), 6.42 (d, *J* = 11.6 Hz, 1H, *Z* isomer), 6.27 (t, *J* = 11.4 Hz, 1H, *Z* isomer), 5.39 (ddt, *J* = 16.8, 1.7, 0.8 Hz, 1H, *Z* isomer), 5.33 (d, *J* = 16.9 Hz, 1H, *E* isomer), 5.24 (dtd, *J* = 10.2, 1.6, 0.9 Hz, 1H, *Z* isomer), 5.18 (m, 1H, *E* isomer) ppm.

^{13}C NMR (101 MHz, $CDCl_3$) δ 140.9 (*Z* isomer), 140.6 (*E* isomer), 136.7 (*E* isomer), 132.6 (*Z* isomer), 132.5 (*Z* isomer), 132.0 (*E* isomer), 131.2 (*E* isomer), 129.5 (*E* isomer), 129.2 (*Z* isomer), 128.8 (*E* isomer), 126.5 (*Z* isomer), 125.6 (q, *J* = 3.9 Hz, *E* isomer), 125.2 (q, *J* = 3.9 Hz, *Z* isomer), 122.9 (*E* isomer), 121.2 (*Z* isomer), 119.4 (*Z* isomer) ppm.

^{19}F NMR (376 MHz, $CDCl_3$) δ -62.3 ppm.

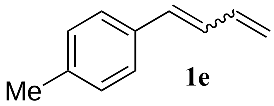

1e

(*E*,*Z*)-1-(buta-1,3-dien-1-yl)-4-methylbenzene (1e). Following the general procedure I using 4-methylbenzaldehyde (0.92 mL, 8 mmol), affording **1e** (0.95 g, 82.4% yield) as a mixture of isomers (*E*:*Z* = 1.6:1). Colorless liquid

^{1}H NMR (400 MHZ, $CDCl_3$) δ 7.20 (d, *J* = 8 Hz, 2H, *E* isomer), 7.13 (d, *J* = 8.4 Hz, 2H, *Z* isomer), 7.04 (d, *J* = 8 Hz, 2H, *E* isomer), 7.02 (d, *J* = 8 Hz, 2H, *Z* isomer), 6.80 (dd, *J* = 15.7, 10.6 Hz, 1H, *E* isomer), 6.65 (dd, *J* = 15.6, 10.4 Hz, 1H, *Z* isomer), 6.44 (d, *J* = 15.9 Hz, 1H, *E* isomer), 6.44 (d, *J* = 15.6 Hz, 1H, *Z* isomer), 6.34 (m, 2H), 5.27 (ddt, *J* = 16.9, 1.7, 0.8 Hz, 1H, *Z* isomer), 5.23 (dd, *J* = 16.1, 0.8 Hz, 1H, *E* isomer), 5.11 (dddd, *J* = 10.5, 2.2, 1.4, 0.9 Hz, 1H, *Z* isomer), 5.04 (d, *J* = 9.5 Hz, 1H, *E* isomer), 2.25 (s, 3H, *E* isomer), 2.23 (s, 3H, *Z* isomer).

^{13}C NMR (101 MHz, $CDCl_3$) δ 137.6 (*E* isomer), 137.4 (*Z* isomer), 136.9 (*E* isomer), 134.6 (*Z* isomer), 134.5 (*E* isomer), 133.5 (*Z* isomer), 132.9 (*Z* isomer), 130.5 (*Z* isomer), 130.3 (*E* isomer), 129.4 (*Z* isomer), 129.3 (*Z* isomer), 129.1 (*E* isomer), 128.8 (*E* isomer), 126.5 (*E* isomer), 119.3 (*E* isomer), 117.1 (*Z* isomer), 21.3 (*Z* isomer), 21.3 (*E* isomer) ppm.

Spectral data is in agreement with the literature.[1]

Spectral data is in agreement with the literature.[3]

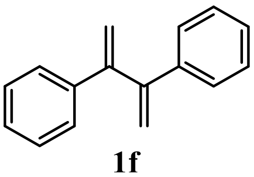

1f

2,3-Diphenyl-1,3-butadiene (1f). Following the general procedure I but using methyltriphenylphosphonium bromide (7.16 g, 20 mmol) and benzil (1.68 g, 8 mmol), affording **1f** (1.2 g, 73% yield). Colorless solid.

^{1}H NMR (400 MHZ, $CDCl_3$) δ 7.40 - 7.38 (m, 4H), 7.38 - 7.21 (m, 6H), 5.54 (d, *J* = 1.6 Hz, 2H), 5.31 (d, *J* = 1.6 Hz, 2H).

^{13}C NMR (101 MHz, $CDCl_3$) δ 149.9, 140.2, 128.2, 127.5, 116.4.

Spectral data is in agreement with the literature.[6]

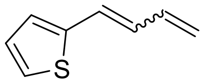

1g

(*E*,Z)-2-(buta-1,3-dien-1-yl)thiophene (1g). Following the general procedure I using 2-thiophenecarboxaldehyde (0.75 mL, 8 mmol), affording **1g** (0.9 g, 87% yield) as a mixture of isomers (*E:Z* = 1:1.5). Yellow liquid.

^{1}H NMR (500 MHz, $CDCl_3$) δ 7.17 (dd, *J* = 5.5, 1.5 Hz, 1H, *E* isomer), 7.09 (dd, *J* = 5.5, 1.5 Hz, 1H, *Z* isomer), 7.01 (m, 1H), 6.93 – 6.85 (m, 1H), 6.60-6.48 (m, 1H), 6.40 – 6.30 (m, 1H), 6.04 (td, *J* = 11.1, 0.9 Hz, 1H), 5.31 (ddd, *J* = 16.9, 1.8, 0.9 Hz, 1H, *Z* isomer), 5.21 (d, *J* = 16.8 Hz, 1H, *E* isomer), 5.05 (m, 1H) ppm.

^{13}C NMR (126 MHz, $CDCl_3$) δ 142.6, 140.3, 136.7, 133.3, 129.4, 128.5, 128.4, 127.6, 127.0, 126.8, 126.1, 125.8, 124.6, 122.6, 120.2, 117.6 ppm.

Spectral data is in agreement with the literature.[5]

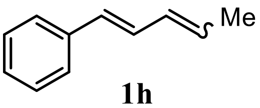

1h

(*E*,*Z*)-Penta-1,3-dien-1-ylbenzene (1h). Following the general procedure I but using ethyltriphenylphosphonium bromide (3.71 g, 10 mmol) and cinnamaldehyde (1 mL, 8 mmol), affording **1h** (1.2 g, 83% yield) as a mixture of isomers (*E:Z* = 1:1.6). Colorless liquid.

^{1}H NMR (400 MHZ, $CDCl_3$) δ 7.37 (d, *J* = 8 Hz, 2H, *Z* isomer), 7.32 (d, *J* = 7.2 Hz, 2H, *E* isomer), 7.28 – 7.22 (m, 2H), 7.18 – 7.14 (m, 1H), 7.07 (ddd, *J* = 15.6, 11.2, 4.4 Hz, 1H, *Z* isomer), 6.70 (dd, *J* = 15.6, 12 Hz, 1H, *E* isomer), 6.48 (d, *J* = 15.6 Hz, 1H, *Z* isomer), 6.37 (d, *J* = 16 Hz, 1H, *E* isomer), 6.20 – 6.13 (m, 1H), 5.77 (dq, *J* = 13.9, 6.8 Hz, 1H, *E* isomer), 5.68 (dq, *J* = 10.7, 7.2 Hz, 1H, *Z* isomer), 1.82 (dd, *J* = 7.2, 1.6 Hz, 3H, *Z* isomer), 1.77 (dd, *J* = 6.8, 1.0 Hz, 3H, *E* isomer) ppm.

^{13}C NMR (101 MHz, $CDCl_3$) δ 137.9, 132.2, 130.4, 130.1, 130.0, 129.5, 128.8, 128.7, 127.5, 127.2, 126.6, 126.4, 124.3, 18.6, 13.8.

Spectral data is in agreement with the literature.[4]

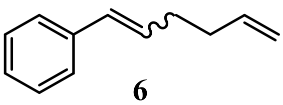

6

(*E*,*Z*)-1,5-Hexadien-1-ylbenzene (6). Following the general procedure I but using benzaldehyde (0.82mL, 8 mmol) and 4-Pentenyltriphenylphosphonium bromide, affording **6** (1.0 g, 80% yield) as a mixture of isomers (*E:Z* = 1:1). Colorless liquid

^{1}H NMR (500 MHZ, $CDCl_3$) δ 7.37-7.20 (5H,m), 6.44 (1H,m), 5.85 (1H, m), 5.69 (1H, m), 5.05 (2H, m), 2.44 (2H,m), 2.23 (2H,m).

^{13}C NMR (126 MHz, $CDCl_3$) δ 138.1, 137.8, 137.1, 132.1, 130.3, 130.2, 129.3, 128.8, 128.5, 128.3, 128.2, 126.9, 126.6, 126.0, 115.0, 34.0, 33.6, 32.5, 28.0 ppm.

Spectral data is in agreement with the literature.[19]

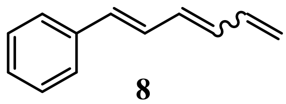

8

(1*E*,3(*E,Z*))-(hexa-1,3,5-trien-1-yl)benzene (8). Following the general procedure I using *trans*-cinnamaldehyde (1.0mL, 8 mmol), affording **8** (1.0 g, 80% yield) as a mixture of isomers (*E*:*Z* = 1:1). White solid

^{1}H NMR (500 MHZ, $CDCl_3$) δ 7.32 (d, *J* = 7.6 Hz, 2H, *E* isomer), 7.28 (d, *J* = 7.2 Hz, 2H, *Z* isomer), 7.22 (t, *J* = 7.0 Hz, 2H, *E* isomer), 7.19 (t, *J* = 7.2 Hz, 2H, *Z* isomer), 7.15 – 7.09 (m, 1H), 6.84 (dd, *J* = 15.6, 10.2 Hz, 1H, *E* isomer), 6.70 (dd, *J* = 15.4, 10.2 Hz, 1H, *Z* isomer), 6.48 (d, *J* = 15.9 Hz, 1H, *E* isomer), 6.44 (d, *J* = 15.6 Hz, 1H, *Z* isomer), 6.36 – 6.21 (m, 2H), 6.07 (t, *J* = 11.0 Hz, 1H, *E* isomer), 5.98 (t, *J* = 11.0 Hz, 1H, *Z* isomer), 5.21 (dd, *J* = 16.9, 1.2 Hz, 1H, *Z* isomer), 5.17 (dd, *J* = 16.1, 0.8 Hz, 1H, *E* isomer), 5.11 (dddd, *J* = 10.5, 2.2, 1.4, 0.9 Hz, 1H, *Z* isomer), 5.02 (d, *J* = 9.5 Hz, 1H, *E* isomer).

^{13}C NMR (126 MHz, $CDCl_3$) δ 137.4 (*E* isomer), 137.4 (*Z* isomer), 137.2 (*E* isomer), 133.9 (*E* isomer), 133.7 (*E* isomer), 133.6 (*E* isomer), 133.1 (*Z* isomer), 132.3 (*Z* isomer), 130.5 (*Z* isomer), 130.3 (*Z* isomer), 128.9 (*E* isomer), 128.8 (*E* isomer), 128.8 (*Z* isomer), 127.9 (*E* isomer), 127.7 (*Z* isomer), 126.7 (*E* isomer), 126.5 (*Z* isomer), 124.1 (*Z* isomer), 118.5 (*E* isomer), 117.6 (*Z* isomer) ppm.

Spectral data is in agreement with the literature.[7]

3.0 Electrocarboxylation of dienes using a non-sacrificial electrochemical cell (Initial Procedure)

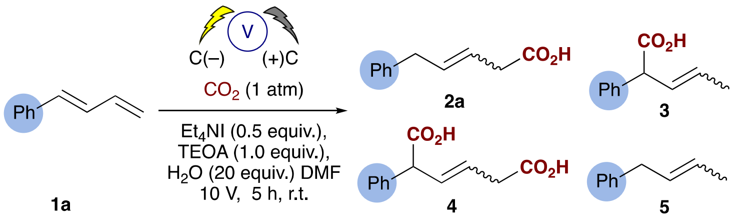

(*E*)-buta-1,3-dien-1-ylbenzene (**1a**). (130 mg, 1.0 mmol) was added to a solution of Et_4NI (128 mg, 0.5 mmol) and TEOA (1.0 mmol) in DMF (5 mL). The resulting mixture was flushed with CO_2 for 10 min and then electrolysed at a constant voltage of 10 V (current fluctuated from 60-100 mA) in a single compartment cell containing a carbon cathode and carbon anode with a constant CO_2 flow with stirring for 5h. The crude reaction mixture was acidified by addition of HCl/H_2O (1:1, 2 mL) and extracted with diethyl ether (3 x 5 mL) to afford an amber oil. ^{1}H NMR analysis showed the presence of three major components **2a**, **3** and **4** in a ratio of 1.7: 1: 1 and amount of reduced product **5** around 10% (detected by GCMS).

4.0 Optimisation Screening

Electrode screening: The above procedure has been repeated by using different electrodes and the product analysed by GCMS.

Entry	Anode	Cathode	Ratio **2a** : **3** : **4** : **5**
1	C	C	1.7 : 1 : 1 : 1
2	C	Ni	0 : 0 : 0 : 1
3	C	Cu	3.8 : 1 : 1 : 2
4	C	SS	9 : 1 : 1 : 1
5	SS	SS	3.1 : 1 : 1 : 0

Water additive screen: The above procedure has been repeated but using a graphite electrode at anode and stainless steel at cathode with different amount of water.

Entry	Water amount	Ratio **2a** : **3** : **4** : **5**
1	5 eq.	9 : 1 : 0.82 : 0.7
2	10 eq.	9 : 1 : 0.74 : 0.7
3	15 eq.	9 : 1 : 0.69 : 0.65
4	20 eq.	9 : 1 : 0.51 : 0.6
5	1 mL	0

5.0 Optimised Procedure Used

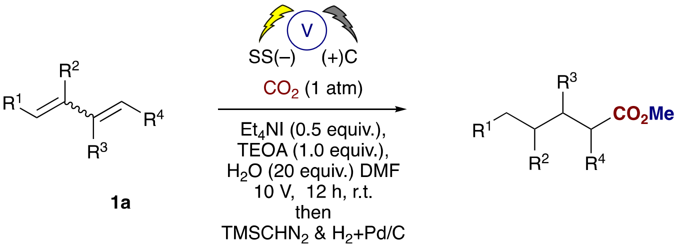

General Procedure II: Substrate (1.0 mmol) was added to a solution of Et_4NI (128 mg, 0.5 mmol) and TEOA (149 mg, 1.0 mmol) in DMF (5 mL) and H_2O (360 μL, 20.0 mmol). The resulting mixture was flushed with CO_2 and kept under a positive pressure of CO_2. The mixture was electrolysed at a constant voltage of 10V (current fluctuated from 60-100 mA) in a single compartment cell containing a stainless-steel cathode and carbon anode with stirring for 4h for substrates **1a-i**. The crude reaction mixture was acidified by addition of HCl/H_2O (1:1, 2 mL) and extracted with diethylether (3 x 5mL). The combined organic phases were washed with brine, dried over $MgSO_4$ and filtered. The solvent was then removed under vacuum and it was dissolved in a 1:1 $MeOH:Et_2O$ mixture. 1 mL of trimethylsilyldiazomethane 2M solution in hexane was added and the reaction was stirred and after 30 minutes silica gel was added, and solvent was removed under vacuum. The monoacids were purified by column chromatography (hexane/EtOAc mixtures), and the product was directly reduced by mixing it with 10% Pd/C (10.6 mg, 5 mol%) in 50 mL Methanol and stirred at room temperature for 24 h under 50 psi of hydrogen gas flow. The solution was filtered through celite and the solvent was removed under reduced pressure and the preparative TLC was used to separate the two acids.

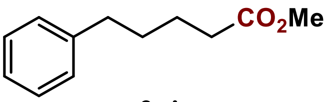

2a'

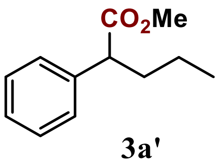

3a'

Methyl 5-phenylpentanoate (2a') and Methyl 2-phenylpentanoate (3a') General procedure II was followed using (*E*)-buta-1,3-dien-1-ylbenzene (**1a**) (130.0 mg, 1.0 mmol), affording **2a'** and **3a'** (100.0 mg, 56.8% yield with ratio 9:1 respectively). Colorless oil.

^{1}H NMR (400 MHZ, $CDCl_3$)
for **2a'** δ 7.28 – 7.14 (m, 5H), 3.65 (s, 3H), 2.61 (t, J = 7.2 Hz, 2H), 2.32 (t, J = 6.8 Hz, 2H), 1.68 – 1.60 (m, 4H).
for **3a'** δ 7.30 - 7.15 (m, 5H), 3.63 (s, 3H), 3.54 (t, J = 7.6 Hz, 1H), 2.02 (m, 1H), 1.73 (m, 1H), 1.25 (m, 2H), 0.89 (t, J = 7.6 Hz, 3H).

^{13}C NMR (101 MHz, $CDCl_3$)
for **2a'** δ 174.1, 142.2, 128.4, 128.3, 125.8, 51.5, 35.6, 34.0, 30.9, 24.6.
for **3a'** δ 174.7, 139.3, 128.6, 128.0, 127.2, 51.9, 51.4, 35.7, 20.8, 13.8.

Spectral data is in agreement with the literature.[11, 12]

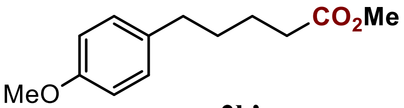

2b’

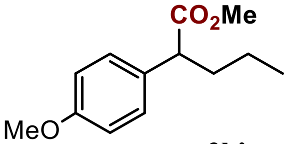

3b’

Methyl 5-(4-methoxyphenyl)pentanoate (2b’) and Methyl 2-(4-methoxyphenyl)pentanoate (3b’). General procedure II was followed using (*E*,*Z*)-1-(buta-1,3-dien-1-yl)-4-methoxybenzene (**1b**) (160.0 mg, 1.0 mmol), affording **2b’** & **3b’** (120.0 mg, 58.2% yield with ratio 8.2:1 respectively). Colorless oil.

^{1}H NMR (400 MHZ, $CDCl_3$)
for **2b’** δ 7.07 (d, *J* = 8.4 Hz, 2H), 6.81 (d, *J* = 8.8 Hz, 2H), 3.77 (s, 3H), 3.64 (s, 3H), 2.55 (t, *J* = 7.2 Hz, 2H), 2.31 (t, *J* = 6.8 Hz, 2H), 1.65 – 1.58 (m, 4H).
for **3b’** δ 7.20 (d, *J* = 8.4 Hz, 2H), 6.83 (d, *J* = 8.4 Hz, 2H), 3.77 (s, 3H), 3.63 (s, 3H), 3.49 (t, *J* = 7.6 Hz, 1H), 2.00 (m, 1H), 1.71 (m, 1H), 1.22 (m, 2H), 0.88 (t, *J* = 7.2 Hz, 3H).

^{13}C NMR (101 MHz, $CDCl_3$)
for **2b’** δ 174.2, 157.8, 134.2, 129.3, 113.8, 55.3, 51.5, 34.6, 34.0, 31.2, 24.6.
for **3b’** δ 175.0, 158.7, 131.4, 128.9, 114.0, 55.3, 51.9, 50.5, 35.7, 20.7, 13.8.

IR (neat, cm^{-1})
for **3b’** 2959, 1733, 1611, 1512, 1436, 1257, 1100, 1034, 829, 794.

HRMS ESI,
for **3b’** ($C_{13}H_{18}NaO_3$) $[M+Na]^+$ *calculated* 245.1154, *found* 245.1151.

Spectral data for **2b’** is in agreement with the literature.[10]

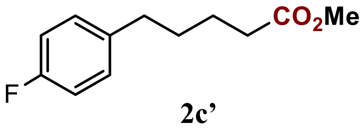

2c’

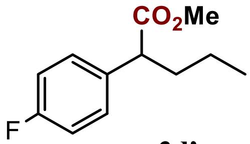

3d’

Methyl 5-(4-flourophenyl)pentanoate (2c’) and Methyl 2-(4-flourophenyl)pentanoate (3c’). General procedure II was followed using (*E*,*Z*)-1-(buta-1,3-dien-1-yl)-4-fluorobenzene (**1c**) (148.0 mg, 1.0 mmol), affording **2c’** & **3c’** (98.0 mg, 50.5% yield with ratio 6.2:1 respectively). Colorless oil.

^{1}H NMR (400 MHZ, $CDCl_3$)
for **2c’** δ 7.10 (dd, *J* = 8.8, 5.6, 2.8 Hz, 2H), 6.94 (t, *J* = 8.8 Hz, 2H), 3.65 (s, 3H), 2.58 (t, *J* = 7.2 Hz, 2H), 2.31 (t, *J* = 6.8 Hz, 2H), 1.68 – 1.58 (m, 4H).
for **3c’** δ 7.27 (d, *J* = 5.2 Hz, 2H), 6.99 (d, *J* = 8.4 Hz, 2H), 3.64 (s, 3H), 3.52 (t, *J* = 7.6 Hz, 1H), 2.01 (m, 1H), 1.69 (m, 1H), 1.26 (m, 2H), 0.89 (t, *J* = 7.2 Hz, 3H).

^{13}C NMR (100 MHz, $CDCl_3$)
for **2c’** δ 174.0, 137.7 (d, *J* = 3.8 Hz), 129.7 (d, *J* = 7.6 Hz), 115.1 (d, *J* = 21.0 Hz), 51.5, 34.8, 33.9, 31.0, 24.5.
for **3c’** δ 174.5, 134.9, 129.4, 115.5, 115.3, 52.0, 50.6, 35.8, 20.7, 13.8.

^{19}F NMR (375 MHz, $CDCl_3$)
for **2c’** δ -117.7.

for **3c’** δ -115.5.

IR (neat, cm^{-1})
for **2c’** 2948, 1736, 1601, 1509, 1436, 1363, 1219, 1157, 1097, 823.
for **3c’** 2961, 1733, 1643, 1510, 1463, 1260, 1095, 1019, 798.

HRMS ESI,
for **2c’** ($C_{12}H_{15}FNaO_2$) $[M+Na]^+$ *calculated* 233.0954, *found* 233.0951.
for **3c’** ($C_{12}H_{15}FNaO_2$) $[M+Na]^+$ *calculated* 233.0954, *found* 233.0955.

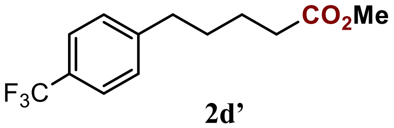

2d’

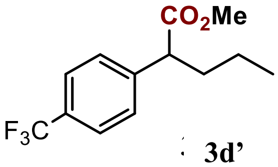

3d’

Methyl 5-(4-triflouromethylphenyl)pentanoate (2d’) and Methyl 2-(4-triflouromethylphenyl)pentanoate (3d’). General procedure II was followed using (*E,Z*)-1-(buta-1,3-dien-1-yl)-4-(trifluoromethyl)benzene (**1d**) (198.0 mg, 1.0 mmol), affording **2d’** & **3d’** (110.0 mg, 45.0% yield with ratio 1:5.5 respectively). Colorless oil.

^{1}H NMR (500 MHZ, $CDCl_3$)
for **2d’** δ 7.47 (d, *J* = 8.5 Hz, 2H), 7.22 (d, *J* = 8.0 Hz, 2H), 3.60 (s, 3H), 2.63 (t, *J* = 7.0 Hz, 2H), 2.28 (t, *J* = 7.0 Hz, 2H), 1.63 – 1.58 (m, 4H).
for **3d’** δ 7.56 (d, *J* = 8.0 Hz, 2H), 7.41 (d, *J* = 8.0 Hz, 2H), 3.65 (s, 3H), 3.61 (t, *J* = 8.0 Hz, 1H), 2.03 (m, 1H), 1.75 (m, 1H), 1.26 (m, 2H), 0.90 (t, *J* = 7.5 Hz, 3H).

^{13}C NMR (125 MHz, $CDCl_3$)
for **2d’** δ 173.9, 146.2, 128.7, 125.3, 125.3, 125.2, 51.6, 35.4, 33.8, 30.6, 24.5.
for **3d’** δ 173.9, 143.2, 129.7, 128.4, 125.6, 125.5, 52.1, 51.2, 35.6, 20.7, 13.7.

^{19}F NMR (475 MHz, $CDCl_3$)
for **2d’** δ -62.4.
for **3d’** δ -62.4.

IR (neat, cm^{-1})
for **2d’** 2918, 1732, 1673, 1463, 1325, 1161, 1125, 1067, 1018, 801.
for **3d’** 2923, 1737, 1619, 1462, 1419, 1165, 1126, 1068, 1019, 800.

HRMS ESI,
for **2d’** ($C_{13}H_{15}F_3NaO_2$) $[M+Na]^+$ *calculated* 283.0922, *found* 283.0919.
for **3d’** ($C_{13}H_{15}F_3NaO_2$) $[M+Na]^+$ *calculated* 283.0922, *found* 283.0921.

Spectral data is in agreement with the literature.[8,9]

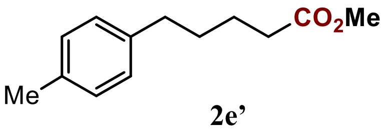

2e’

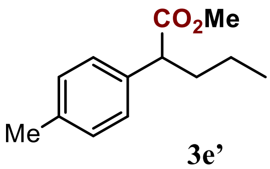

3e’

Methyl 5-(*p*-tolyl)pentanoate (2e’) and Methyl 2-(*p*-tolyl)pentanoate (3e’). General procedure II was followed using (*E,Z*)-1-(buta-1,3-dien-1-yl)-4-methylbenzene (**1e**) (144.0 mg, 1.0 mmol), affording **2e’**& **3e’** (108.0 mg, 56.8% yield with ratio 7.4:1 respectively). Faint yellow oil.

^{1}H NMR (400 MHZ, $CDCl_3$)

for **2e'** δ 7.07 (d, *J* = 8.0 Hz, 2H), 7.04 (d, *J* = 8.4 Hz, 2H), 3.64 (s, 3H), 2.57 (t, *J* = 7.6 Hz, 2H), 2.31 (t, *J* = 7.2 Hz, 2H), 2.30 (s, 3H), 1.67 – 1.57 (m, 4H).
for **3e'** δ 7.17 (d, *J* = 8.0 Hz, 2H), 7.10 (d, *J* = 8.0 Hz, 2H), 3.62 (s, 3H), 3.50 (t, *J* = 7.2 Hz, 1H), 2.31 (s, 3H), 2.00 (m, 1H), 1.72 (m, 1H), 1.25 (m, 2H), 0.88 (t, *J* = 7.6 Hz, 3H).

^{13}C NMR (101 MHz, $CDCl_3$)
for **2e'** δ 174.2, 139.1, 135.2, 129.0, 128.3, 51.5, 35.1, 34.0, 31.0, 24.6, 21.0.
for **3e'** δ 174.8, 136.8, 136.3, 129.3, 127.8, 51.9, 51.0, 35.6, 21.1, 20.7, 13.8.

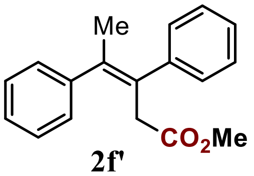

2f'

Methyl 3,4-diphenylpent-3-enoate (2f'). General procedure II was followed (without hydrogenation step) using 2,3-Diphenyl-1,3-butadiene (**1f**) (206.0 mg, 1.0 mmol), affording **2f'** (150.0 mg, 59.5% yield). Colorless solid.

^{1}H NMR (400 MHZ, $CDCl_3$) δ 7.36 – 7.24 (m, 10H), 3.52 (s, 3H), 3.26 (s, 2H), 1.89 (s, 3H).

^{13}C NMR (101 MHz, $CDCl_3$) δ 172.3, 143.5, 141.9, 138.0, 130.2, 128.8, 128.4, 128.2, 127.9, 126.9, 126.8, 51.6, 41.1, 22.8.

IR (neat, cm^{-1}) 2918, 1736, 1600, 1492, 1436, 1163, 1015, 701.

HRMS ESI, ($C_{18}H_{18}NaO_2$) $[M+Na]^+$ *calculated* 289.3298, *found* 289.3299.

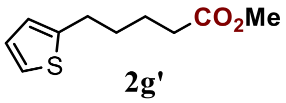

2g'

Methyl 5-(thiophen-2-yl)pentanoate (2g'). General procedure II was followed using (*E*,*Z*)-2-(buta-1,3-dien-1-yl)thiophene (**1g**) (136.0 mg, 1.0 mmol), affording **2g'** (100.0 mg, 54.9% yield). Yellow oil.

^{1}H NMR (400 MHZ, $CDCl_3$) δ 7.05 (dd, *J* = 4.8, 1.2 Hz, 1H), 6.85 (dd, *J* = 5.2, 3.6 Hz, 1H), 6.72 (d, *J* = 4.4 Hz, 1H), 3.60 (s, 3H), 2.78 (t, *J* = 7.2 Hz, 2H), 2.28 (t, *J* = 7.2 Hz, 2H), 1.70 – 1.60 (m, 4H).

^{13}C NMR (101 MHz, $CDCl_3$) δ 173.0, 143.9, 125.7, 123.2, 121.9, 50.5, 32.8, 30.2, 28.5, 23.3.

Spectral data is in agreement with the literature.[10]

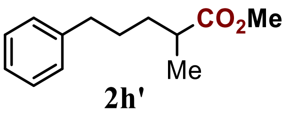

2h'

Methyl 2-methyl-5-phenylpentanoate (2h'). General procedure II was followed using (*E*,Z)-Penta-1,3-dien-1-ylbenzene (**1h**) (144.0 mg, 1.0 mmol), affording **2h'** (110.0 mg, 57.8% yield). Colorless oil.

^{1}H NMR (500 MHZ, $CDCl_3$) δ 7.22 – 7.09 (m, 5H), 3.59 (s, 3H), 2.54 (t, *J* = 7.5 Hz, 2H), 2.40 (six, *J* = 7.0 Hz, 1H), 1.68 – 1.60 (m, 1H), 1.58 – 1.51 (m, 2H), 1.43 – 1.36 (m, 1H), 1.08 (d, *J* = 7.5 Hz, 3H).

^{13}C NMR (125 MHz, $CDCl_3$) δ 177.2, 142.2, 128.4, 128.3, 125.8, 51.5, 39.4, 35.8, 33.4, 29.1, 17.1.

Spectral data is in agreement with the literature.[9]

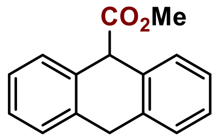

2j'

Methyl 9,10-dihydroanthracene-10-carboxylate (2j'). General procedure II was followed (without hydrogenation step) using Anthracene (178.1 mg, 1.0 mmol), affording **2j'** (140.0 mg, 62.5% yield). White solid.

^{1}H NMR (500 MHZ, $CDCl_3$) δ 7.40 – 7.23 (m, 8H), 5.00 (s, 1H), 4.32 (d, *J* = 18.5 Hz, 1H), 3.90 (d, *J* = 18.0 Hz, 1H), 3.58 (s, 3H).

^{13}C NMR (125 MHz, $CDCl_3$) δ 172.4, 136.7, 133.8, 128.3, 128.1, 127.5, 126.4, 52.9, 52.4, 35.7.

Spectral data is in agreement with the literature.[15]

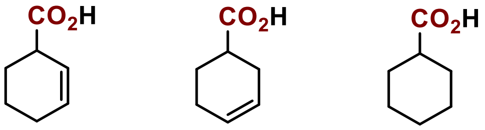

2ma 2mb 2m'

2-Cyclohexene-1-carboxylic acid (2ma) and 3-Cyclohexene-1-carboxylic acid (2mb). General procedure II was followed using Cyclohexa-1,3-diene (**1m**) (80.0 mg, 1.0 mmol), affording **2ma** & **2mb** (100.0 mg, 79.3% yield with ratio 1.65:1 respectively) without methylation and hydrogenation steps. Colorless oil. And after hydrogenation step, **Cyclohexane carboxylic acid (2m')** was obtained.

^{1}H NMR (400 MHZ, $CDCl_3$)
for **2ma&2mb** δ 10.93 (br, 1H), 5.89-5.64 (m, 2H), 3.12 (m, 1H, **3ka** C-H), 2.60 (m, 1H, **3kb** C-H), 2.26-1.53 (m, 6H).
for **2m'** δ 9.05 (br, 1H), 2.26 (m, 1H), 1.92-1.15 (m, 10H).

^{13}C NMR (101 MHz, $CDCl_3$)
for **2ma&2mb** δ 182.4, 181.2, 130.2, 126.8, 125.0, 123.6, 40.9, 39.1, 27.2, 25.1, 24.8, 24.6, 24.3, 20.7.
for **2m'** δ 182.2, 43.2, 28.9, 25.8, 25.4.

Spectral data is in agreement with the literature. [16,17,18]

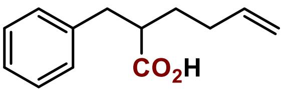

7

2-Benzylhex-5-enoic Acid (7). General procedure II was followed (without hydrogenation and methylation steps) using (*E,Z*)-1,5-Hexadien-1-ylbenzene (**6**) (158.1 mg, 1.0 mmol), affording **7** (150.0 mg, 73.5% yield). Colorless liqiud.

^{1}H NMR (400 MHZ, $CDCl_3$) δ 11.22 (br, 1H), 7.32 – 7.16 (m, 5H), 5.73 (m, 1H), 4.98 (m, 2H), 2.97 (m, 1H), 2.73 (m, 2H), 2.11 (m, 2H), 1.78 (m, 1H), 1.74 (m, 1H).

^{13}C NMR (100 MHz, $CDCl_3$) δ 181.1, 138.9, 137.5, 128.9, 128.5, 126.5, 115.5, 46.7, 38.1, 31.4, 30.7.

Spectral data is in agreement with the literature.[20]

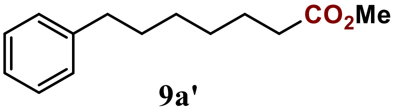

9a'

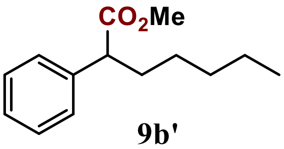

9b'

Methyl 7-phenylheptanoate (9a') and Methyl 2-phenylheptanoate (9b'). General procedure II was followed using (*1E,3(E,Z)*)-(hexa-1,3,5-trien-1-yl)benzene (**8**) (156.0 mg, 1.0 mmol), affording **9a'** & **9b'** (120.0 mg, 59.4% yield with ratio 6:1 respectively). Colorless oil.

^{1}H NMR (400 MHZ, $CDCl_3$)
for **9b'** δ 7.37 - 7.27 (m, 5H), 3.69 (s, 3H), 3.57 (t, *J* = 7.6 Hz, 1H), 2.09 (m, 1H), 1.80 (m, 1H), 1.25 (m, 6H), 0.89 (t, *J* = 6.8 Hz, 3H).
for **9a'** δ 7.24 – 7.09 (m, 5H), 3.60 (s, 3H), 2.53 (t, *J* = 7.2 Hz, 2H), 2.23 (t, *J* = 7.6 Hz, 2H), 1.59 – 1.52 (m, 4H), 1.30 – 1.26 (m, 4H).

^{13}C NMR (101 MHz, $CDCl_3$)
for **9b'** δ 174.7, 139.3, 128.6, 127.9, 127.2, 51.9, 51.7, 33.6, 31.6, 27.3, 22.5, 14.0.
for **9a'** δ 174.3, 142.7, 128.4, 128.3, 125.6, 51.5, 35.9, 34.1, 31.3, 29.0, 28.9, 24.9.

Spectral data is in agreement with the literature.[13,14]

Deuterium Labelling Studies

Following the general procedure II but using D_2O instead of H_2O and starting with Anthracene affording the deuterated product **(D)2j'.**

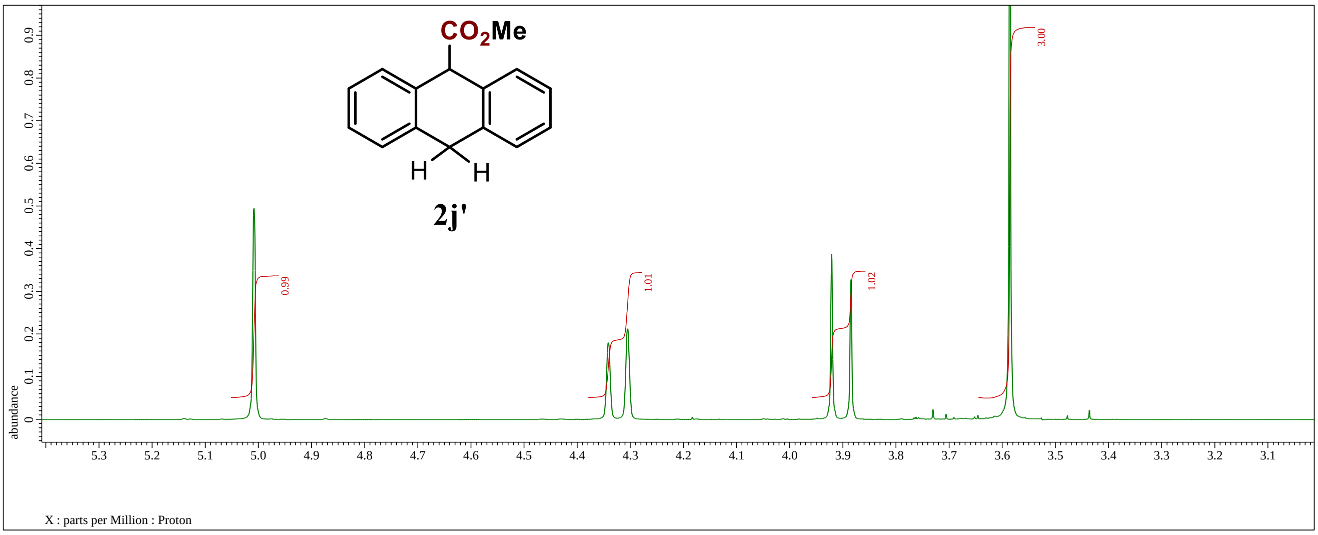

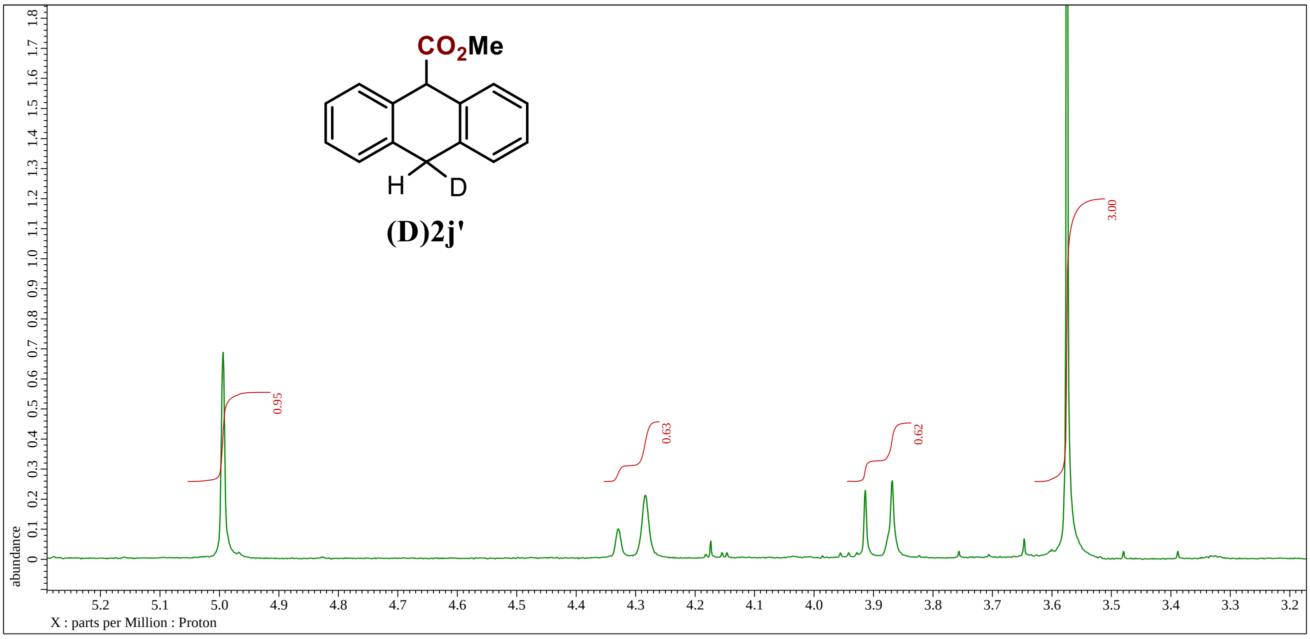

Following the general procedure II but using D_2O or H_2O and no CO_2 starting with Anthracene affording the reduced product **5J** and deuterated reduced product **(D)5J**.

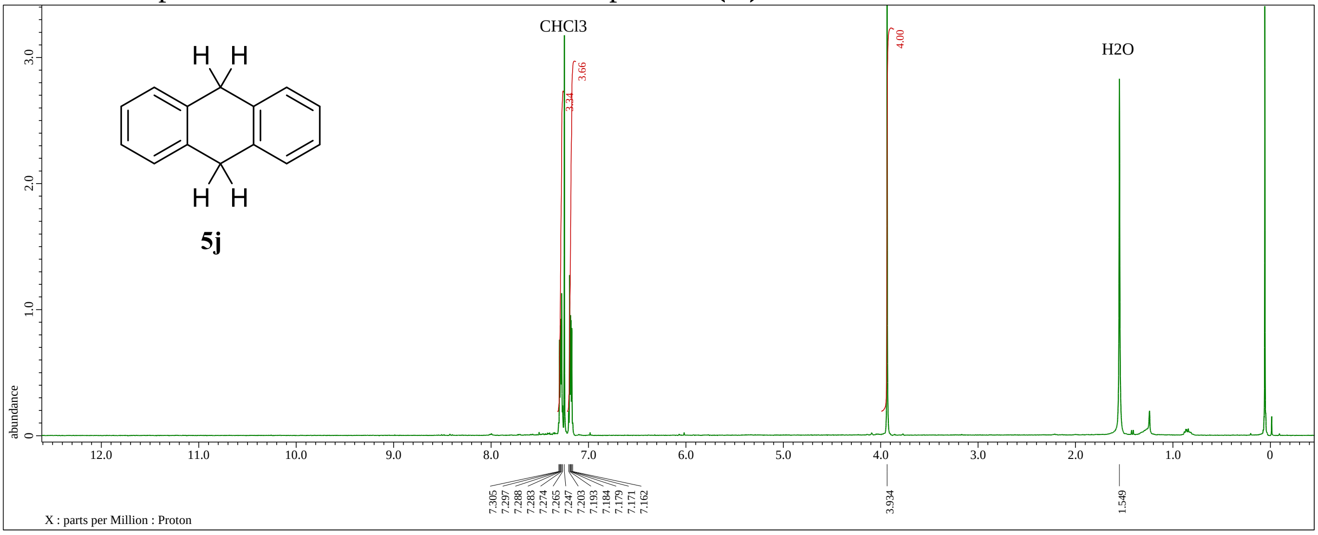

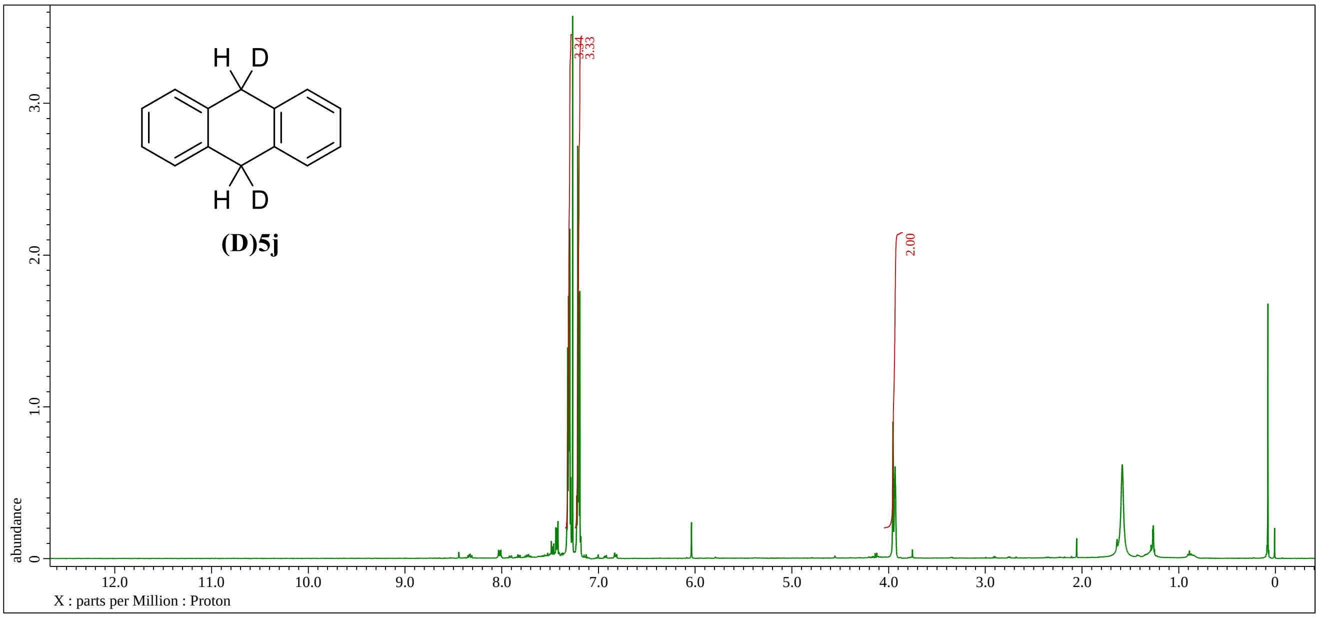

Unsuccessful substrates

The following compounds were tested under the optimized reaction conditions. In all cases, we observed no conversion to product or less than 30% yield of the corresponding carboxylic acid derivatives.

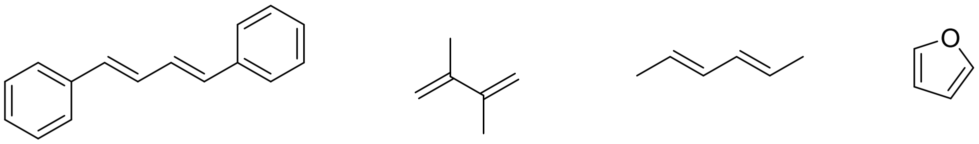

References:

(1) Mundal, D.; Lutz, K.; Thomson R. *Org. Lett.*, **2009**, 11, 465.

(2) Menges, F. S.; Craig, S. M.; Tötsch, N.; Bloomfield, A.; Ghosh, S.; Krüger, H. J.;Johnson, M. A. Angew. *Chemie. Int. Ed.* **2016,** 55, 1282–1285.

(3) Takimoto, M.; Mori, M. *J. Am. Chem. Soc.* **2001**, 123, 2895–2896.

(4) Yamashita, M.; Hirano, K.; Satoh,T.; Miura, M. Adv. *Synth. Catal.* **2011,** 353, 631.

(5) Madden, K. S.; David, S.; Knowles, J. P.; Whiting, A. *Chem. Commun.* **2015,** 51, 11409-11412.

(6) Ohashi, M.; Takeda, I.; Ikawa, M.; Ogoshi, S. *J. Am. Chem. Soc.* **2011**, 133, 18018-18021.

(7) Wang, R.; Zhang, S. *RSC Advances* **2014,** 4, 39497-39507.

(8) Estévez, M.-C.; Galve, R.; Sánchez-Baeza, F.; Marco, M.-P. *Anal. Chem.* **2005,** *77*, 5283.

(9) Paritosh, S.; Hsu, Yun-Chu, H.; Cheng, Chien-Hong, C. *J. Org. Chem.* **2006,** 2, 655.

(10) Yan, Xiao-Biao, Y.; Li, Chun-Ling, L.; Jin, Wen-Jie, J.; Guo, Peng, G.; Xing-Zhong, S. *Chem. Sci.* **2018,** 9, 4529.

(11) Lee, J. *J. Korean Chem. Soc.* **2017,** 61, 125.

(12) Lu, Q.; Xiao, X.; Cui, X. *Faming Zhuanli Shenqing* **2019,** CN 109336763A.

(13) Xue, F.; Wang, F.; Liu, J.; Di, J.; Liao, Q.; Lu, H.; Zhu, M.; He, L.; He, H.; Zhang, D.; Song, H.; Liu, X.; Qin, Y. *Angew. Chem.* **2018,** 57, 6667.

(14) Guisán-Ceinos, M.; Soler-Yanes, R.; Collado-Sanz, D.; Phapale, V. B.; Buñuel, E; Cárdenas, D. J. *Chem. Eur. J.* **2013,** 19, 8405.

(15) Findlay, N. J.; Park, S. R.; Schoenebeck, F.; Cahard, E.; Zhou, S.; Berlouis, L. E. A.; Spicer, M. D.; Tuttle, T.; Murphy, J. A. *J. Am. Chem. Soc.* **2010**, 132, 15462.

(16) Dai, Peng-F.; Qu, Jian-P.; Kang, Yan-B. *Org. Lett.* **2019,** 21, 1393-1396.

(17) Takaya, J.; Sasano, K.; Iwasawa, N. *Org. Lett.* **2011,** 13, 1698–1701.

(18) Yu, H.; S. Rui, G. Dai, Y. Zhai, H. Lin, S. Han, Y. Wei, *Angew. Chem., Int. Ed.* **2017,** 56, 3867.

(19) Ding, F; William, R; Wang, F; Liu, X. *Chem. Comm.* **2012,** 48, 8709-8711.

(20) Hoshikawa, T.; Tanji, K.; Matsuo, J.; Ishibashi, H. *Chem. Pharm. Bull.* **2012,** 60, 4, 548-553.

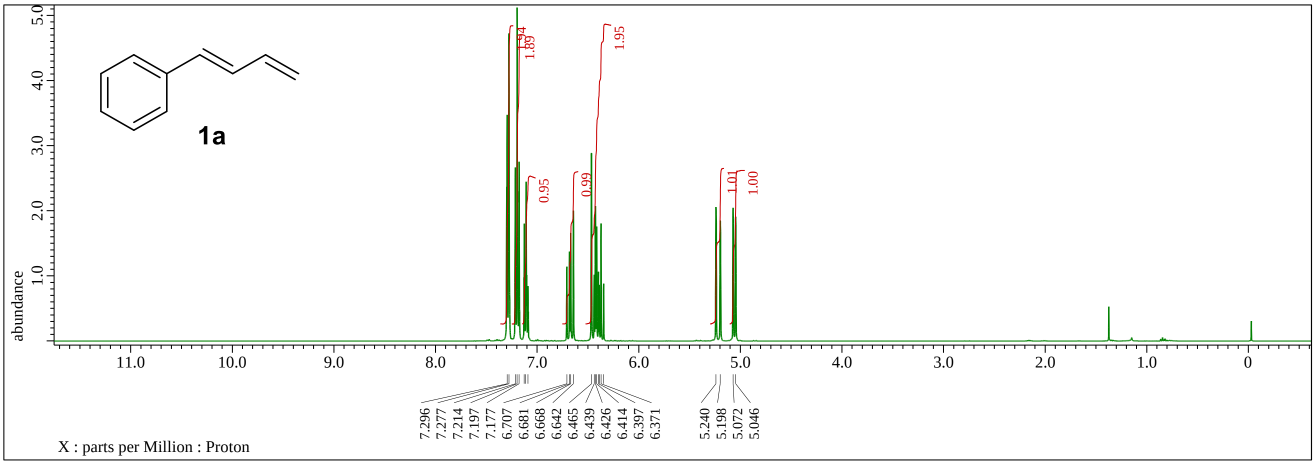
1a
abundance
5.0
4.0
3.0
2.0
1.0
1.94
1.89
0.95
0.99
1.95
1.01
1.00
11.0 10.0 9.0 8.0 7.0 6.0 5.0 4.0 3.0 2.0 1.0 0
7.296 7.277 7.214 7.197 7.177 6.707 6.681 6.668 6.642 6.465 6.439 6.426 6.414 6.397 6.371
5.240 5.198 5.072 5.046
X : parts per Million : Proton

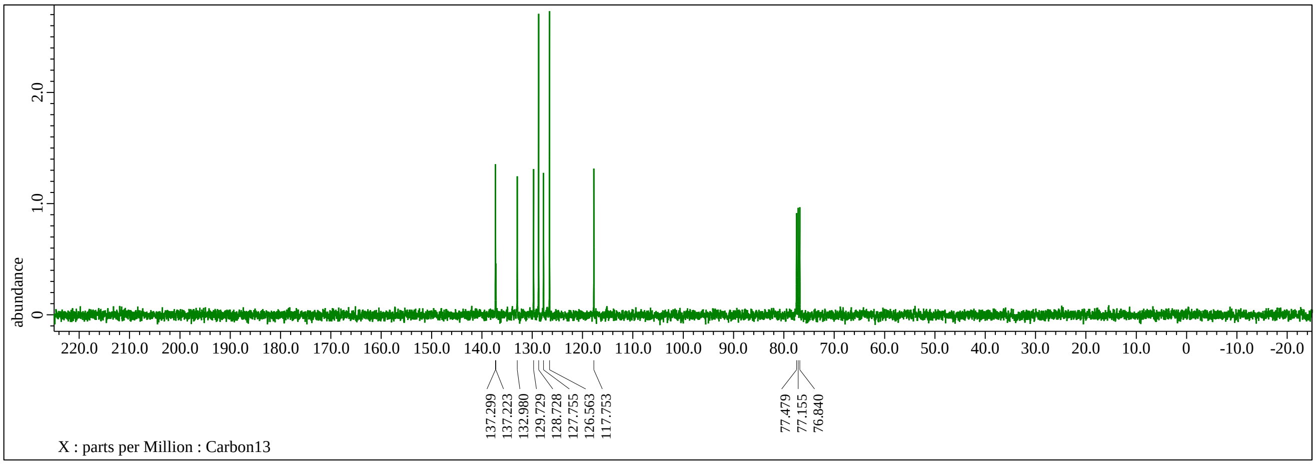
abundance
2.0
1.0
0
220.0 210.0 200.0 190.0 180.0 170.0 160.0 150.0 140.0 130.0 120.0 110.0 100.0 90.0 80.0 70.0 60.0 50.0 40.0 30.0 20.0 10.0 0 -10.0 -20.0
137.299 137.223 132.980 129.729 128.728 127.755 126.563 117.753
77.479 77.155 76.840
X : parts per Million : Carbon13

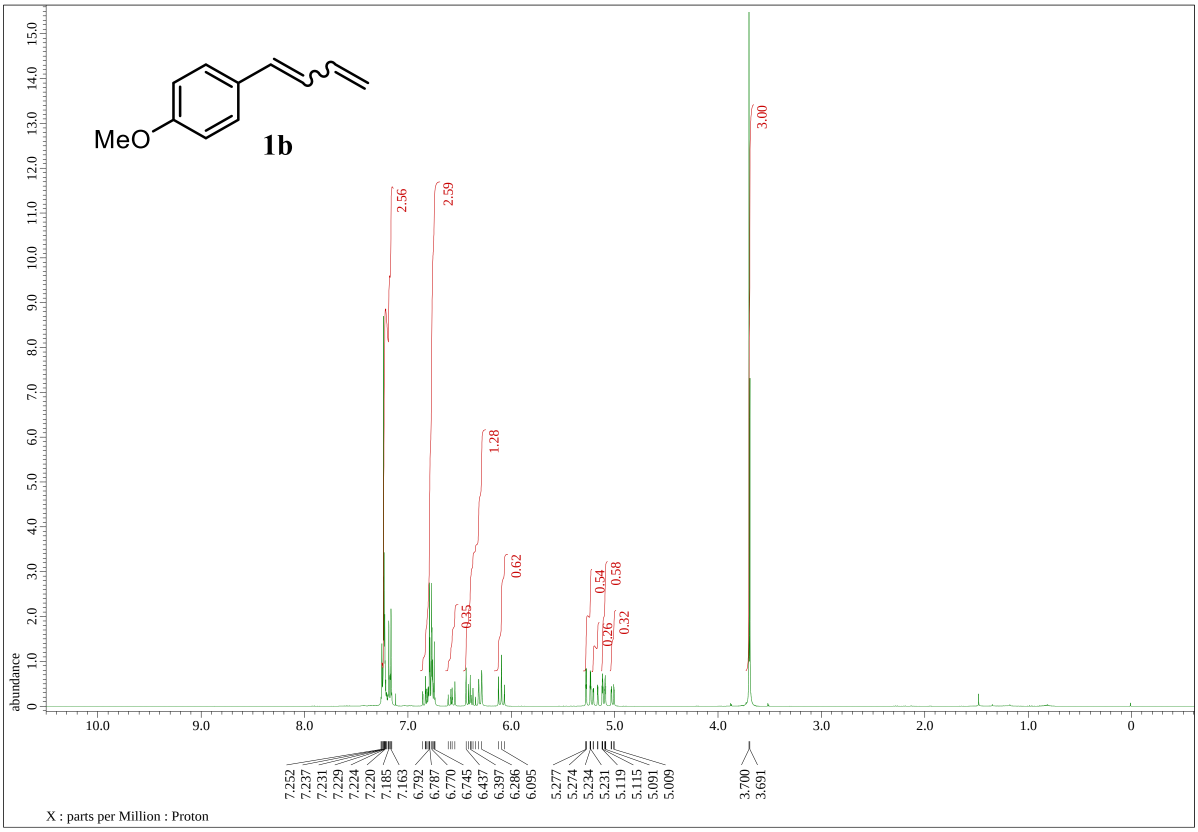
MeO
1b
abundance
2.56
2.59
1.28
0.35
0.62
0.54
0.58
0.26
0.32
3.00
7.252
7.237
7.231
7.229
7.224
7.220
7.185
7.163
6.792
6.787
6.770
6.745
6.437
6.397
6.286
6.095
5.277
5.274
5.234
5.231
5.119
5.115
5.091
5.009
3.700
3.691
X : parts per Million : Proton

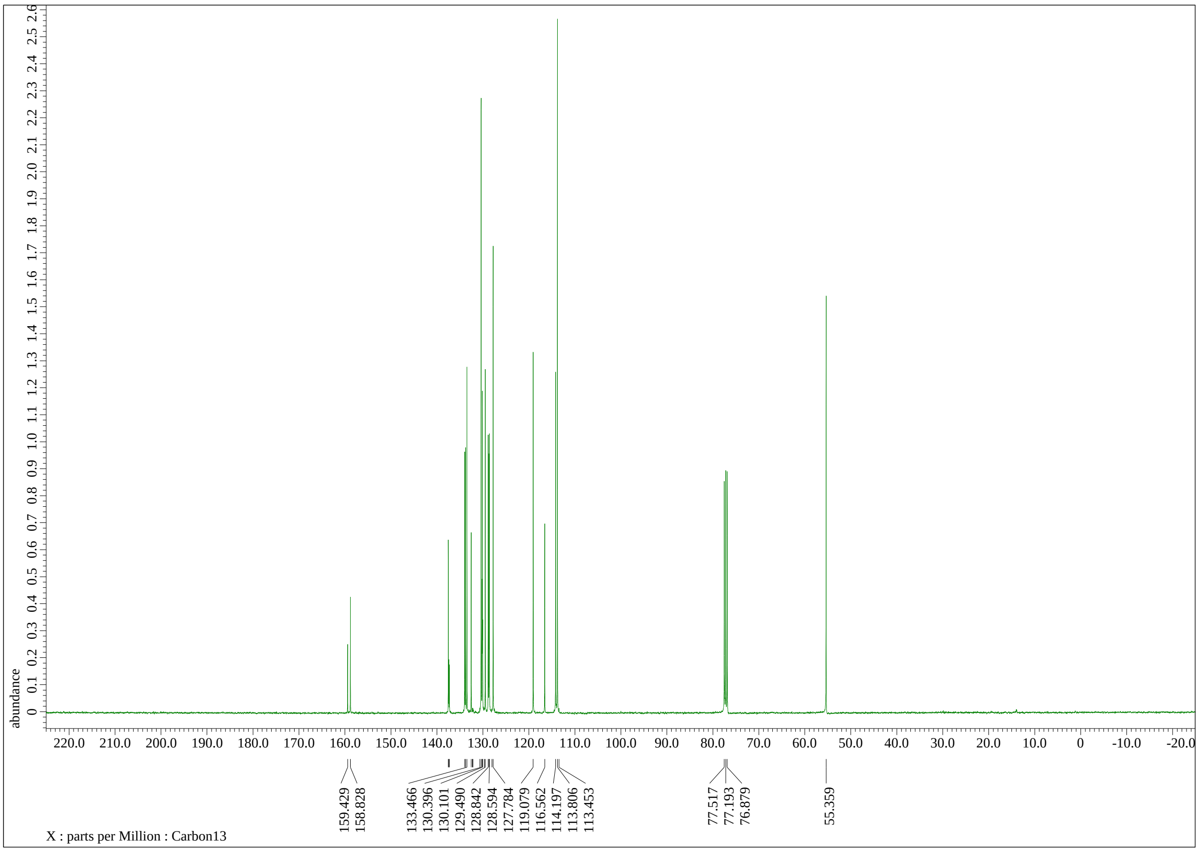
abundance
159.429
158.828
133.466
130.396
130.101
129.490
128.842
128.594
127.784
119.079
116.562
114.197
113.806
113.453
77.517
77.193
76.879
55.359
X : parts per Million : Carbon13

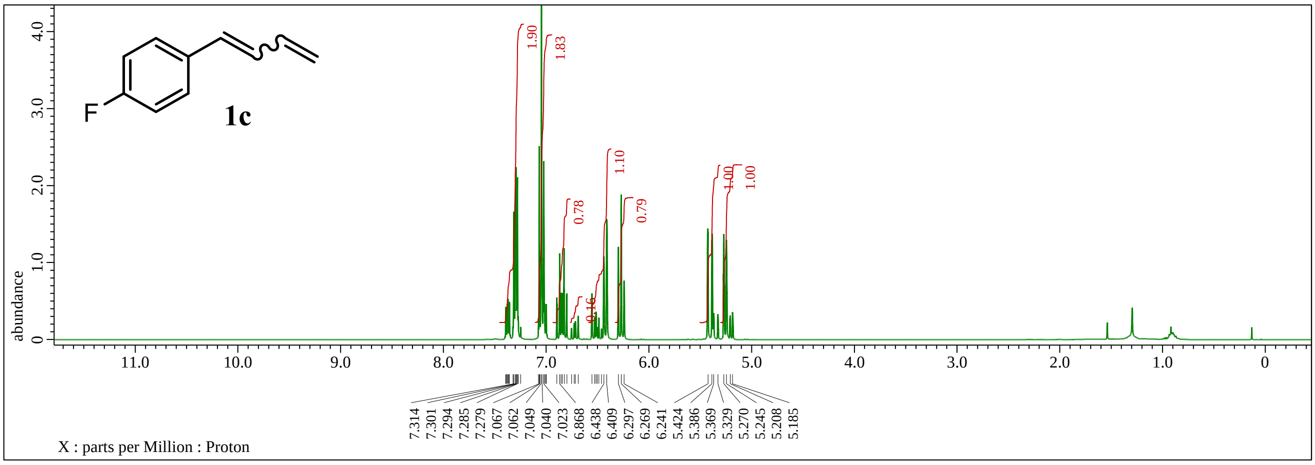
F
1c
1.90
1.83
0.78
0.16
1.10
0.79
1.00
1.00
7.314 7.301 7.294 7.285 7.279 7.067 7.062 7.049 7.040 7.023 6.868 6.438 6.409 6.297 6.269 6.241 5.424 5.386 5.369 5.329 5.270 5.245 5.208 5.185
abundance
X : parts per Million : Proton

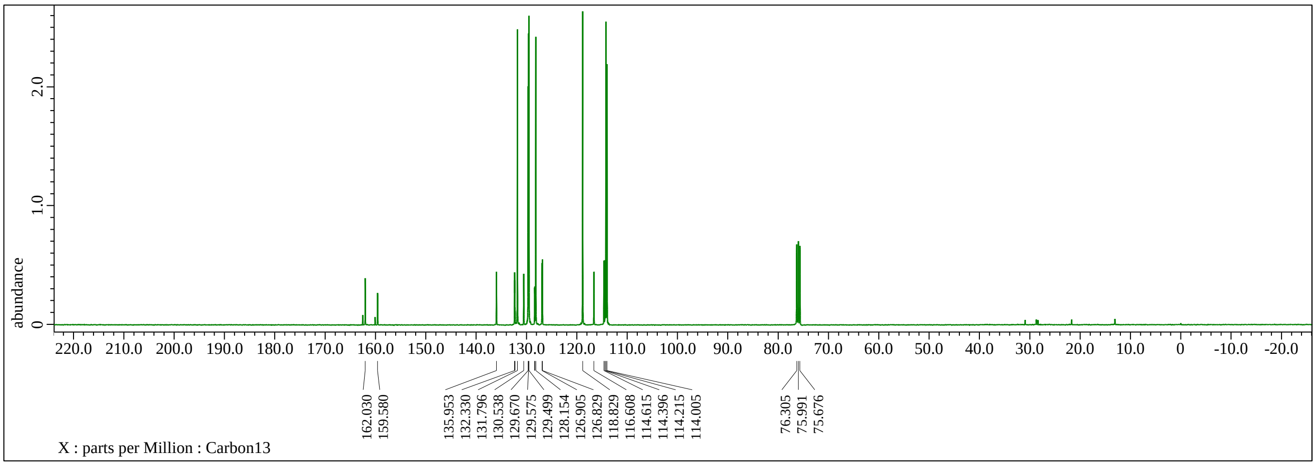
162.030 159.580
135.953 132.330 131.796 130.538 129.670 129.575 129.499 128.154 126.905 126.829 118.829 116.608 114.615 114.396 114.215 114.005
76.305 75.991 75.676
abundance
X : parts per Million : Carbon13

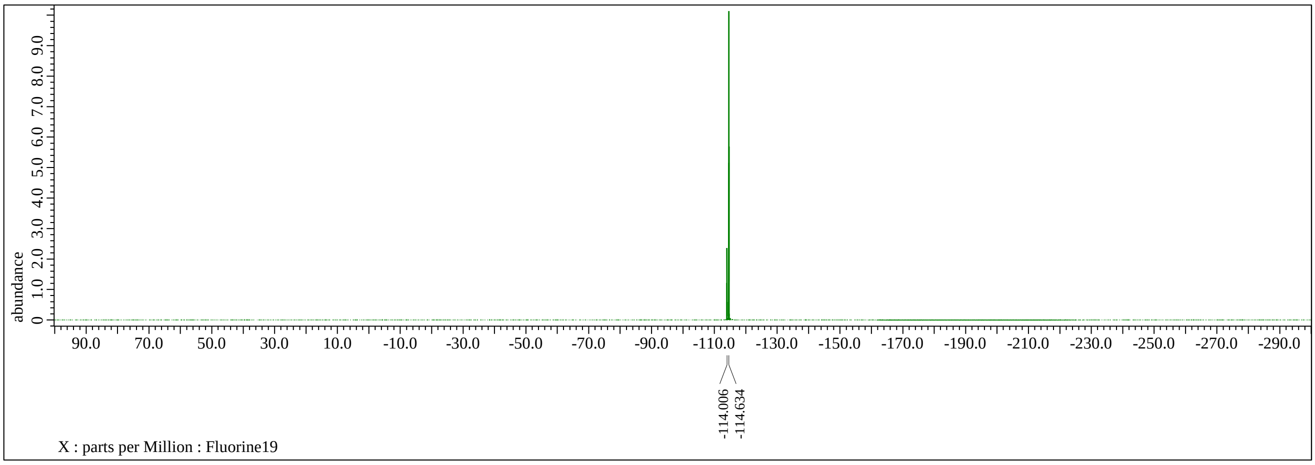
-114.006 -114.634
abundance
X : parts per Million : Fluorine19

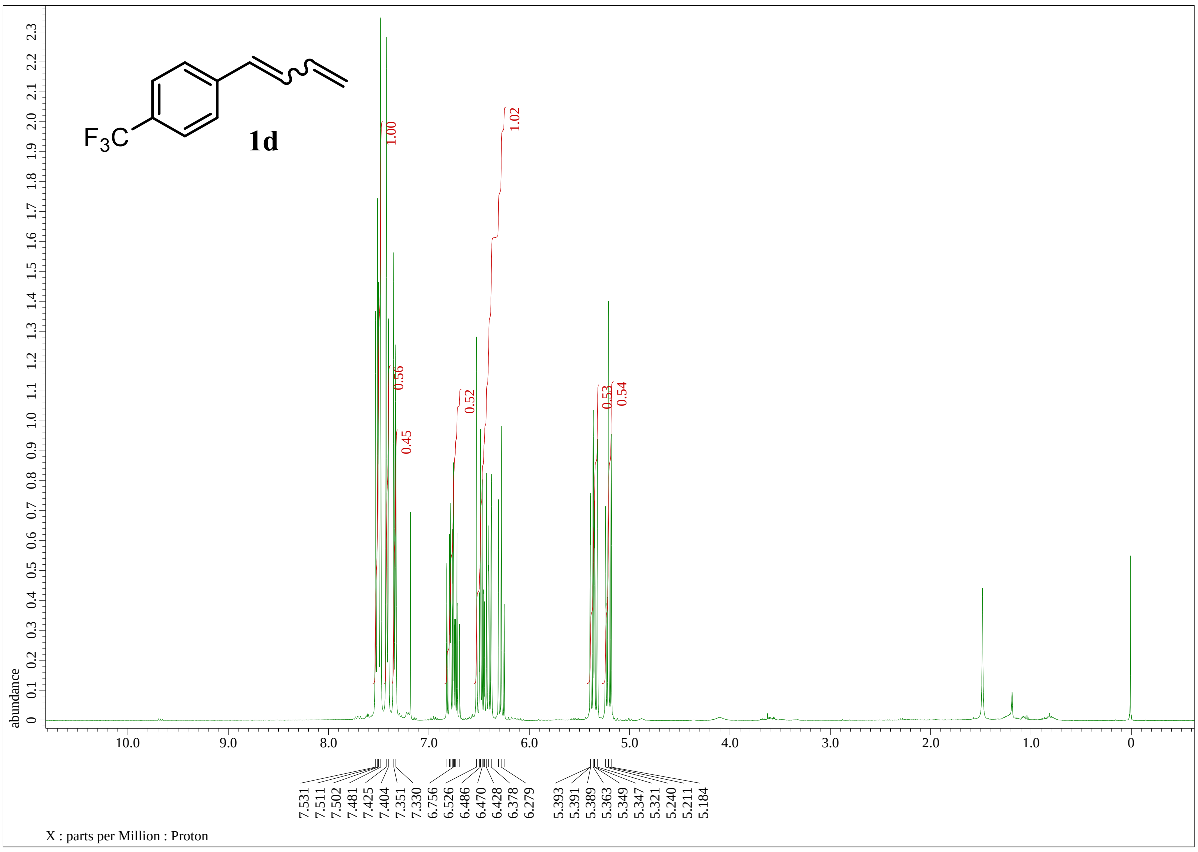
F3C
1d
1.00
0.56
0.45
0.52
1.02
0.53
0.54
abundance
7.531 7.511 7.502 7.481 7.425 7.404 7.351 7.330 6.756 6.526 6.486 6.470 6.428 6.378 6.279
5.393 5.391 5.389 5.363 5.349 5.347 5.321 5.240 5.211 5.184
X : parts per Million : Proton

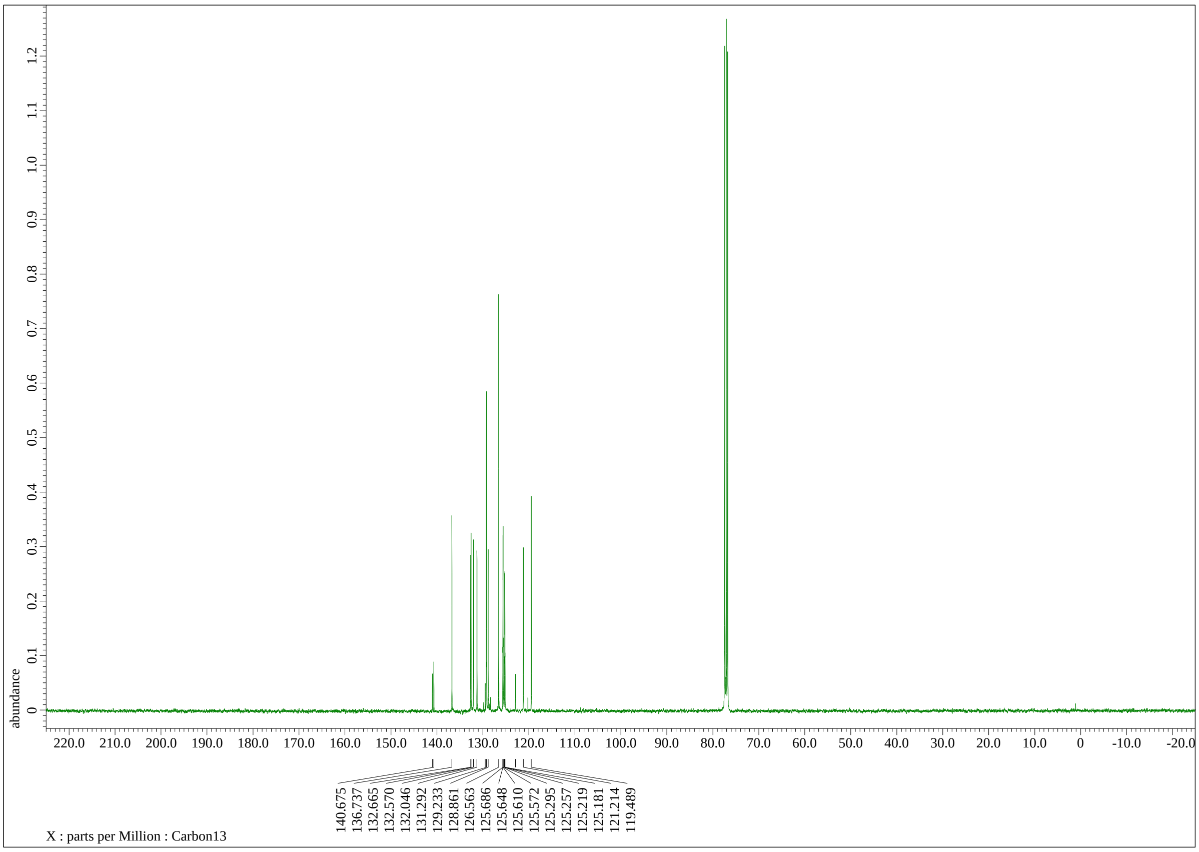
abundance
140.675 136.737 132.665 132.570 132.046 131.292 129.233 128.861 126.563 125.686 125.648 125.610 125.572 125.295 125.257 125.219 125.181 121.214 119.489
X : parts per Million : Carbon13

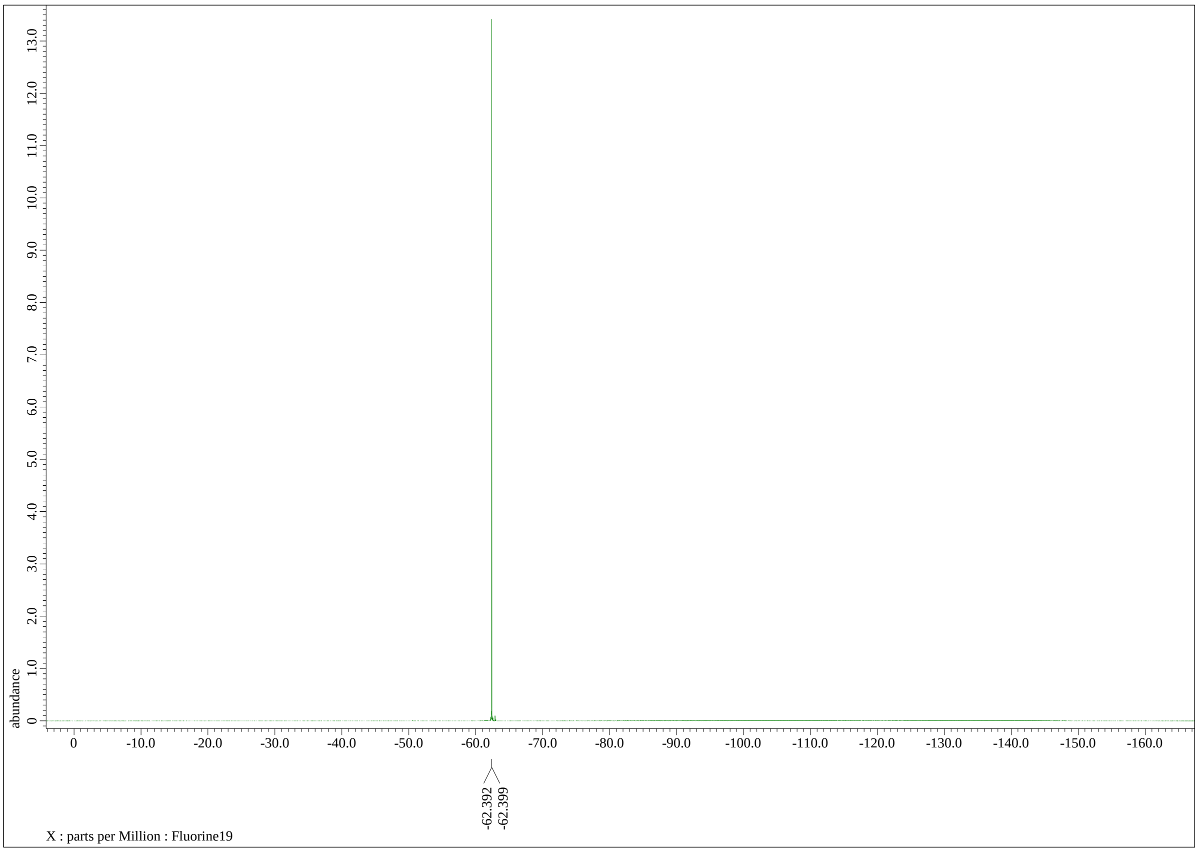

abundance
13.0
12.0
11.0
10.0
9.0
8.0
7.0
6.0
5.0
4.0
3.0
2.0
1.0
0
0
-10.0
-20.0
-30.0
-40.0
-50.0
-60.0
-70.0
-80.0
-90.0
-100.0
-110.0
-120.0
-130.0
-140.0
-150.0
-160.0
-62.392
-62.399
X : parts per Million : Fluorine19

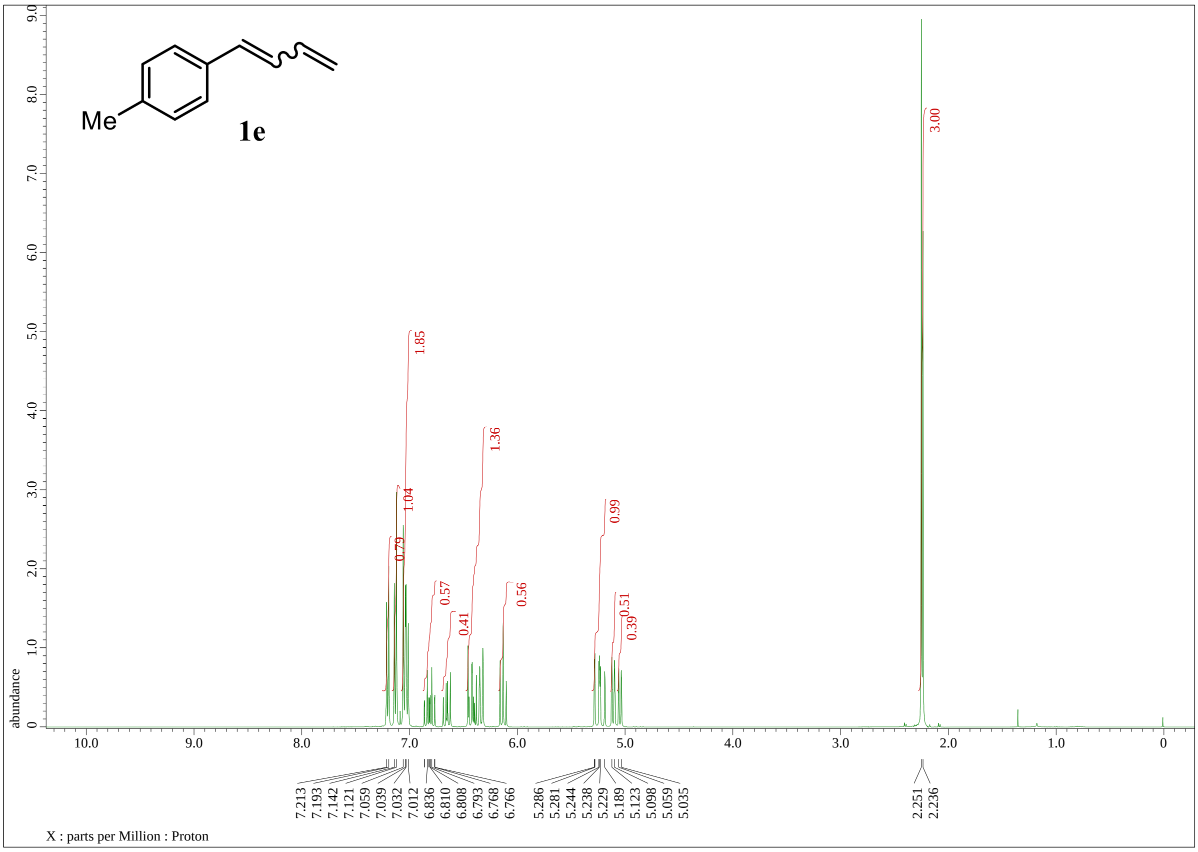
Me
1e
abundance
3.00
1.85
1.36
1.04
0.79
0.57
0.41
0.56
0.99
0.51
0.39
7.213
7.193
7.142
7.121
7.059
7.039
7.032
7.012
6.836
6.810
6.808
6.793
6.768
6.766
5.286
5.281
5.244
5.238
5.229
5.189
5.123
5.098
5.059
5.035
2.251
2.236
X : parts per Million : Proton

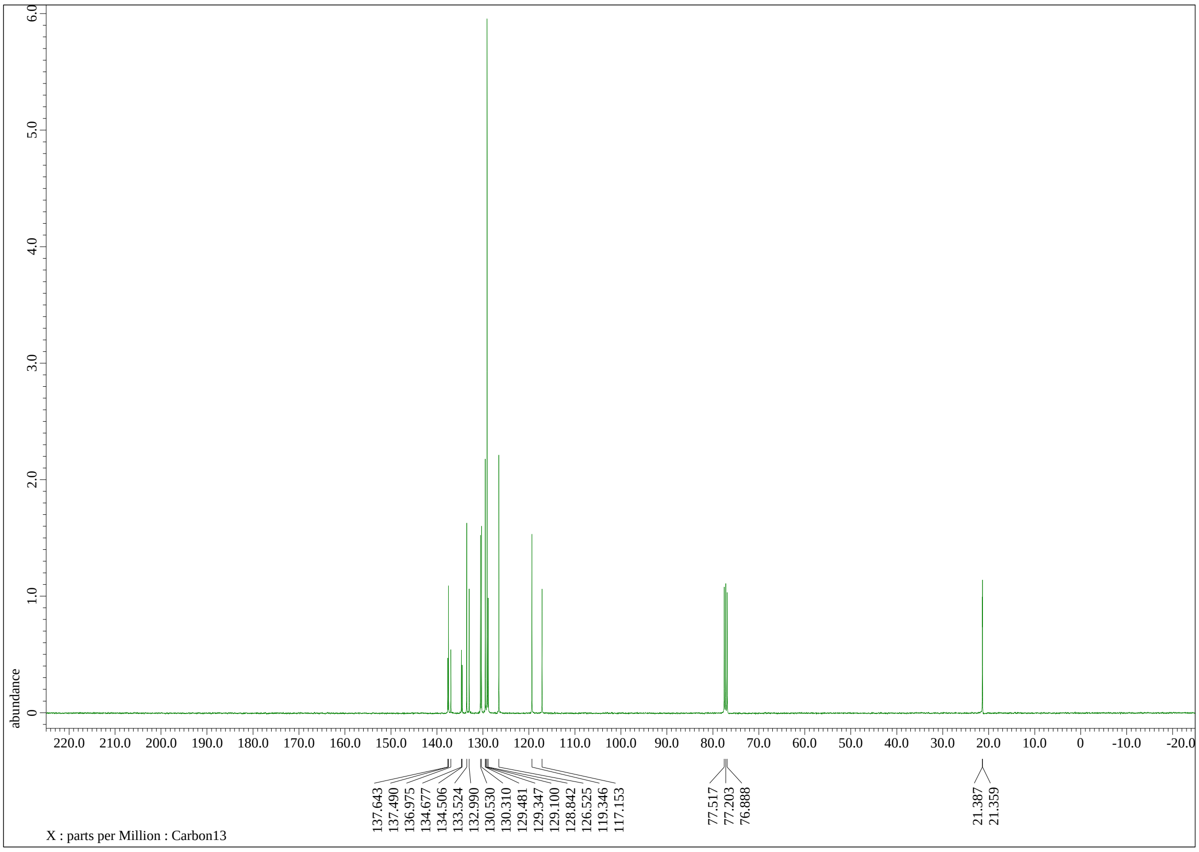
abundance
137.643
137.490
136.975
134.677
134.506
133.524
132.990
130.530
130.310
129.481
129.347
129.100
128.842
126.525
119.346
117.153
77.517
77.203
76.888
21.387
21.359
X : parts per Million : Carbon13

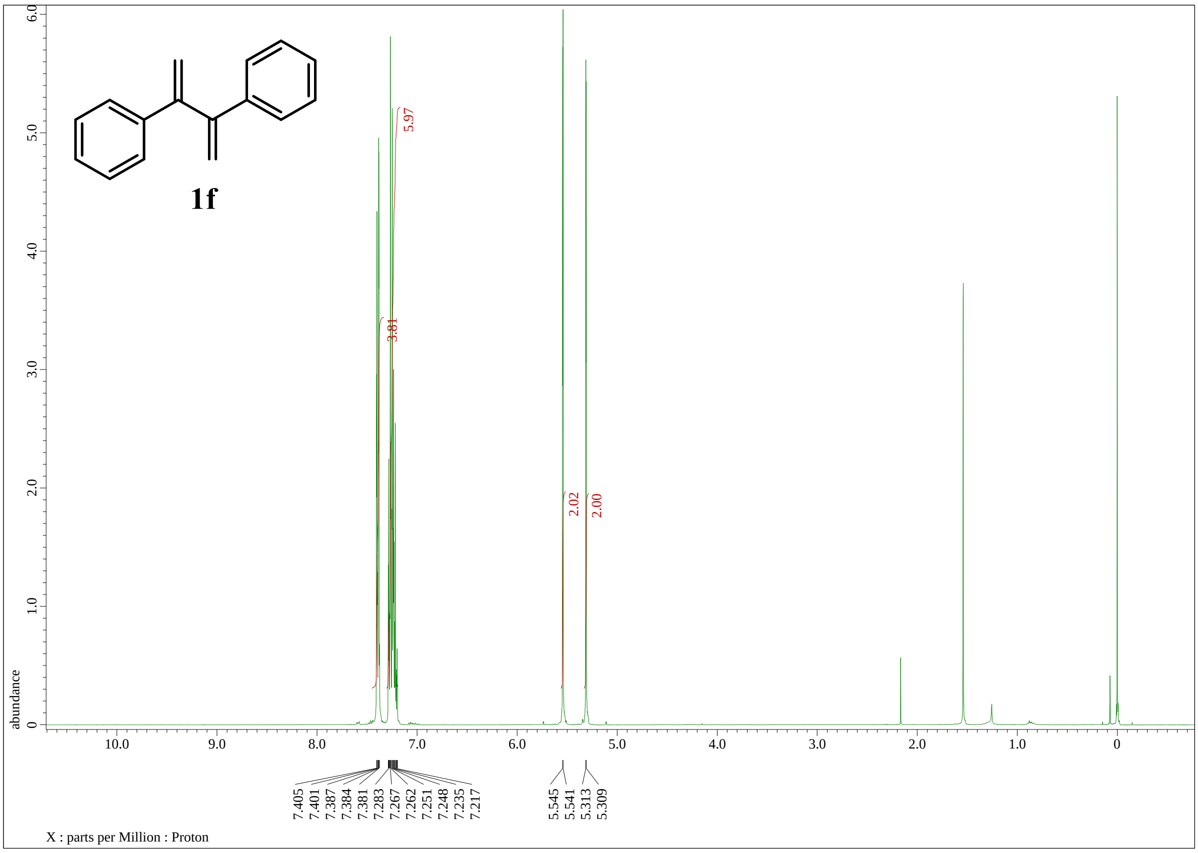
1f
abundance
X : parts per Million : Proton
5.97
3.81
2.02
2.00
7.405
7.401
7.387
7.384
7.381
7.283
7.267
7.262
7.251
7.248
7.235
7.217
5.545
5.541
5.313
5.309

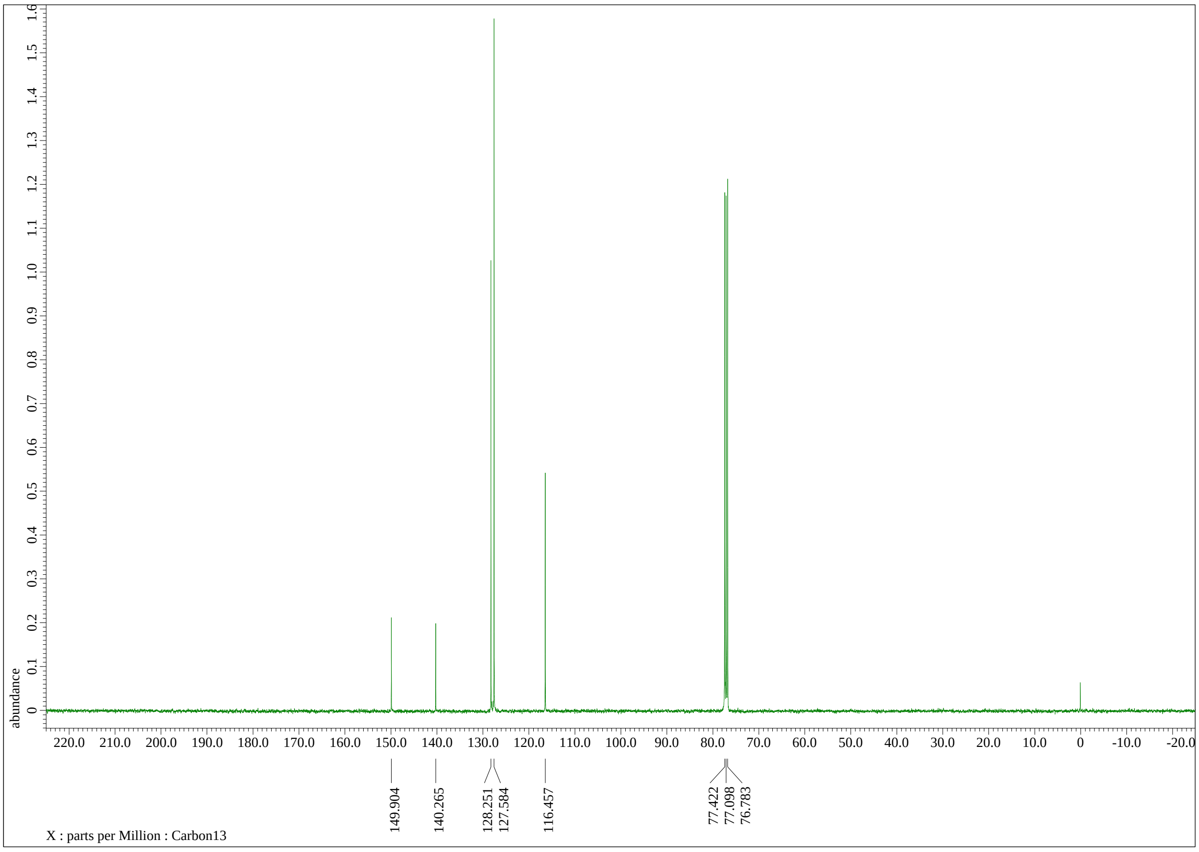
abundance
X : parts per Million : Carbon13
149.904
140.265
128.251
127.584
116.457
77.422
77.098
76.783

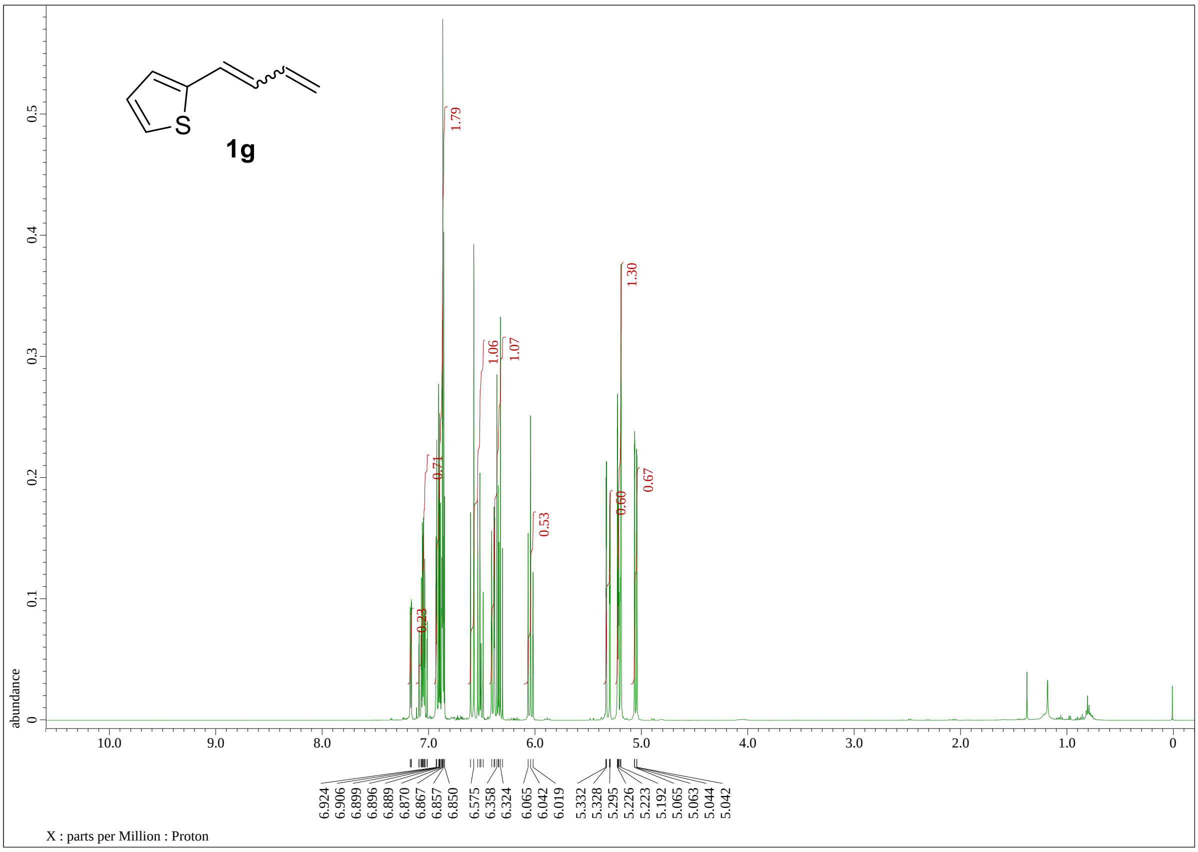

S
1g
abundance
0
0.1
0.2
0.3
0.4
0.5
0.23
0.71
1.79
1.06
1.07
0.53
0.60
1.30
0.67
10.0 9.0 8.0 7.0 6.0 5.0 4.0 3.0 2.0 1.0 0
6.924
6.906
6.899
6.896
6.889
6.870
6.867
6.857
6.850
6.575
6.358
6.324
6.065
6.042
6.019
5.332
5.328
5.295
5.226
5.223
5.192
5.065
5.063
5.044
5.042
X : parts per Million : Proton

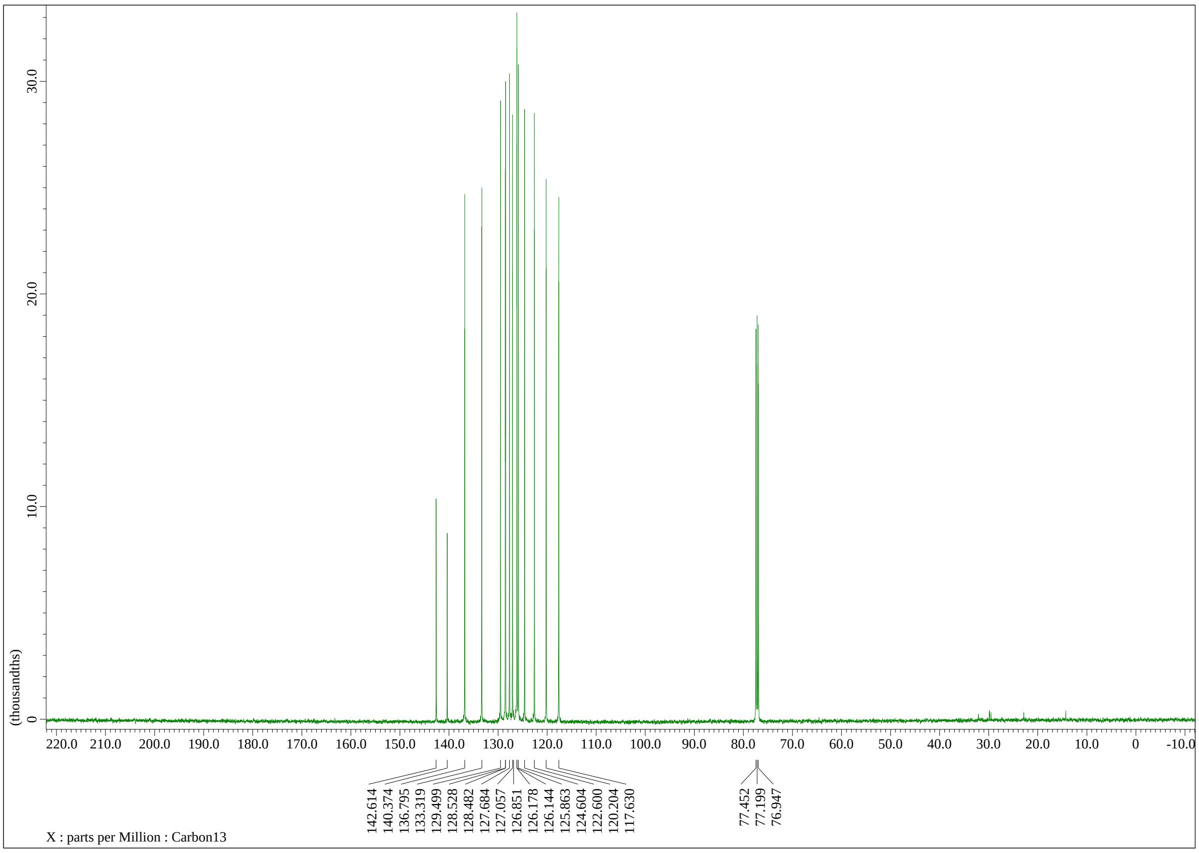

(thousandths)
0
10.0
20.0
30.0
220.0 210.0 200.0 190.0 180.0 170.0 160.0 150.0 140.0 130.0 120.0 110.0 100.0 90.0 80.0 70.0 60.0 50.0 40.0 30.0 20.0 10.0 0 -10.0
142.614
140.374
136.795
133.319
129.499
128.528
128.482
127.684
127.057
126.851
126.178
126.144
125.863
124.604
122.600
120.204
117.630
77.452
77.199
76.947
X : parts per Million : Carbon13

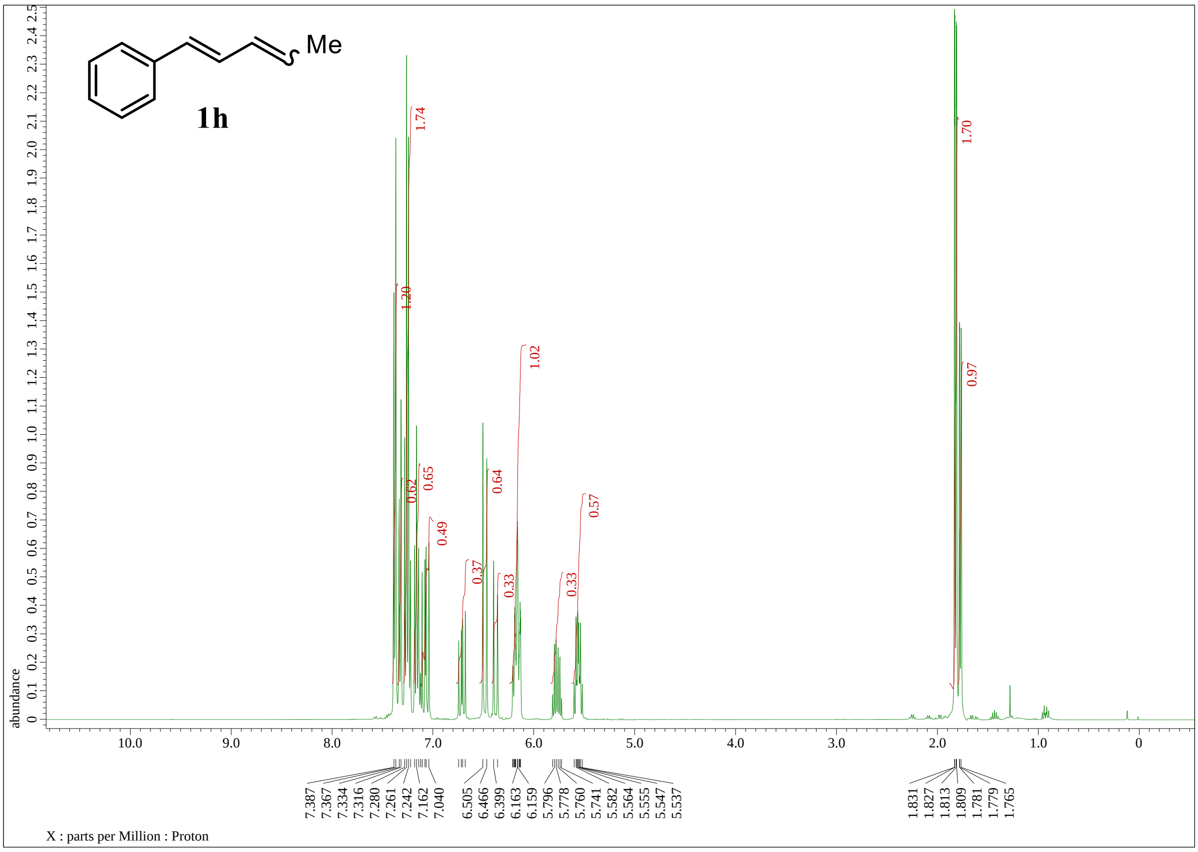
Me
1h
abundance
1.74
1.20
0.62
0.65
0.49
0.37
0.64
0.33
1.02
0.33
0.57
1.70
0.97
7.387
7.367
7.334
7.316
7.280
7.261
7.242
7.162
7.040
6.505
6.466
6.399
6.163
6.159
5.796
5.778
5.760
5.741
5.582
5.564
5.555
5.547
5.537
1.831
1.827
1.813
1.809
1.781
1.779
1.765
X : parts per Million : Proton

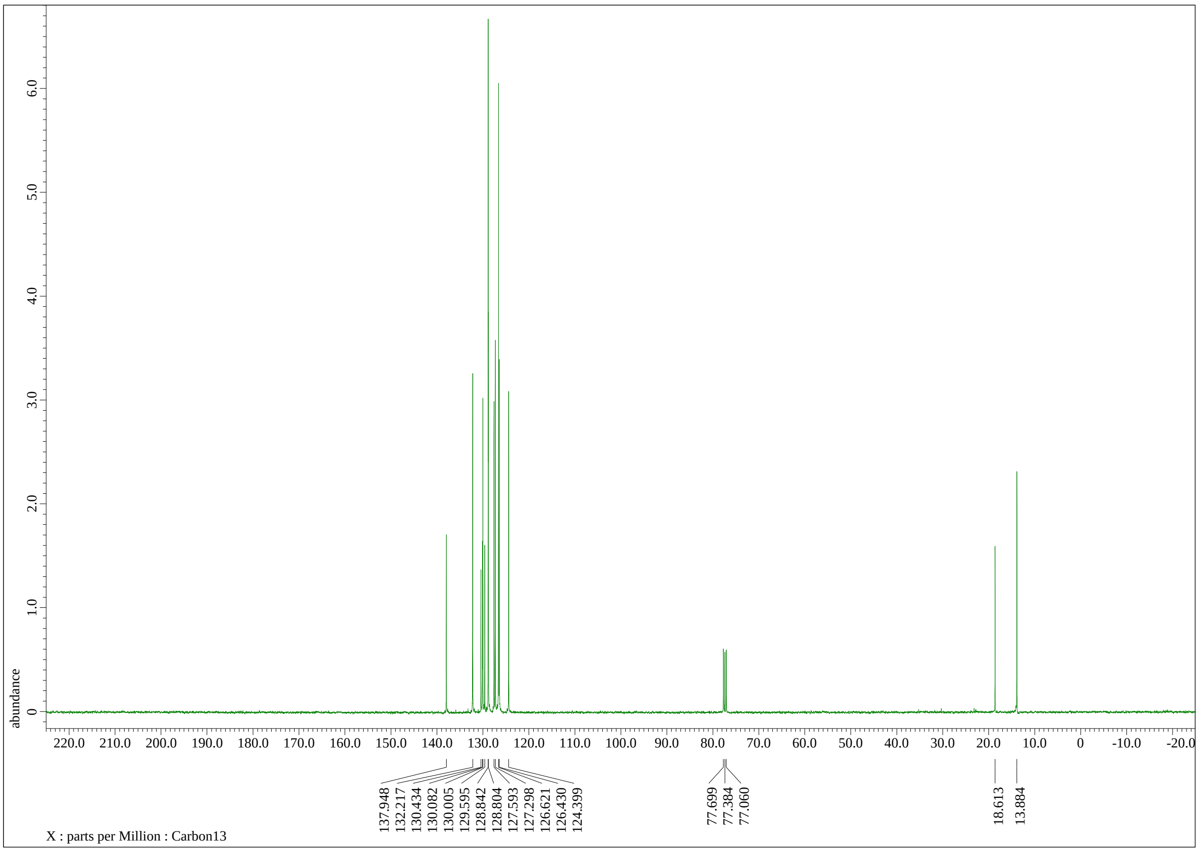
abundance
137.948
132.217
130.434
130.082
130.005
129.595
128.842
128.804
127.593
127.298
126.621
126.430
124.399
77.699
77.384
77.060
18.613
13.884
X : parts per Million : Carbon13

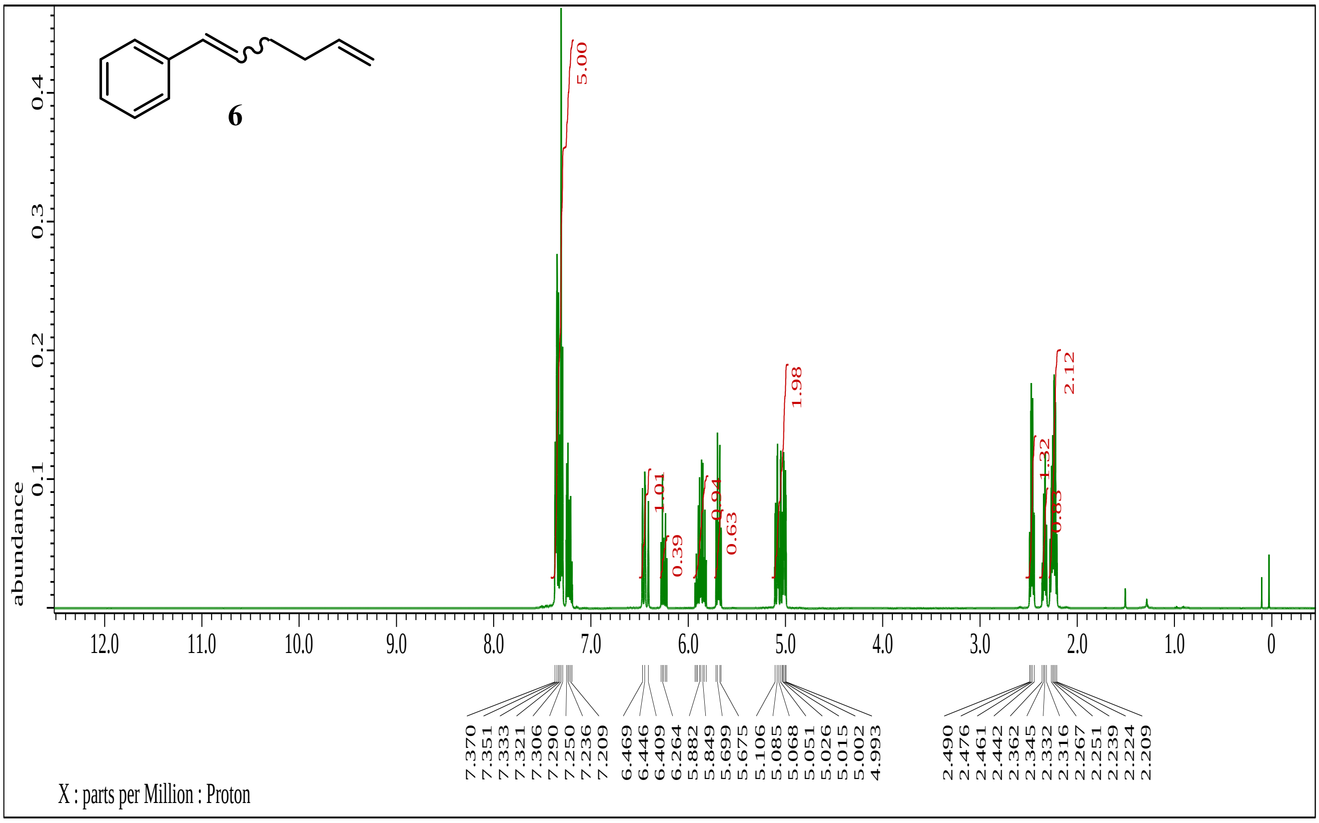
6
abundance
X : parts per Million : Proton
5.00
1.01
0.39
0.94
0.63
1.98
1.32
2.12
7.370 7.351 7.333 7.321 7.306 7.290 7.250 7.236 7.209
6.469 6.446 6.409 6.264 5.882 5.849 5.699 5.675 5.106 5.085 5.068 5.051 5.026 5.015 5.002 4.993
2.490 2.476 2.461 2.442 2.362 2.345 2.332 2.316 2.267 2.251 2.239 2.224 2.209

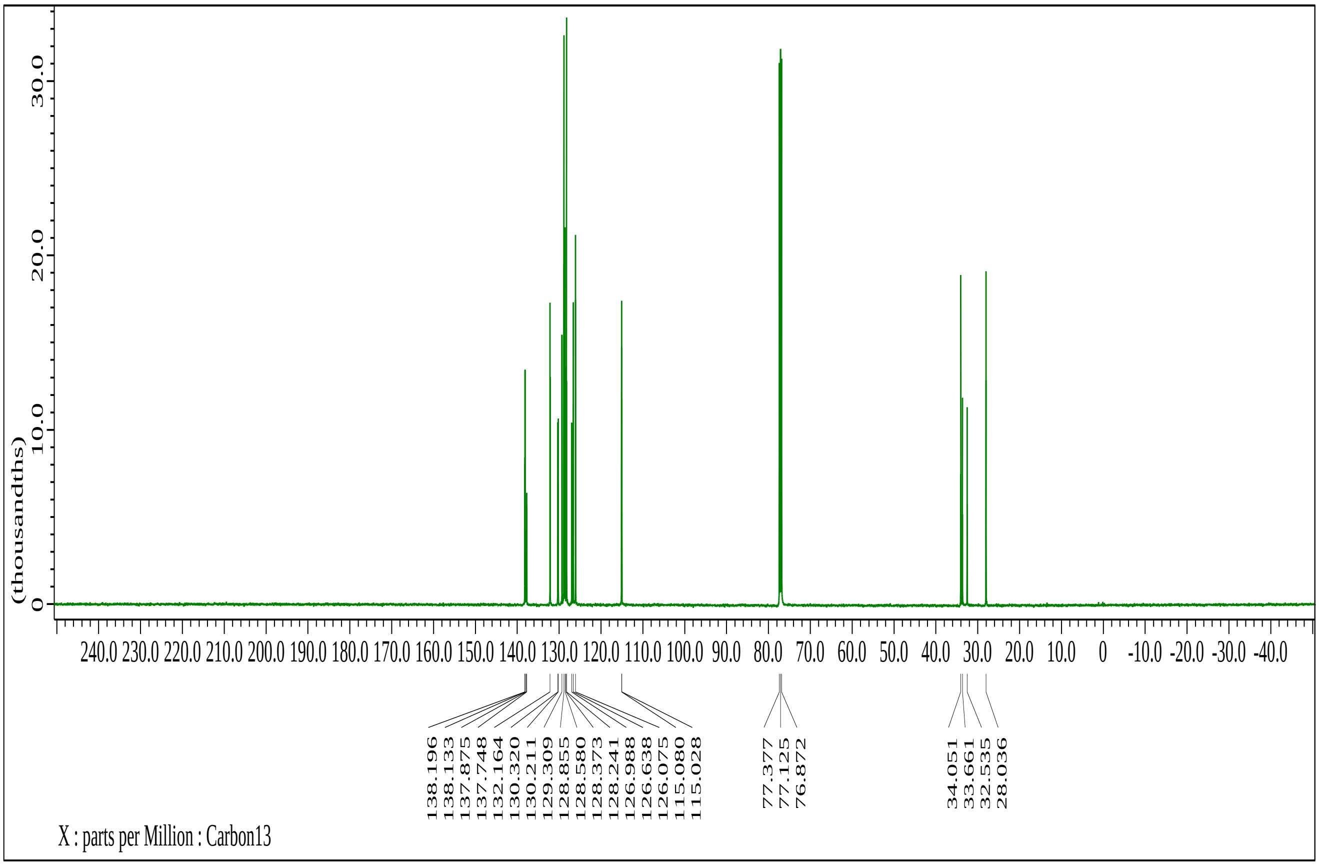
(thousandths)
X : parts per Million : Carbon13
138.196 138.133 137.875 137.748 132.164 130.320 130.211 129.309 128.855 128.580 128.373 128.241 126.988 126.638 126.075 115.080 115.028
77.377 77.125 76.872
34.051 33.661 32.535 28.036

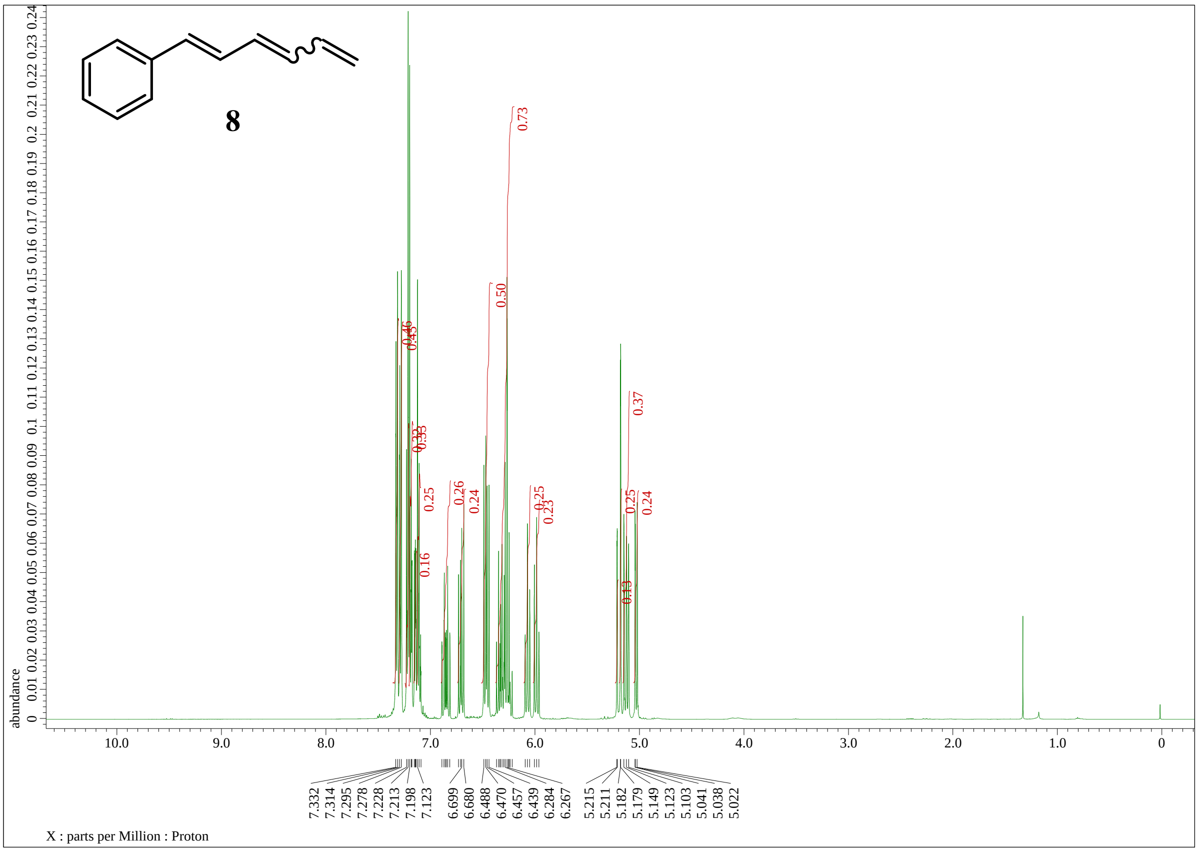
8
abundance
7.332 7.314 7.295 7.278 7.228 7.213 7.198 7.123 6.699 6.680 6.488 6.470 6.457 6.439 6.284 6.267 5.215 5.211 5.182 5.179 5.149 5.123 5.103 5.041 5.038 5.022
X : parts per Million : Proton

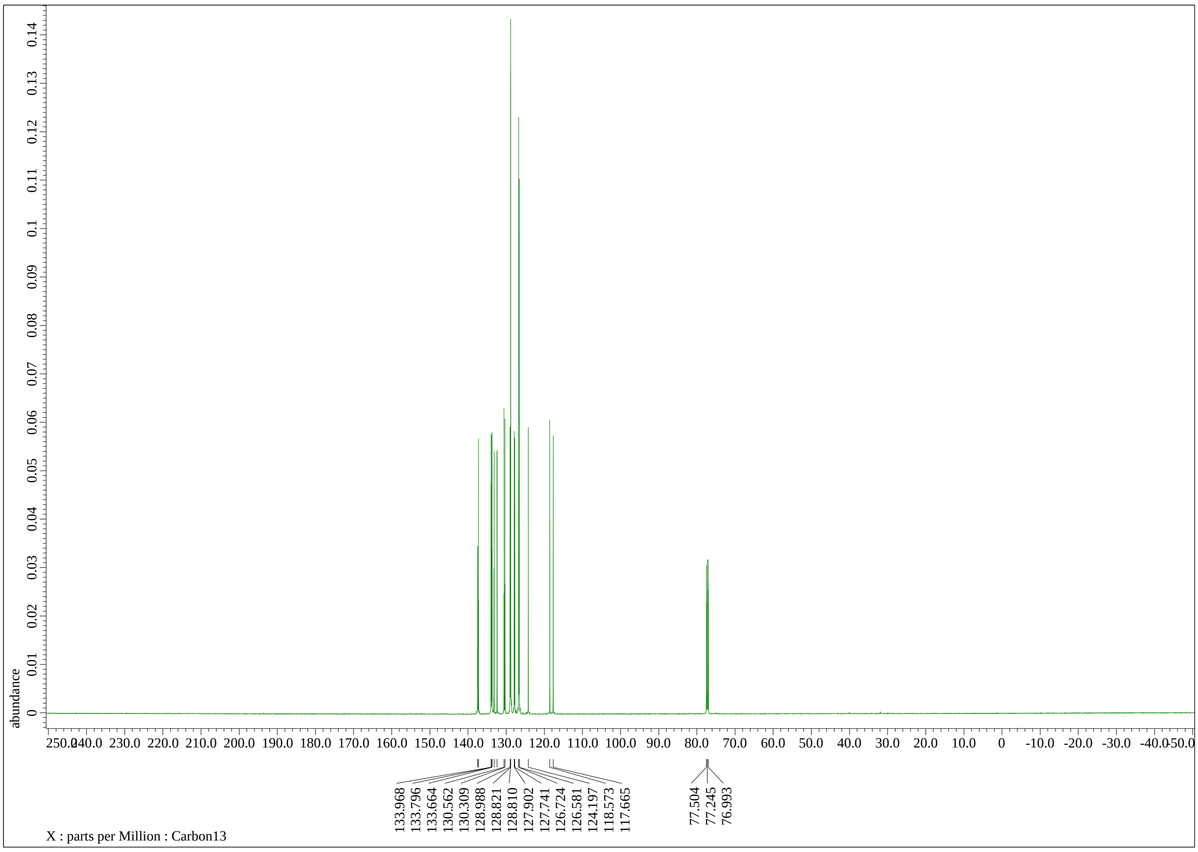
abundance
133.968 133.796 133.664 130.562 130.309 128.988 128.821 128.810 127.902 127.741 126.724 126.581 124.197 118.573 117.665
77.504 77.245 76.993
X : parts per Million : Carbon13

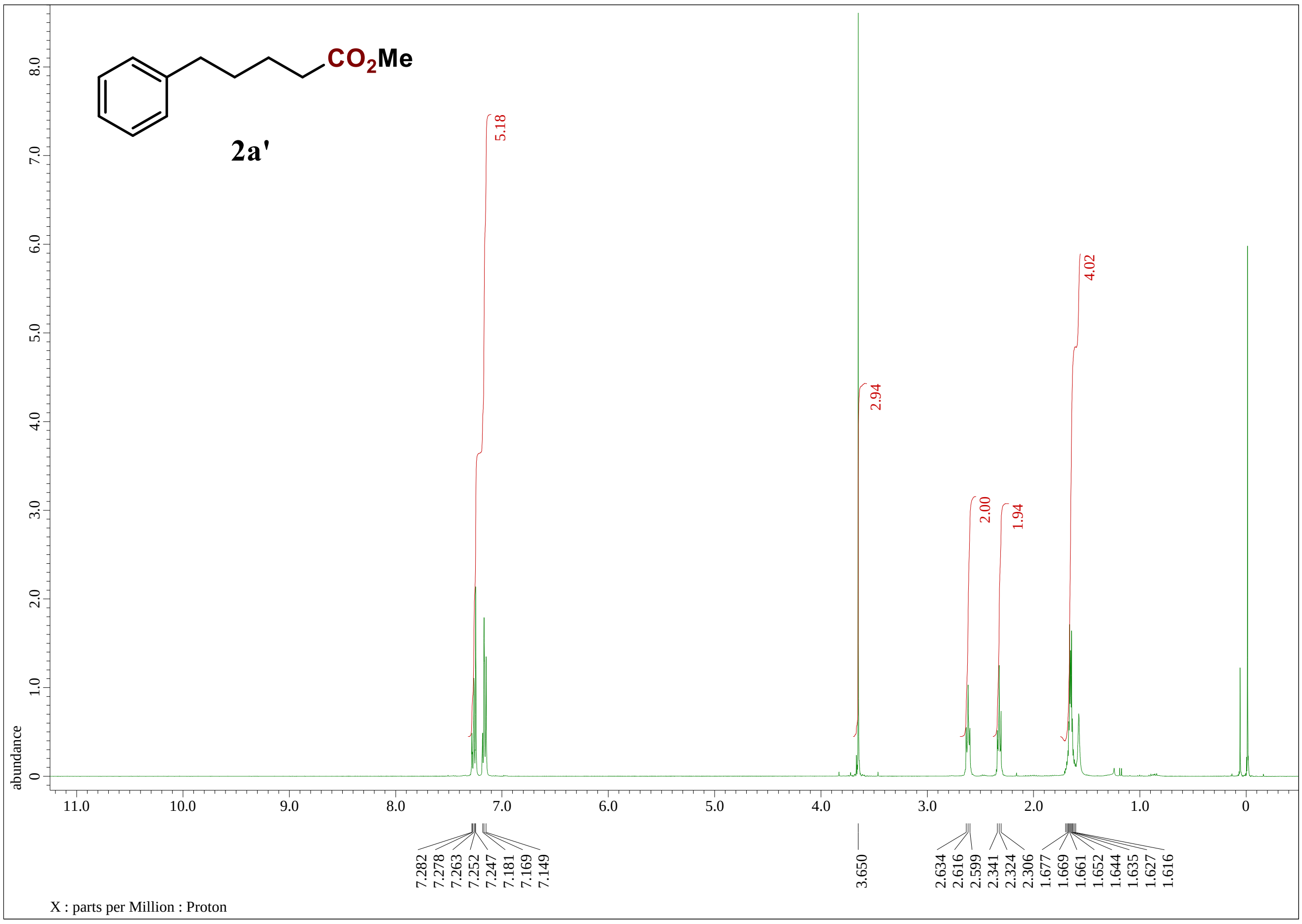
CO2Me
2a'
5.18
2.94
2.00
1.94
4.02
abundance
7.282 7.278 7.263 7.252 7.247 7.181 7.169 7.149
3.650
2.634 2.616 2.599 2.341 2.324 2.306 1.677 1.669 1.661 1.652 1.644 1.635 1.627 1.616
X : parts per Million : Proton

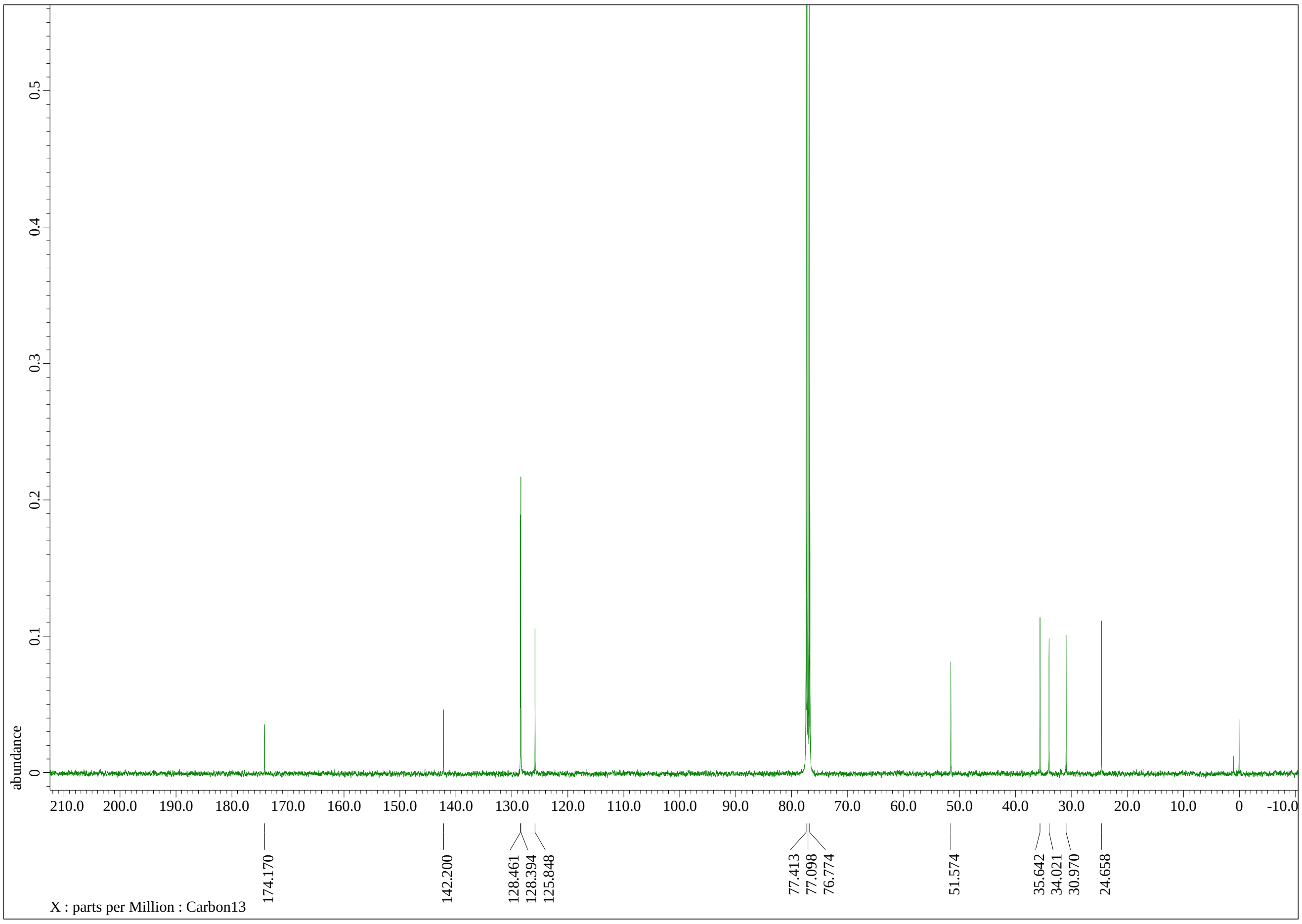
abundance
174.170
142.200
128.461 128.394 125.848
77.413 77.098 76.774
51.574
35.642 34.021 30.970
24.658
X : parts per Million : Carbon13

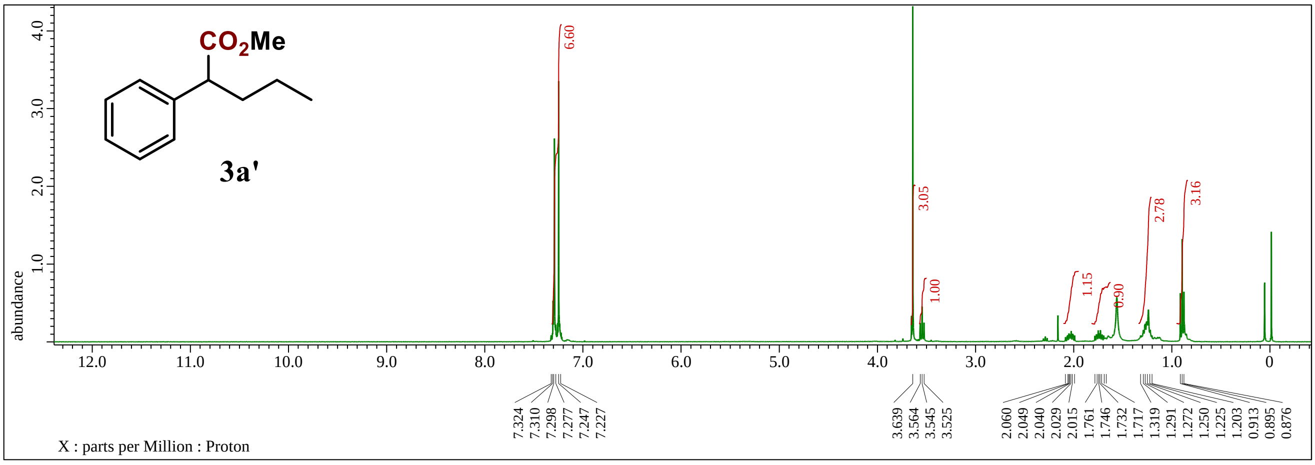

CO2Me
3a'
abundance
X : parts per Million : Proton

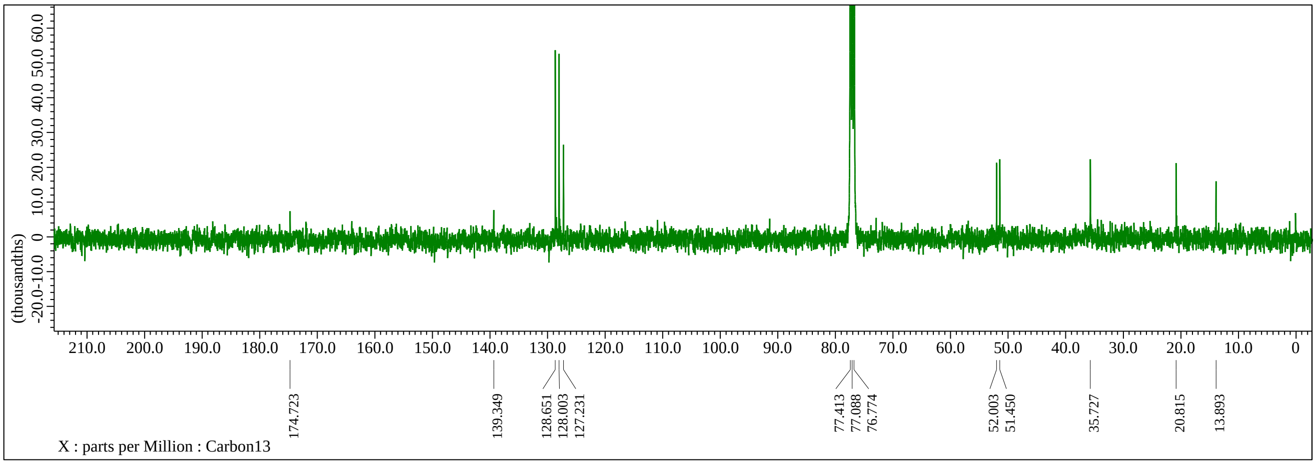

(thousandths)
X : parts per Million : Carbon13

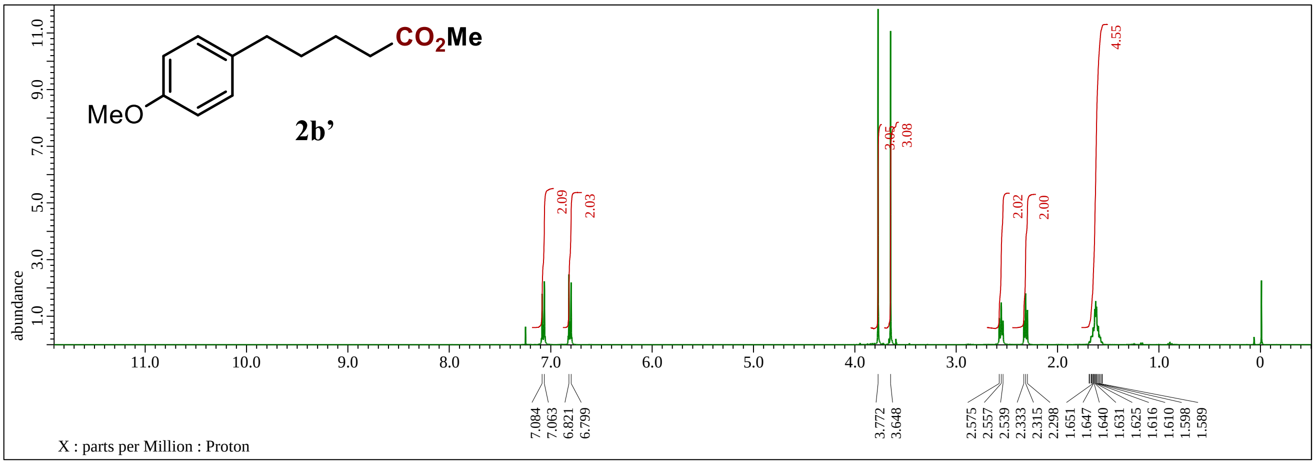
MeO
CO_2Me
2b'
abundance
7.084
7.063
6.821
6.799
3.772
3.648
2.575
2.557
2.539
2.333
2.315
2.298
1.651
1.647
1.640
1.631
1.625
1.616
1.610
1.598
1.589
2.09
2.03
3.05
3.08
2.02
2.00
4.55
X : parts per Million : Proton

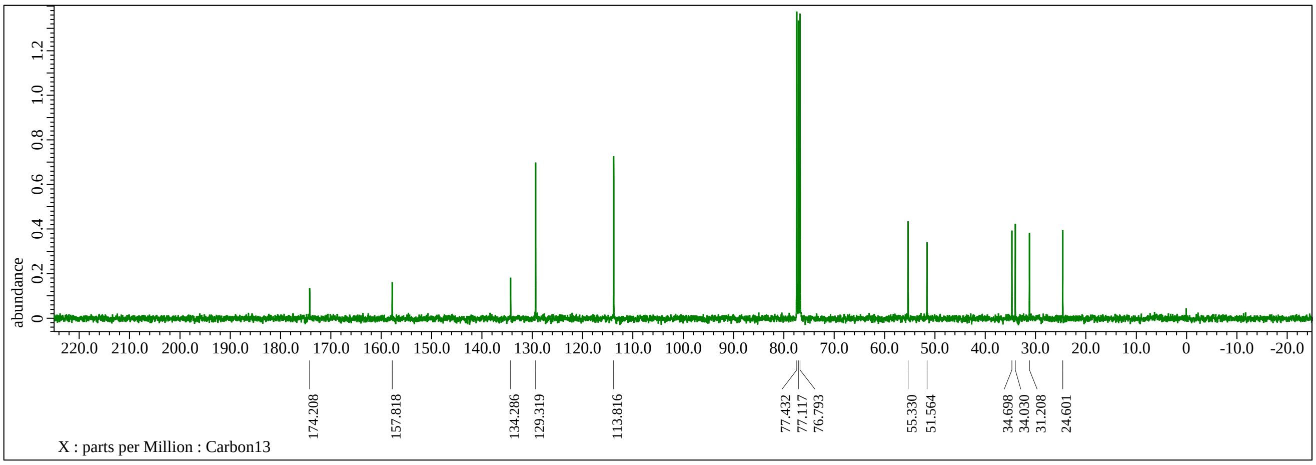
abundance
174.208
157.818
134.286
129.319
113.816
77.432
77.117
76.793
55.330
51.564
34.698
34.030
31.208
24.601
X : parts per Million : Carbon13

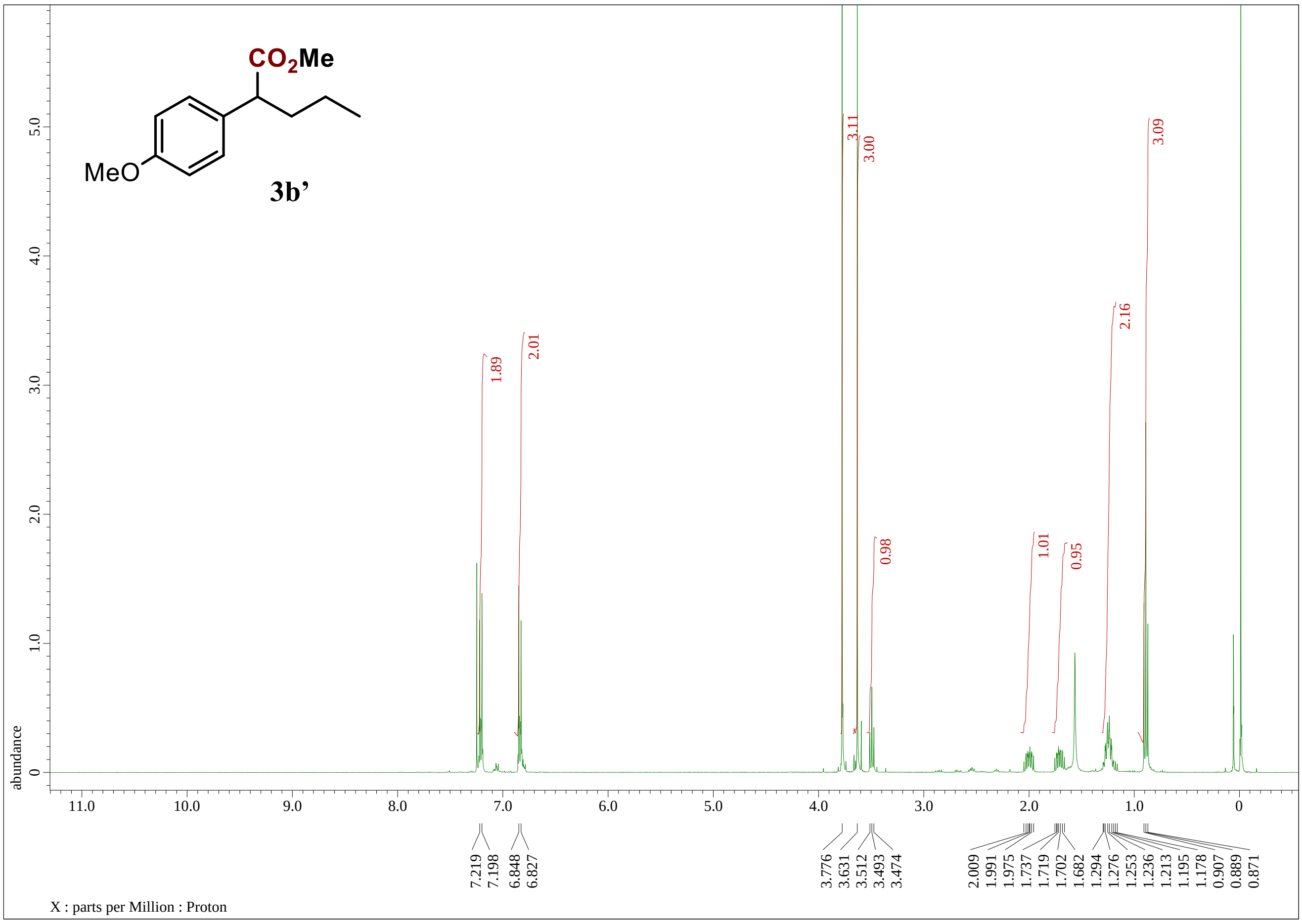
CO2Me
MeO
3b'
abundance
X : parts per Million : Proton
1.89
2.01
3.11
3.00
0.98
1.01
0.95
2.16
3.09
7.219
7.198
6.848
6.827
3.776
3.631
3.512
3.493
3.474
2.009
1.991
1.975
1.737
1.719
1.702
1.682
1.294
1.276
1.253
1.236
1.213
1.195
1.178
0.907
0.889
0.871

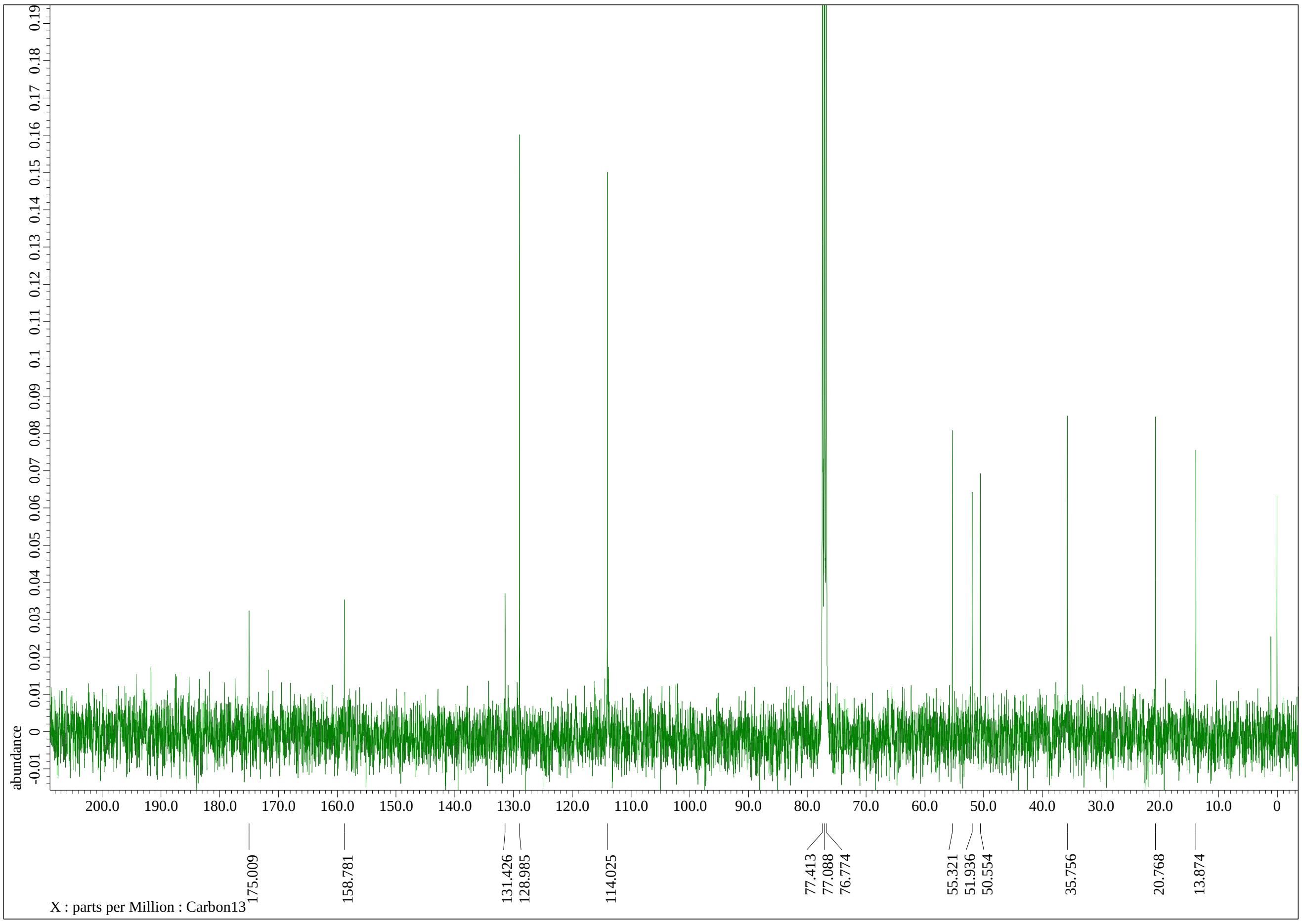
abundance
X : parts per Million : Carbon13
175.009
158.781
131.426
128.985
114.025
77.413
77.088
76.774
55.321
51.936
50.554
35.756
20.768
13.874

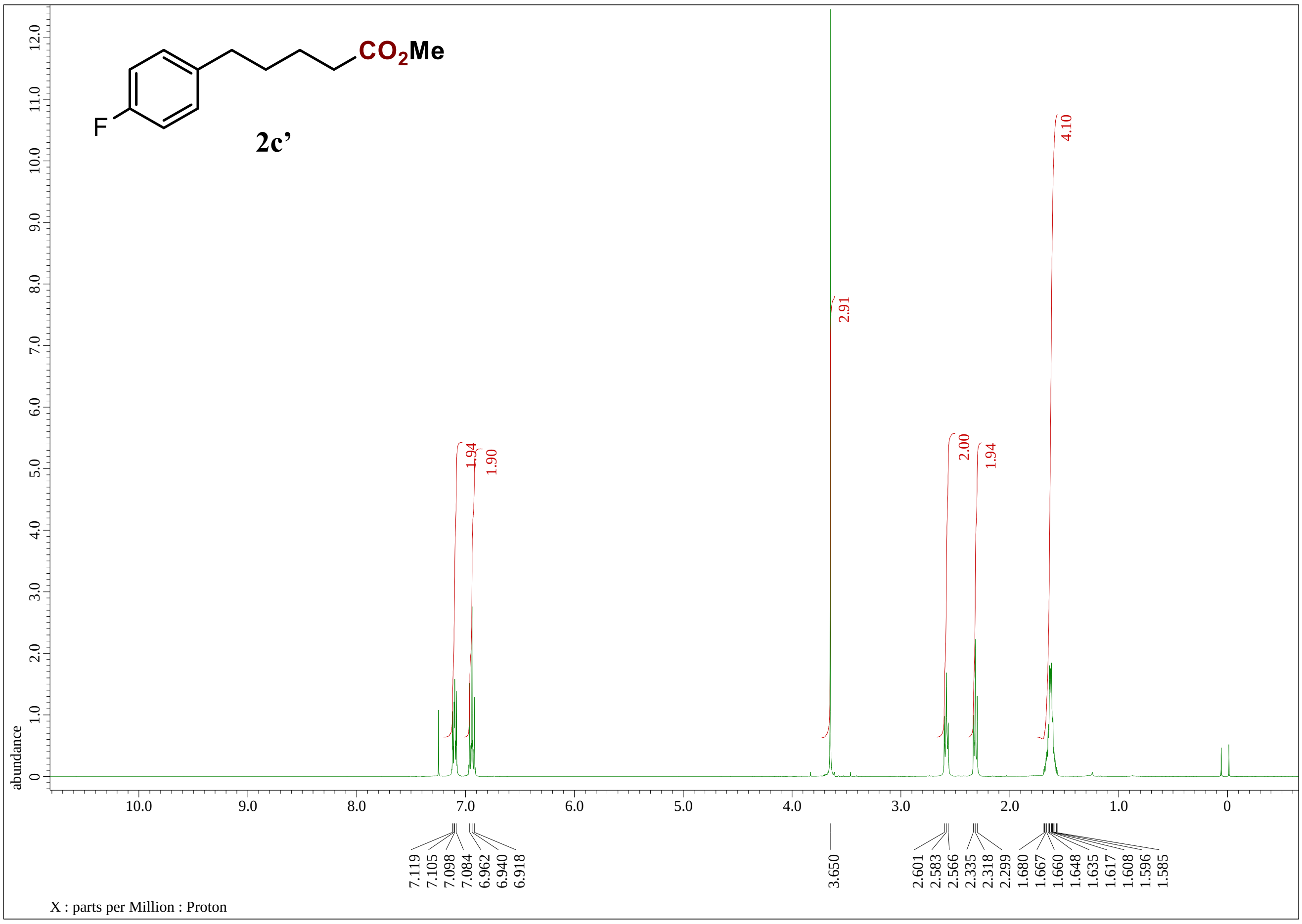

CO2Me
F
2c’
2.91
4.10
1.94
1.90
2.00
1.94
7.119
7.105
7.098
7.084
6.962
6.940
6.918
3.650
2.601
2.583
2.566
2.335
2.318
2.299
1.680
1.667
1.660
1.648
1.635
1.617
1.608
1.596
1.585
abundance
X : parts per Million : Proton

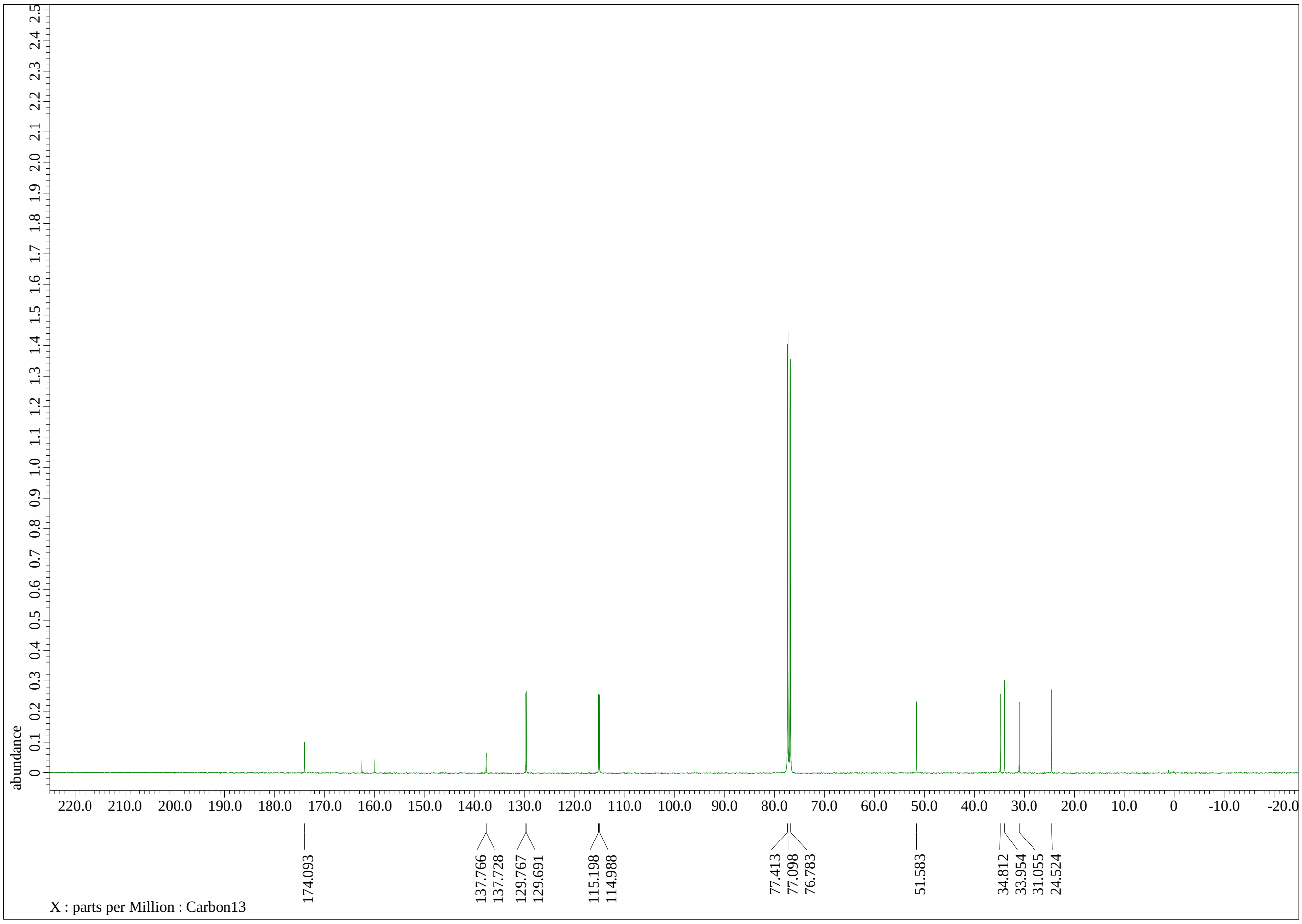

174.093
137.766
137.728
129.767
129.691
115.198
114.988
77.413
77.098
76.783
51.583
34.812
33.954
31.055
24.524
abundance
X : parts per Million : Carbon13

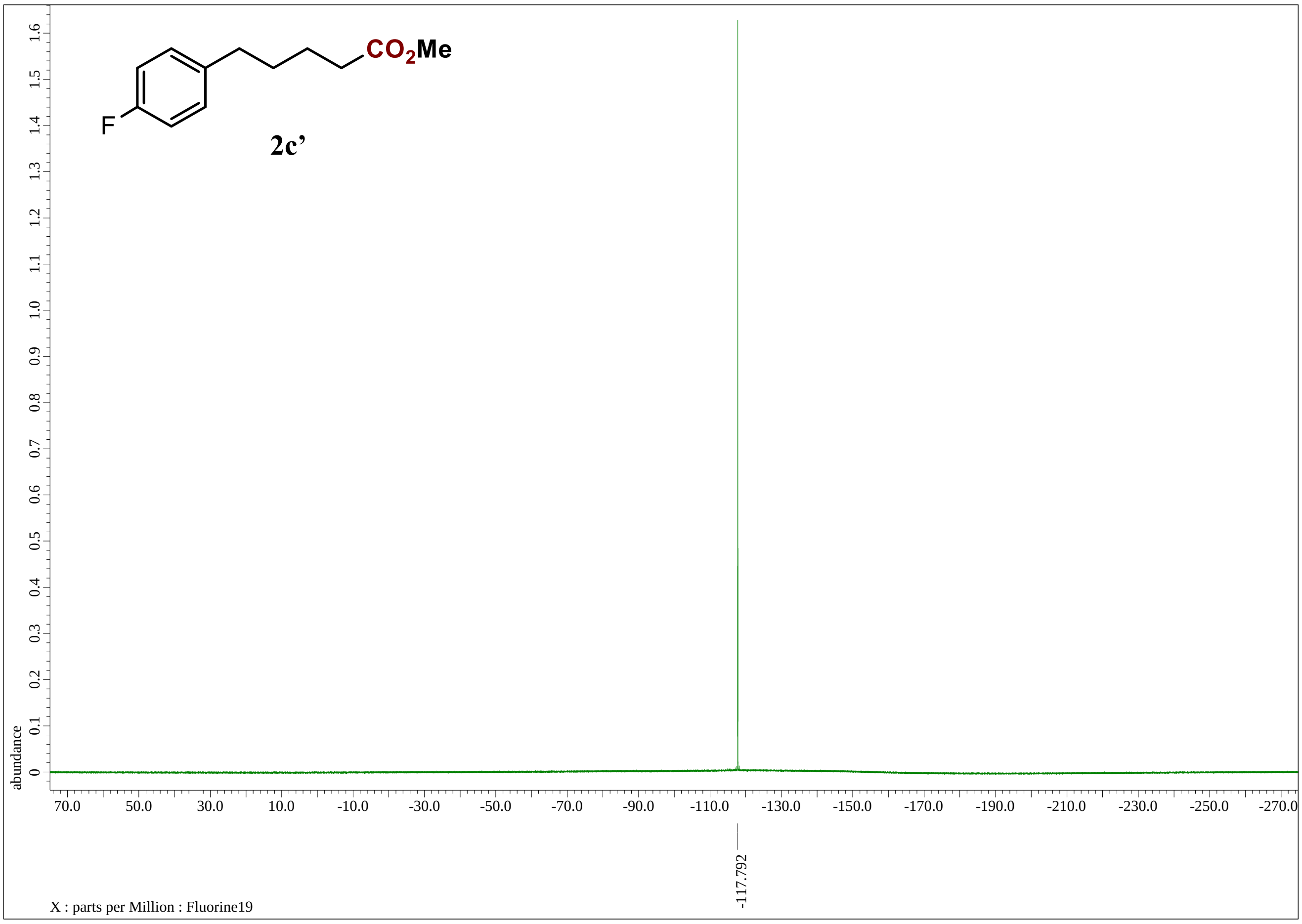
CO2Me
F
2c'
abundance
-117.792
X : parts per Million : Fluorine19

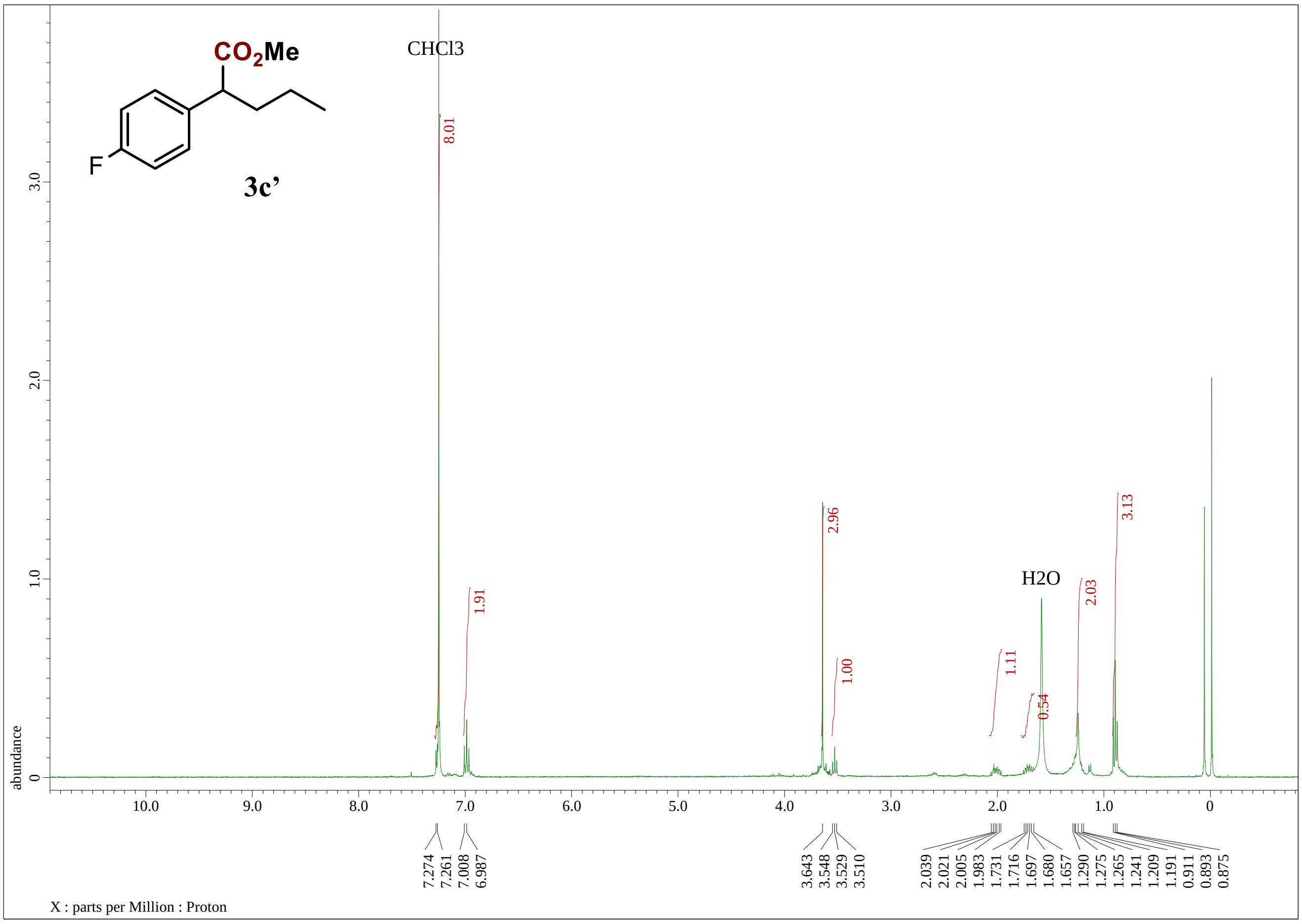
CO2Me
F
3c'
CHCl3
H2O
8.01
1.91
2.96
1.00
1.11
0.54
2.03
3.13
abundance
7.274
7.261
7.008
6.987
3.643
3.548
3.529
3.510
2.039
2.021
2.005
1.983
1.731
1.716
1.697
1.680
1.657
1.290
1.275
1.265
1.241
1.209
1.191
0.911
0.893
0.875
X : parts per Million : Proton

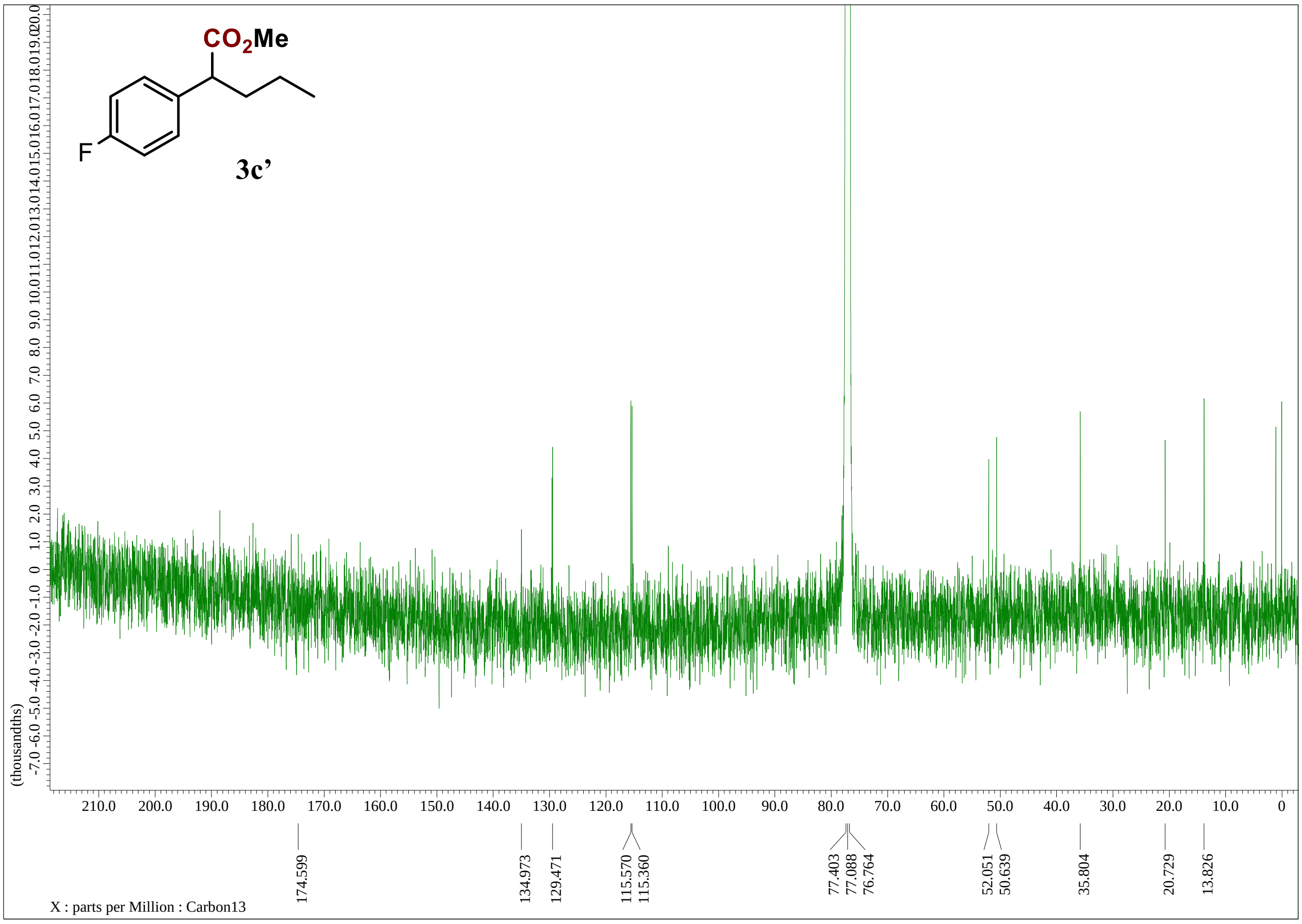
CO2Me
F
3c'
(thousandths)
174.599
134.973
129.471
115.570
115.360
77.403
77.088
76.764
52.051
50.639
35.804
20.729
13.826
X : parts per Million : Carbon13

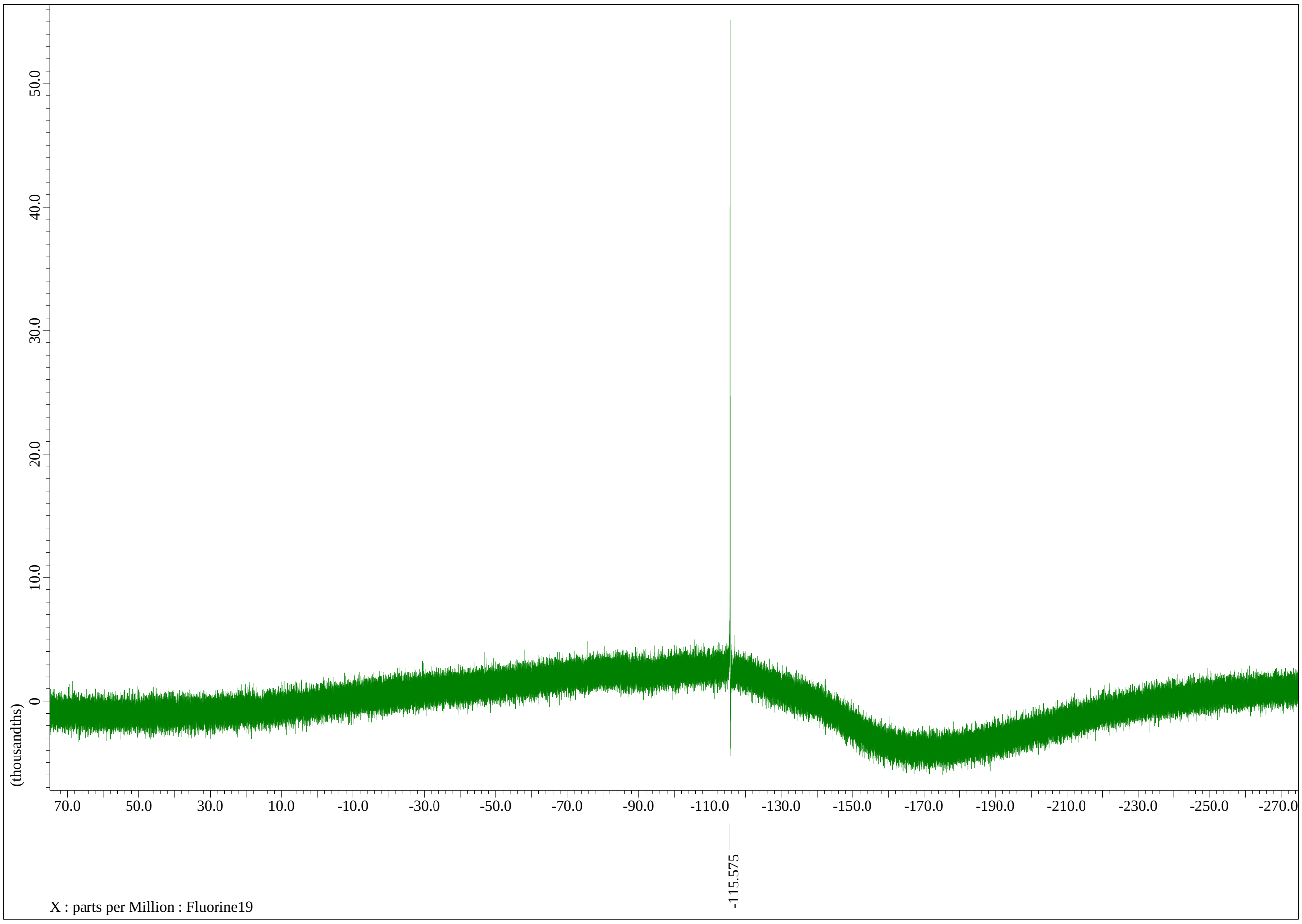
(thousandths)
-115.575
X : parts per Million : Fluorine19

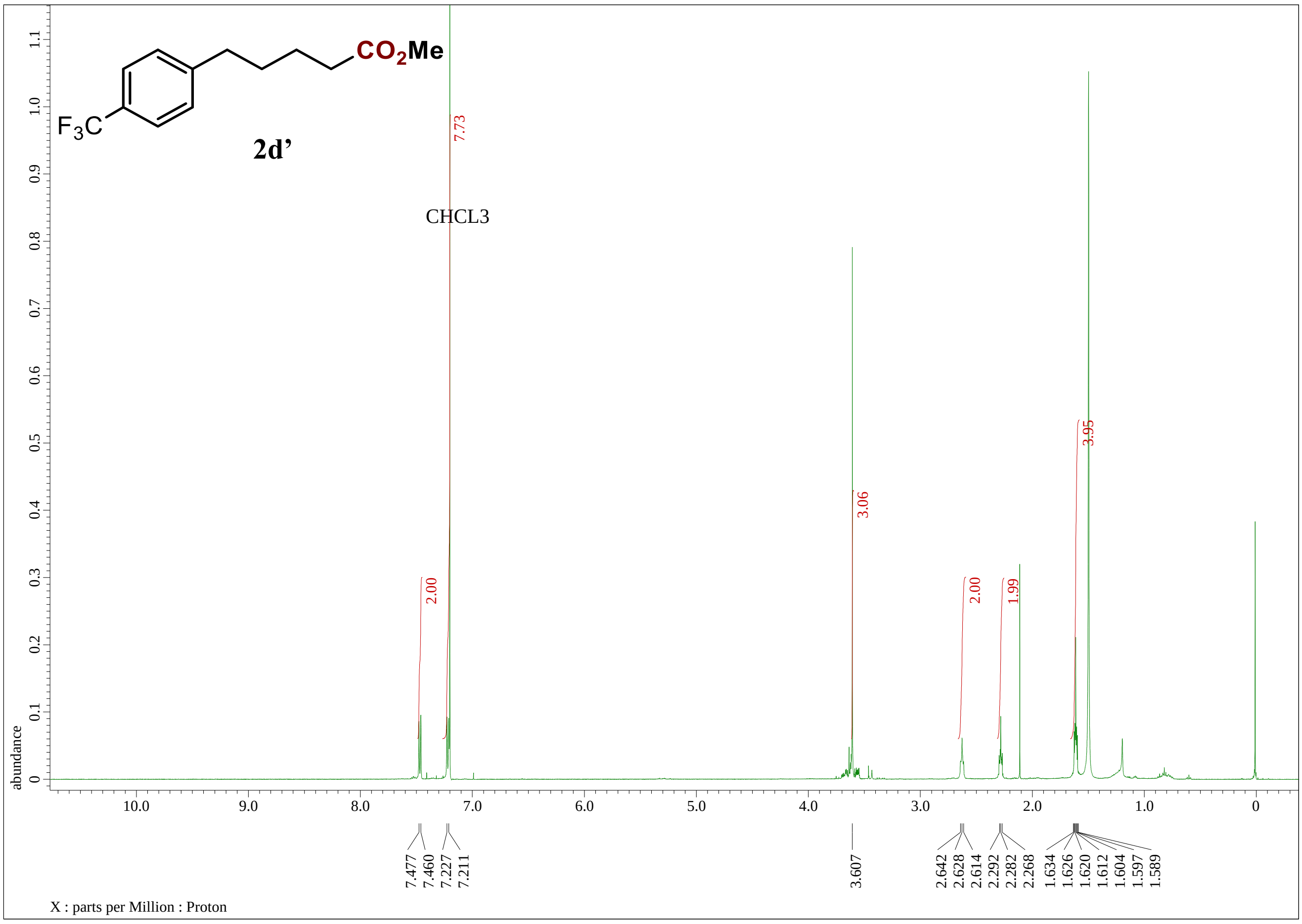
CO2Me
F3C
2d'
CHCL3
7.73
2.00
3.06
2.00
1.99
3.95
abundance
10.0 9.0 8.0 7.0 6.0 5.0 4.0 3.0 2.0 1.0 0
7.477 7.460 7.227 7.211
3.607
2.642 2.628 2.614 2.292 2.282 2.268 1.634 1.626 1.620 1.612 1.604 1.597 1.589
X : parts per Million : Proton

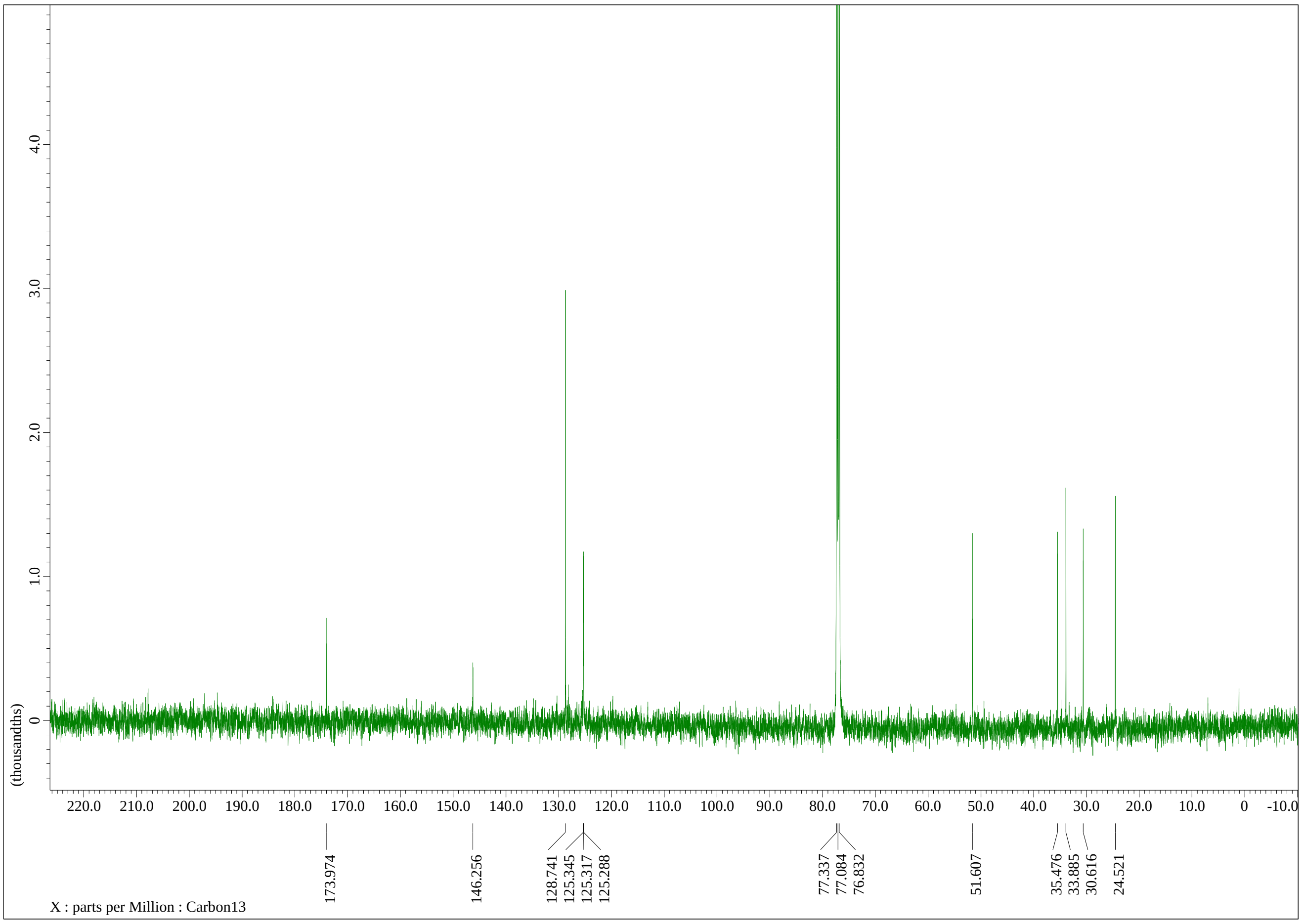
(thousandths)
220.0 210.0 200.0 190.0 180.0 170.0 160.0 150.0 140.0 130.0 120.0 110.0 100.0 90.0 80.0 70.0 60.0 50.0 40.0 30.0 20.0 10.0 0 -10.0
173.974
146.256
128.741 125.345 125.317 125.288
77.337 77.084 76.832
51.607
35.476 33.885 30.616
24.521
X : parts per Million : Carbon13

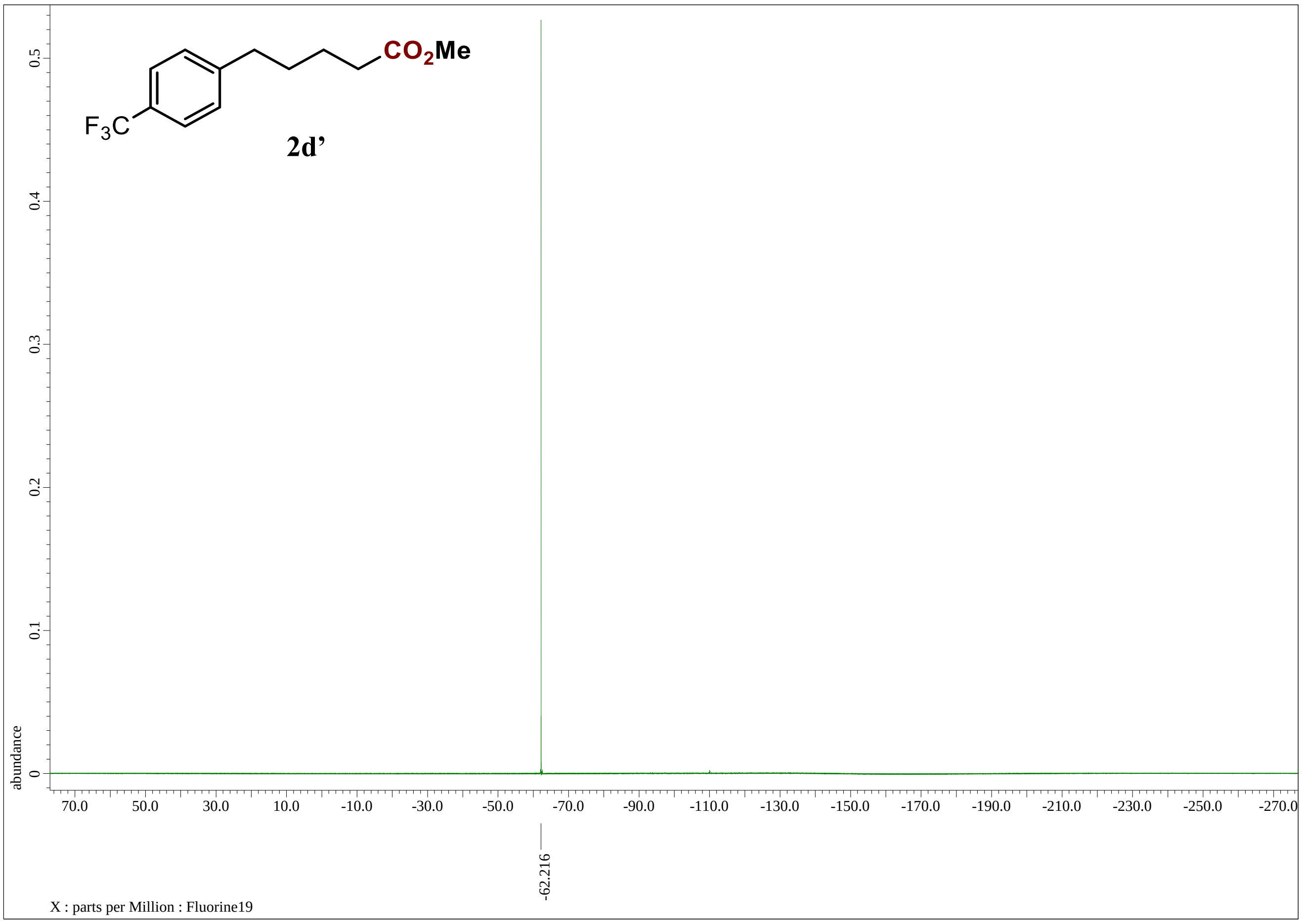

CO2Me
F3C
2d'
abundance
X : parts per Million : Fluorine19
-62.216

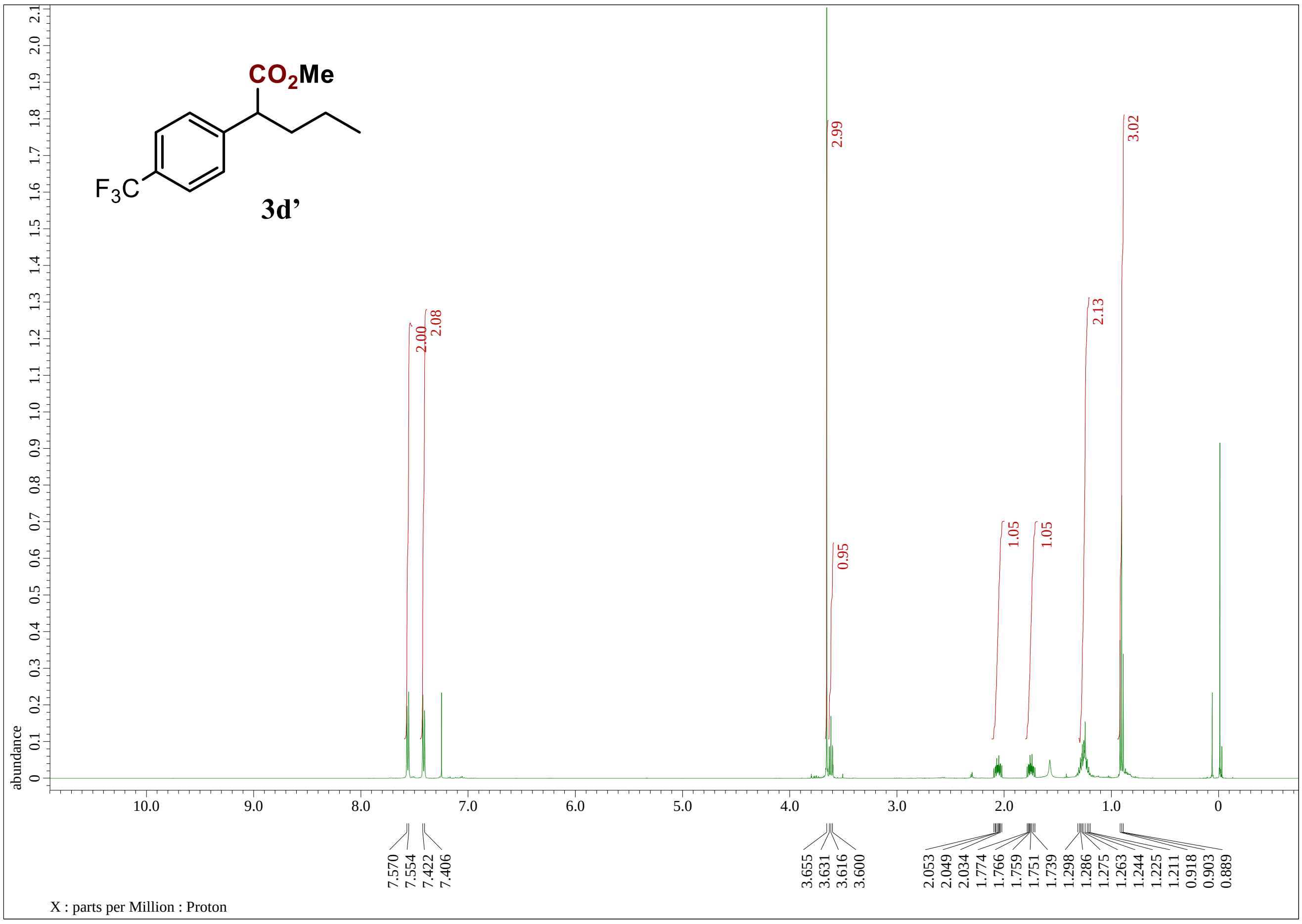

CO2Me
F3C
3d'
abundance
X : parts per Million : Proton
2.00
2.08
2.99
0.95
1.05
1.05
2.13
3.02
7.570
7.554
7.422
7.406
3.655
3.631
3.616
3.600
2.053
2.049
2.034
1.774
1.766
1.759
1.751
1.739
1.298
1.286
1.275
1.263
1.244
1.225
1.211
0.918
0.903
0.889

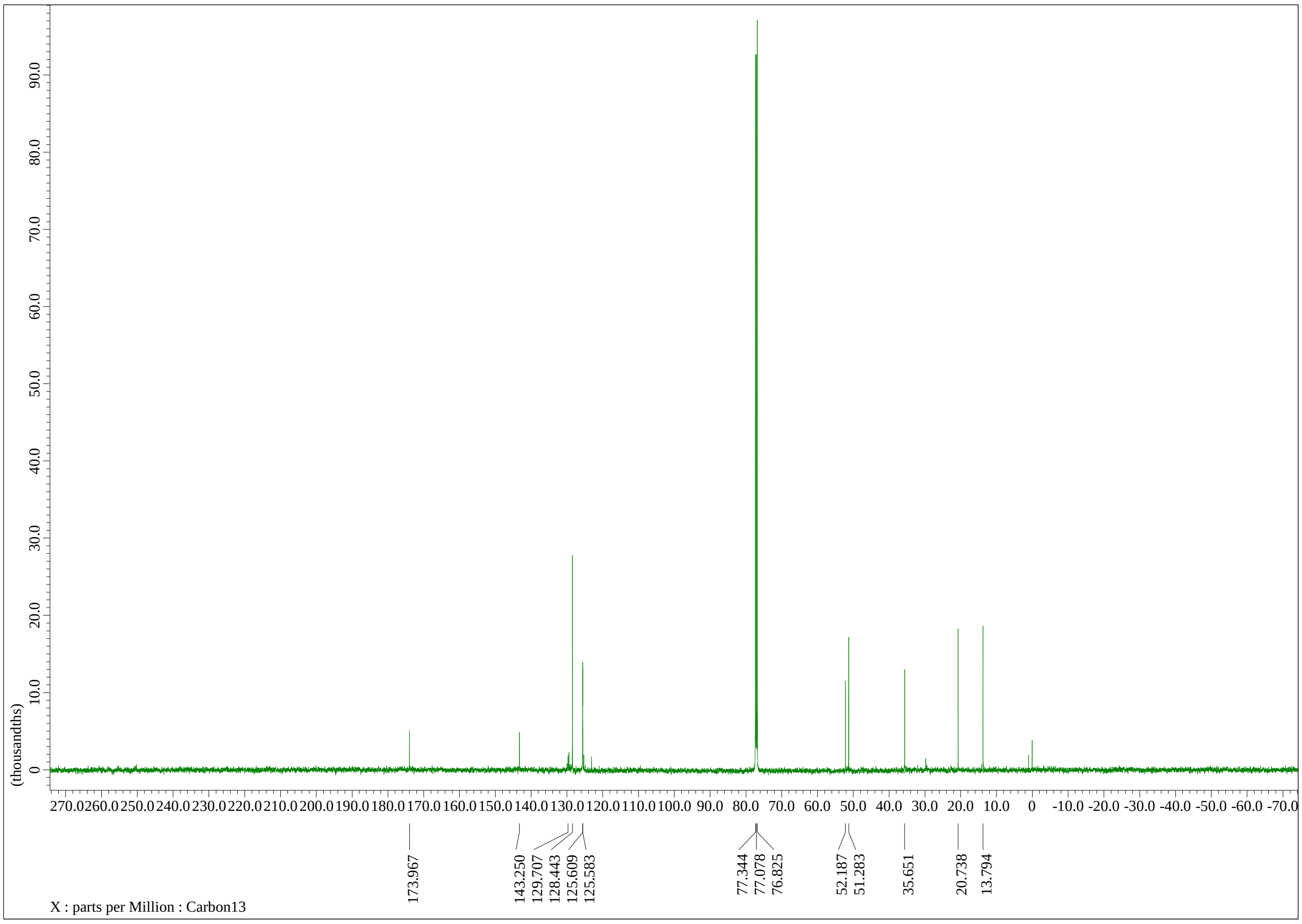
173.967
143.250
129.707
128.443
125.609
125.583
77.344
77.078
76.825
52.187
51.283
35.651
20.738
13.794
(thousandths)
X : parts per Million : Carbon13

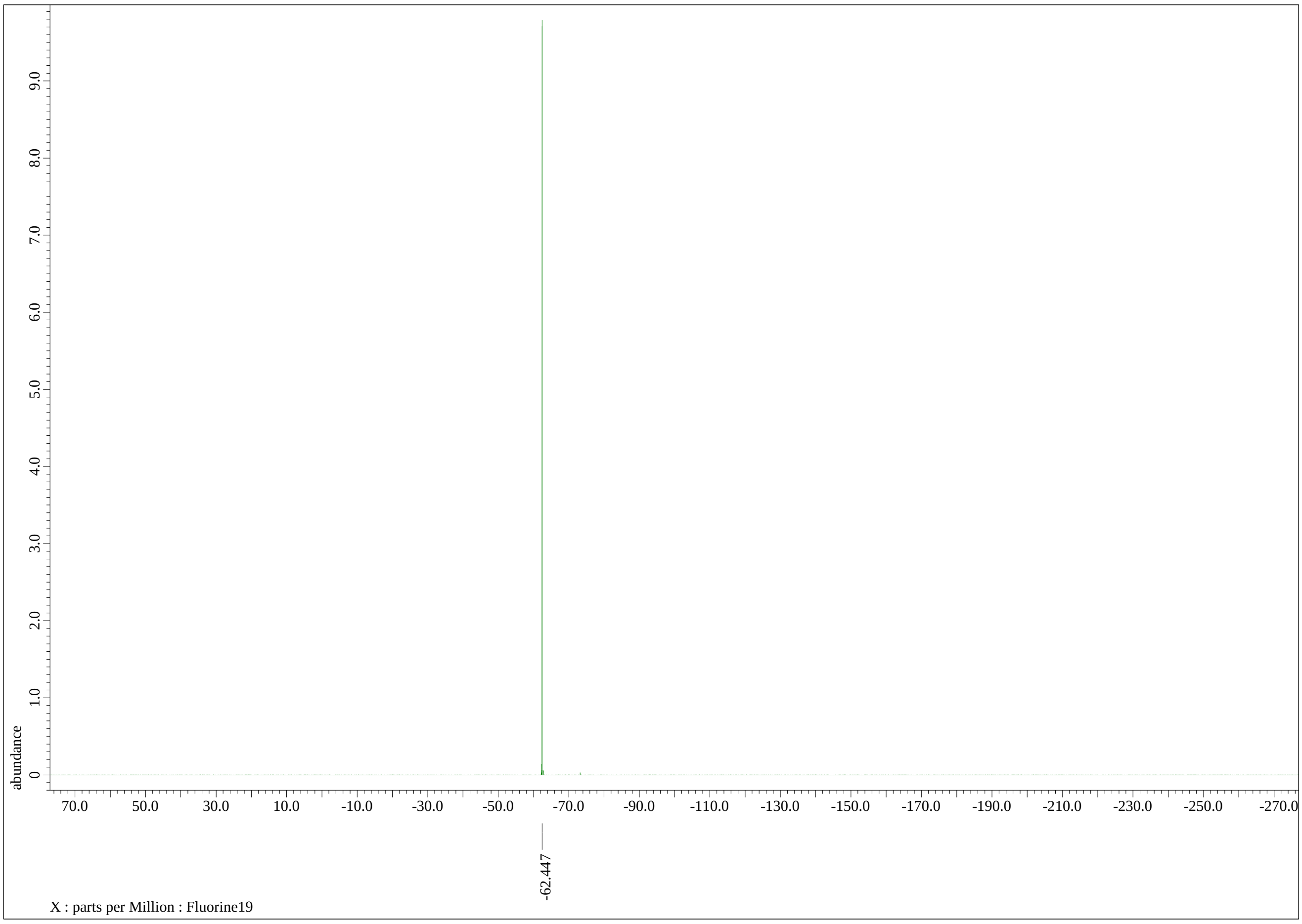
-62.447
abundance
X : parts per Million : Fluorine19

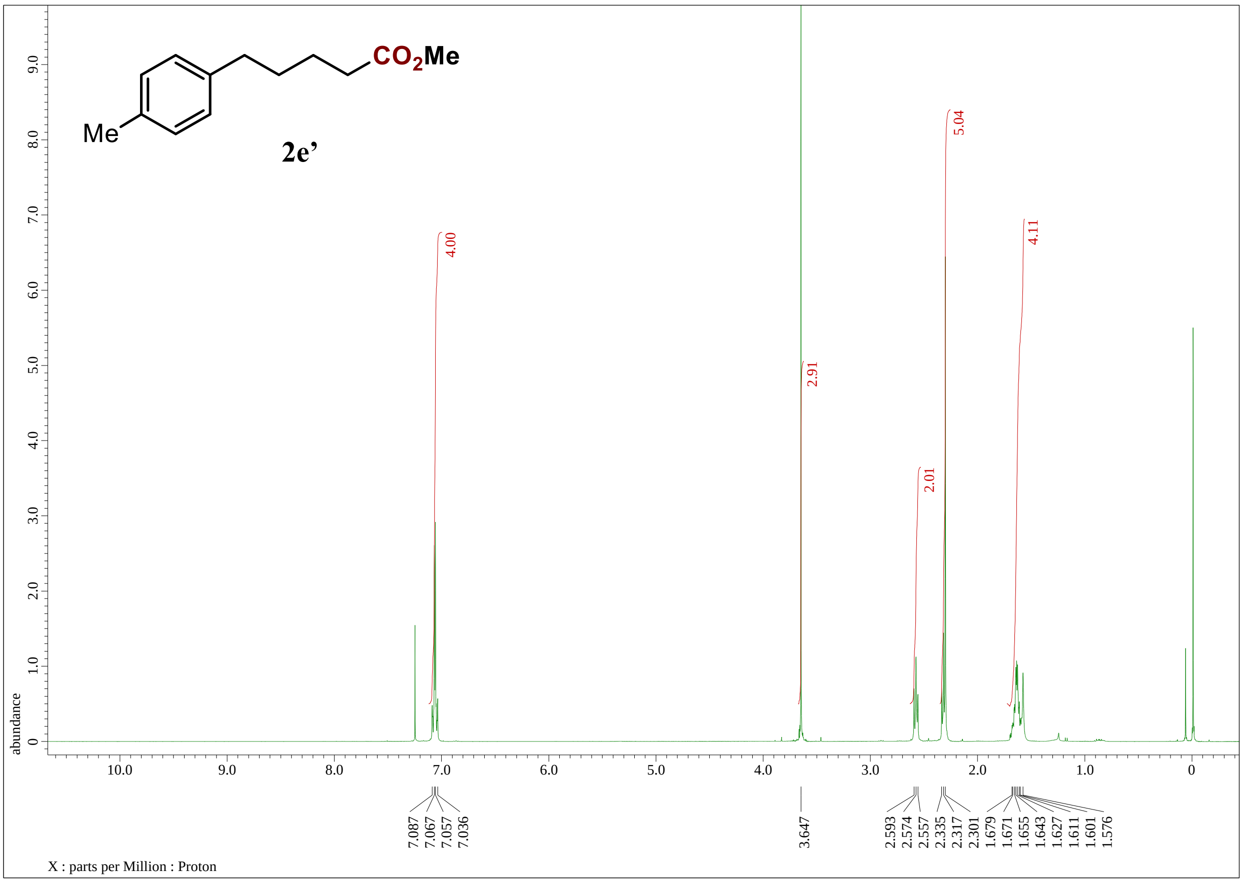
CO2Me
Me
2e'
4.00
2.91
2.01
5.04
4.11
abundance
7.087
7.067
7.057
7.036
3.647
2.593
2.574
2.557
2.335
2.317
2.301
1.679
1.671
1.655
1.643
1.627
1.611
1.601
1.576
X : parts per Million : Proton

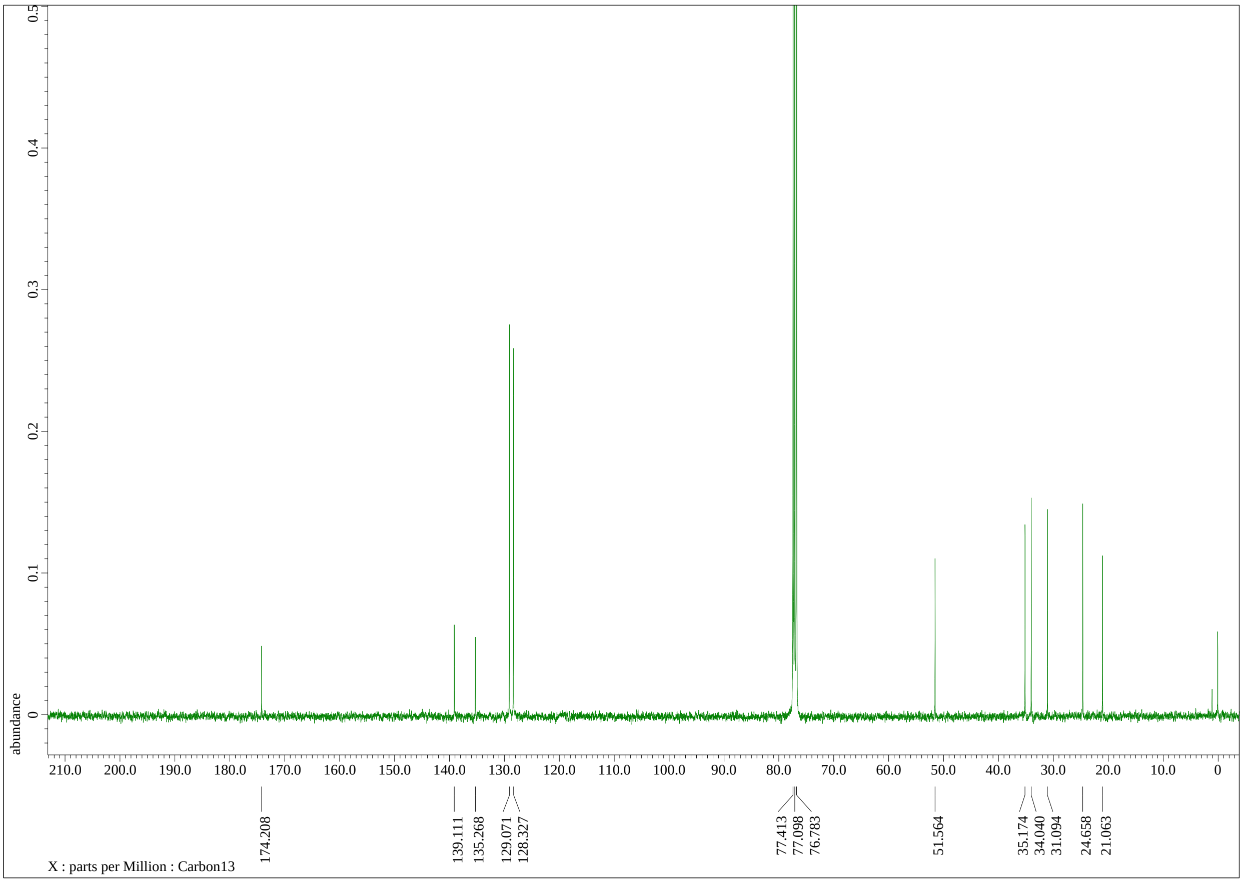
abundance
174.208
139.111
135.268
129.071
128.327
77.413
77.098
76.783
51.564
35.174
34.040
31.094
24.658
21.063
X : parts per Million : Carbon13

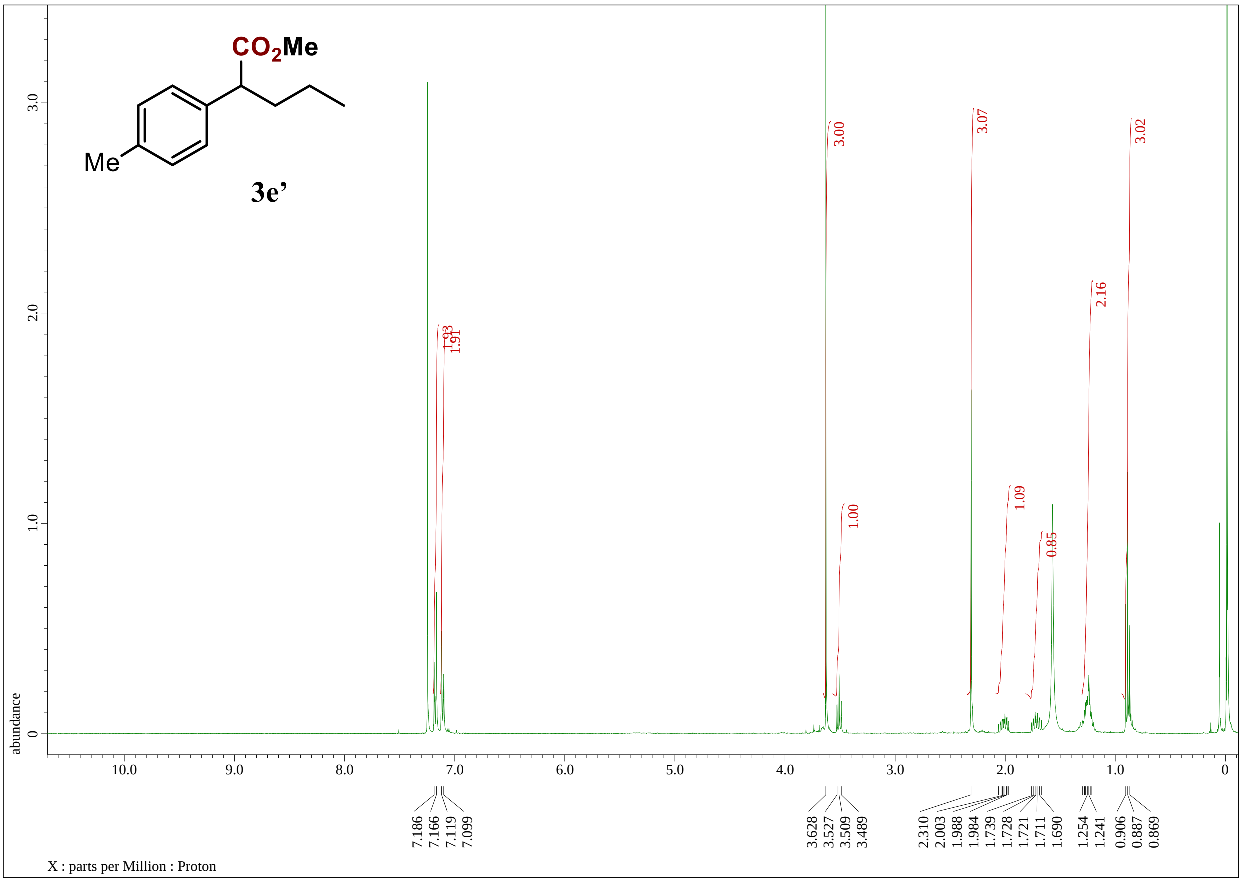

CO2Me
Me
3e’
3.00
3.07
3.02
1.93
1.91
1.00
1.09
0.85
2.16
abundance
X : parts per Million : Proton
7.186
7.166
7.119
7.099
3.628
3.527
3.509
3.489
2.310
2.003
1.988
1.984
1.739
1.728
1.721
1.711
1.690
1.254
1.241
0.906
0.887
0.869

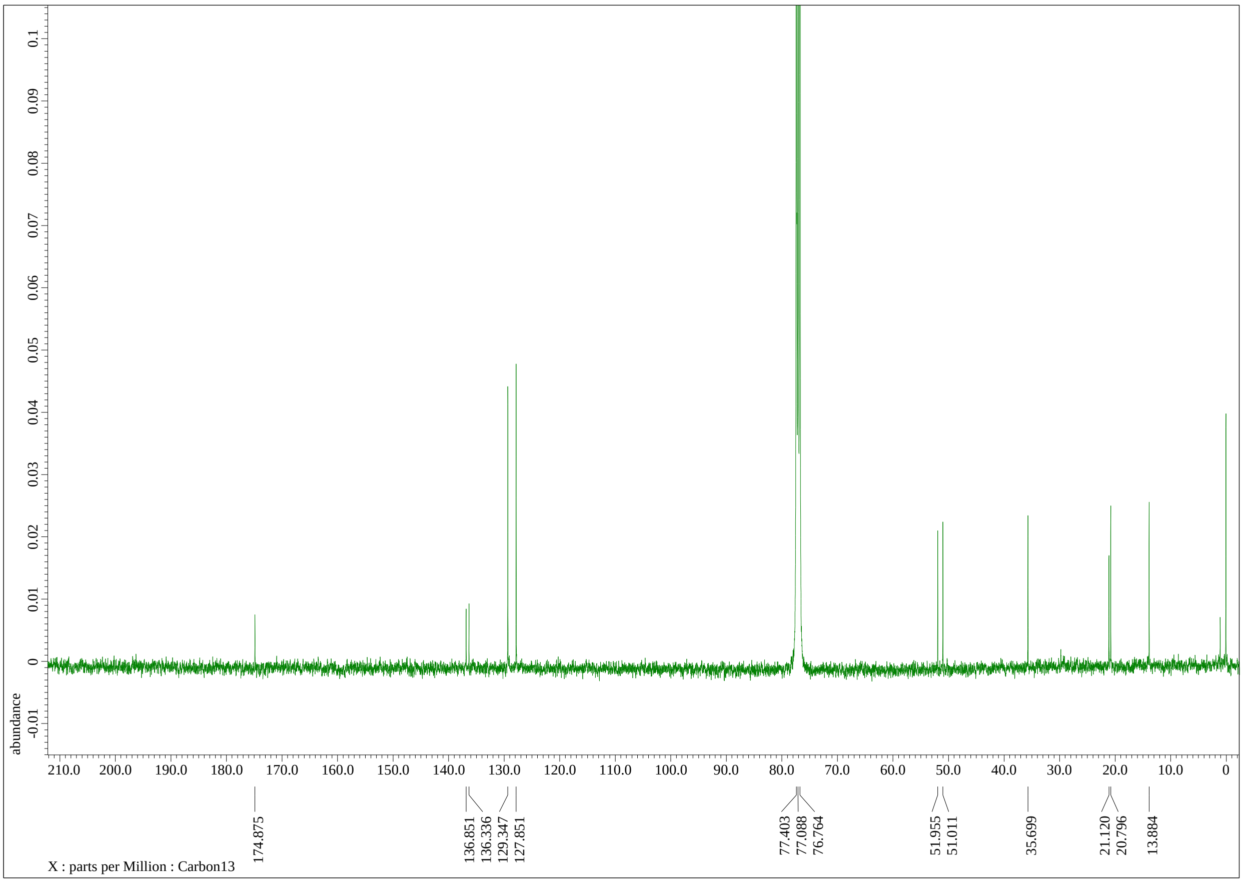

abundance
X : parts per Million : Carbon13
174.875
136.851
136.336
129.347
127.851
77.403
77.088
76.764
51.955
51.011
35.699
21.120
20.796
13.884

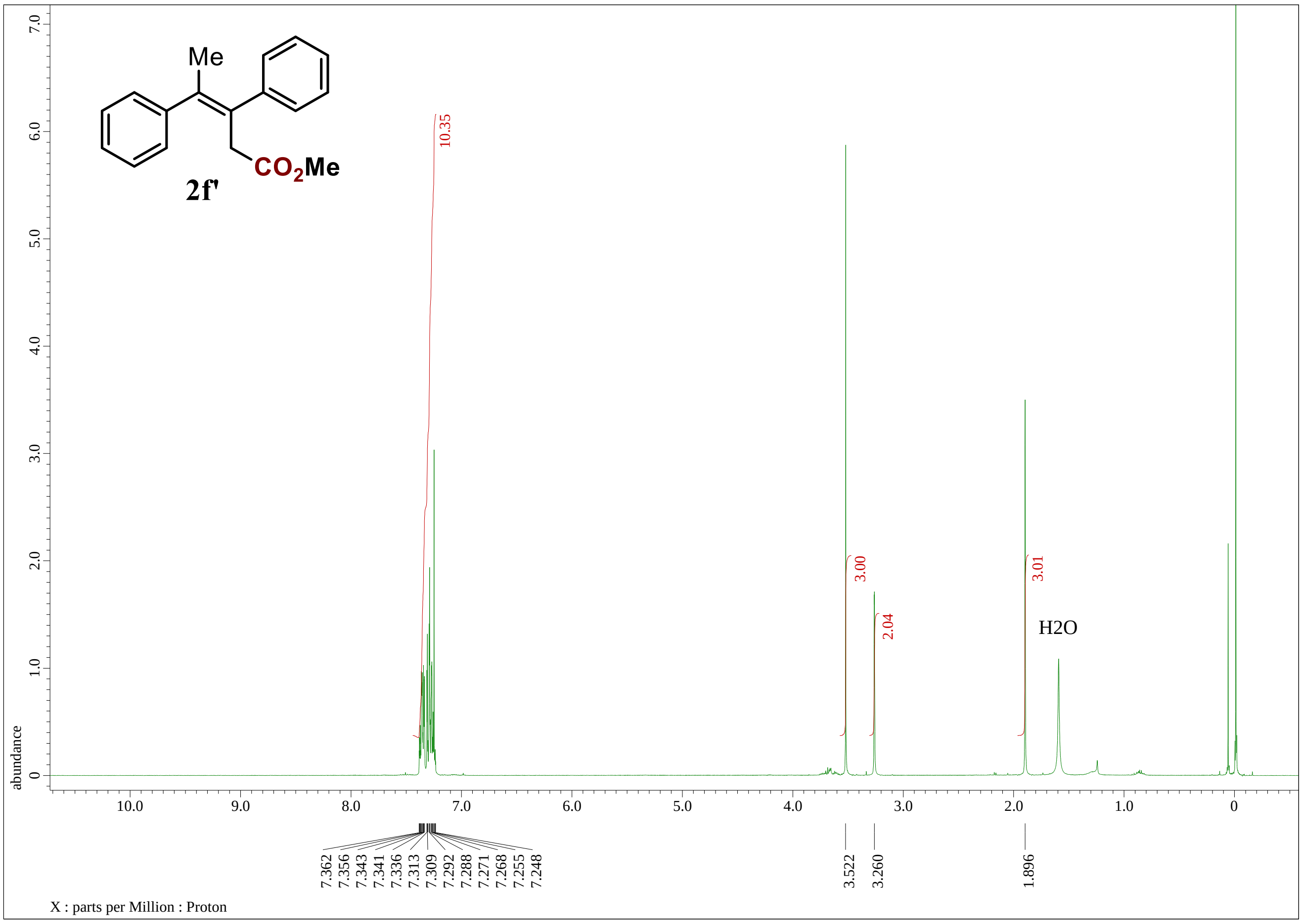
Me
CO2Me
2f'
10.35
3.00
2.04
3.01
H2O
abundance
7.362 7.356 7.343 7.341 7.336 7.313 7.309 7.292 7.288 7.271 7.268 7.255 7.248
3.522
3.260
1.896
X : parts per Million : Proton

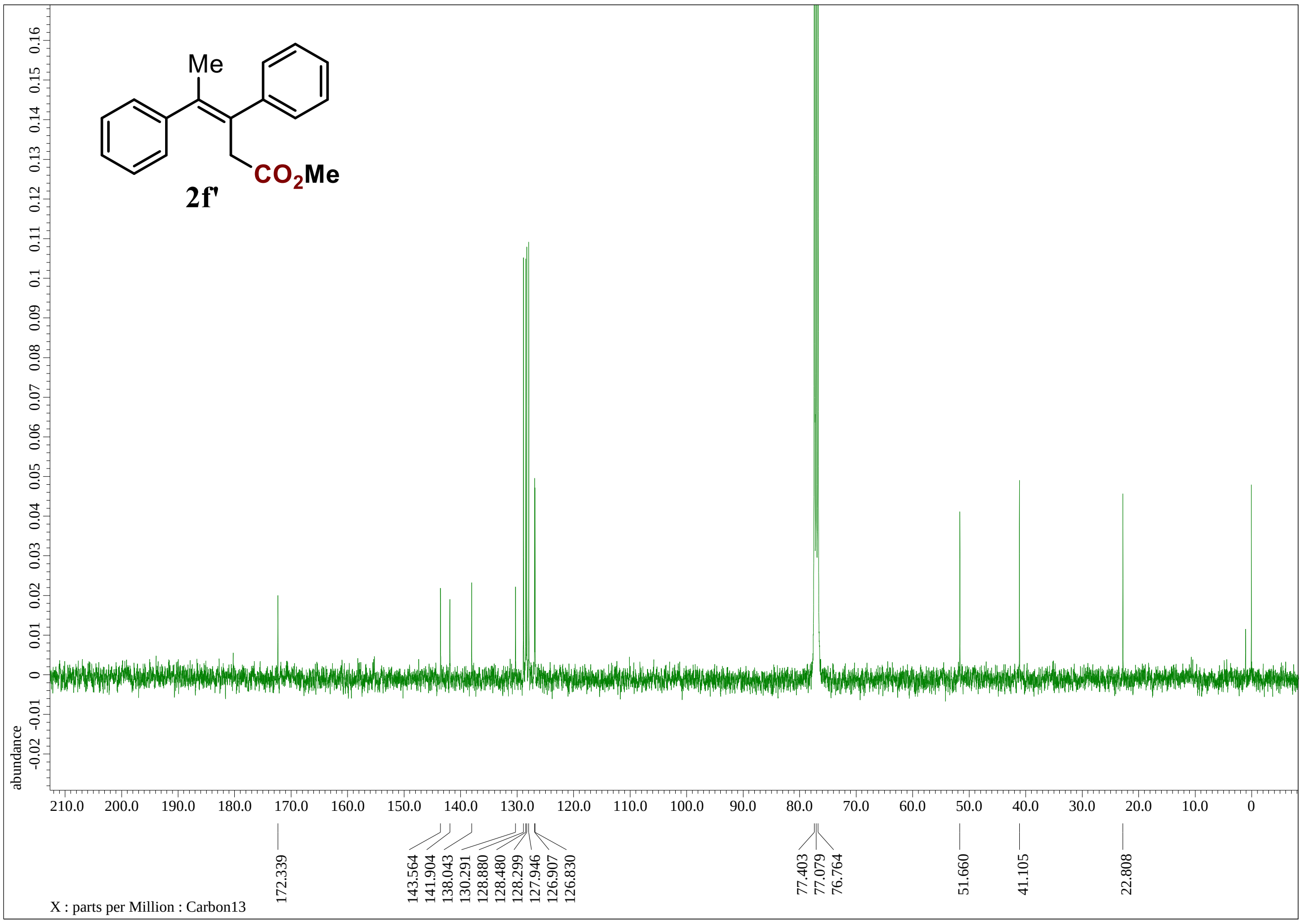
Me
CO2Me
2f'
abundance
172.339
143.564 141.904 138.043 130.291 128.880 128.480 128.299 127.946 126.907 126.830
77.403 77.079 76.764
51.660
41.105
22.808
X : parts per Million : Carbon13

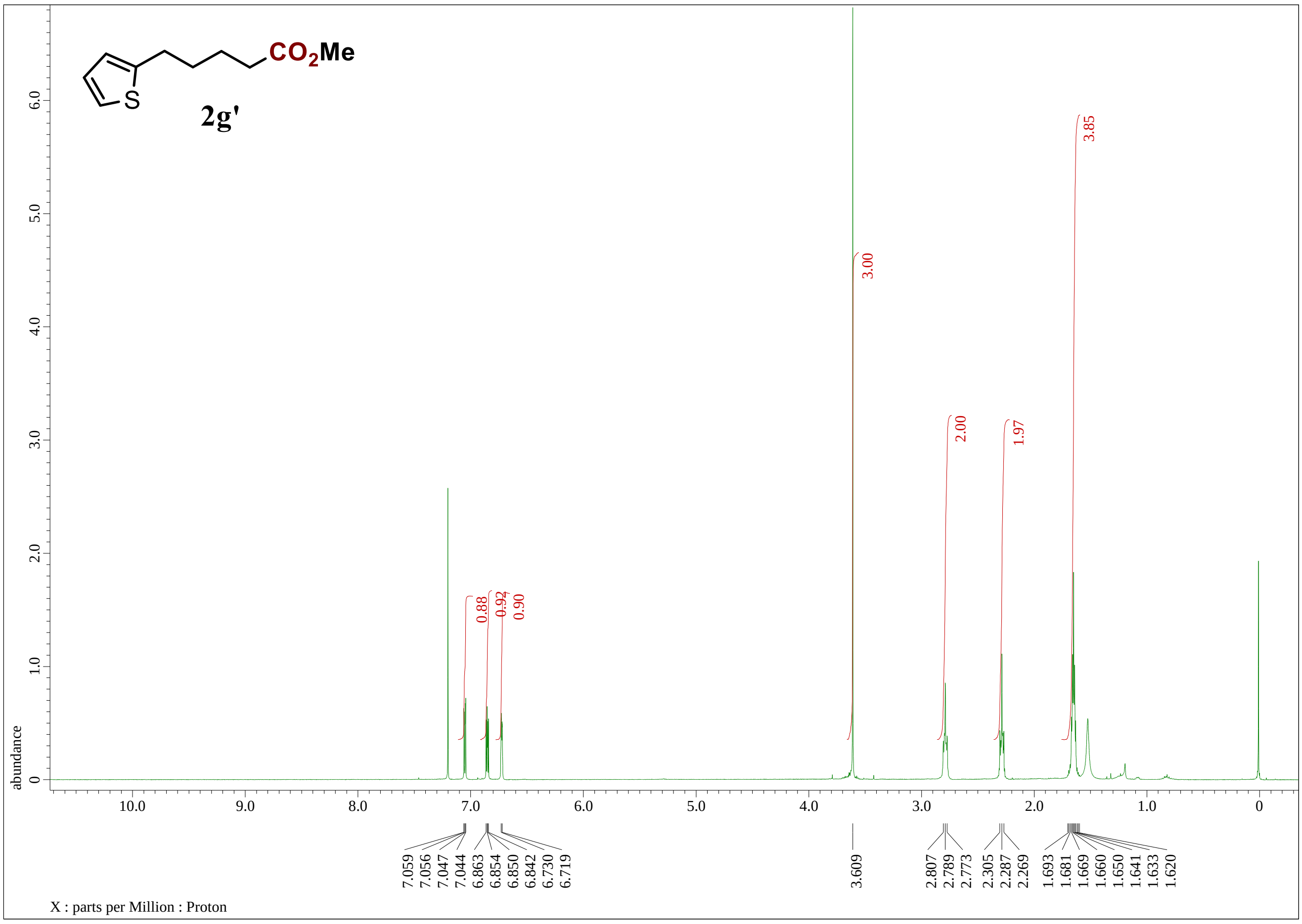

CO2Me
S
2g'
abundance
3.00
2.00
1.97
3.85
0.88
0.92
0.90
7.059
7.056
7.047
7.044
6.863
6.854
6.850
6.842
6.730
6.719
3.609
2.807
2.789
2.773
2.305
2.287
2.269
1.693
1.681
1.669
1.660
1.650
1.641
1.633
1.620
X : parts per Million : Proton

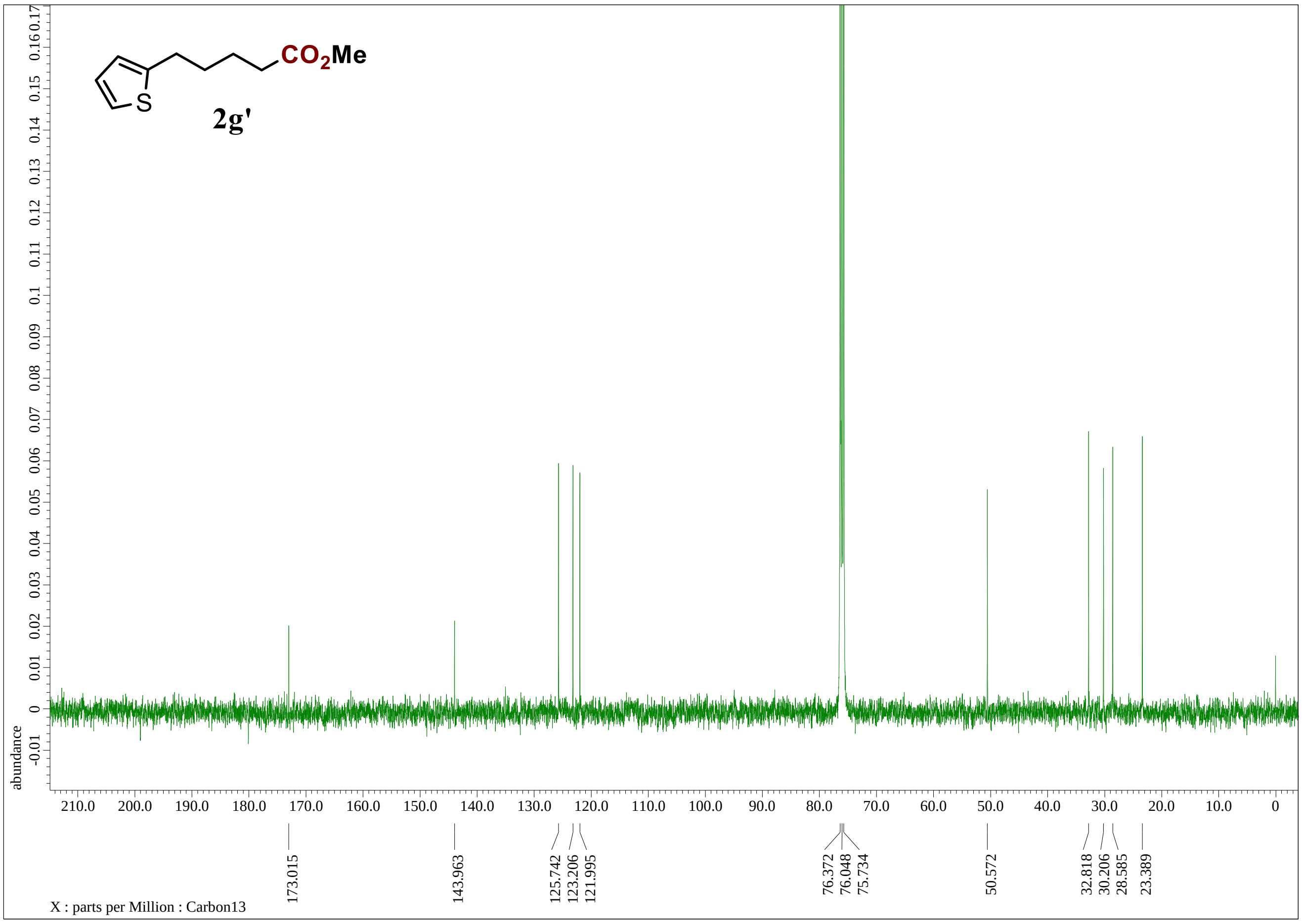

CO2Me
S
2g'
abundance
173.015
143.963
125.742
123.206
121.995
76.372
76.048
75.734
50.572
32.818
30.206
28.585
23.389
X : parts per Million : Carbon13

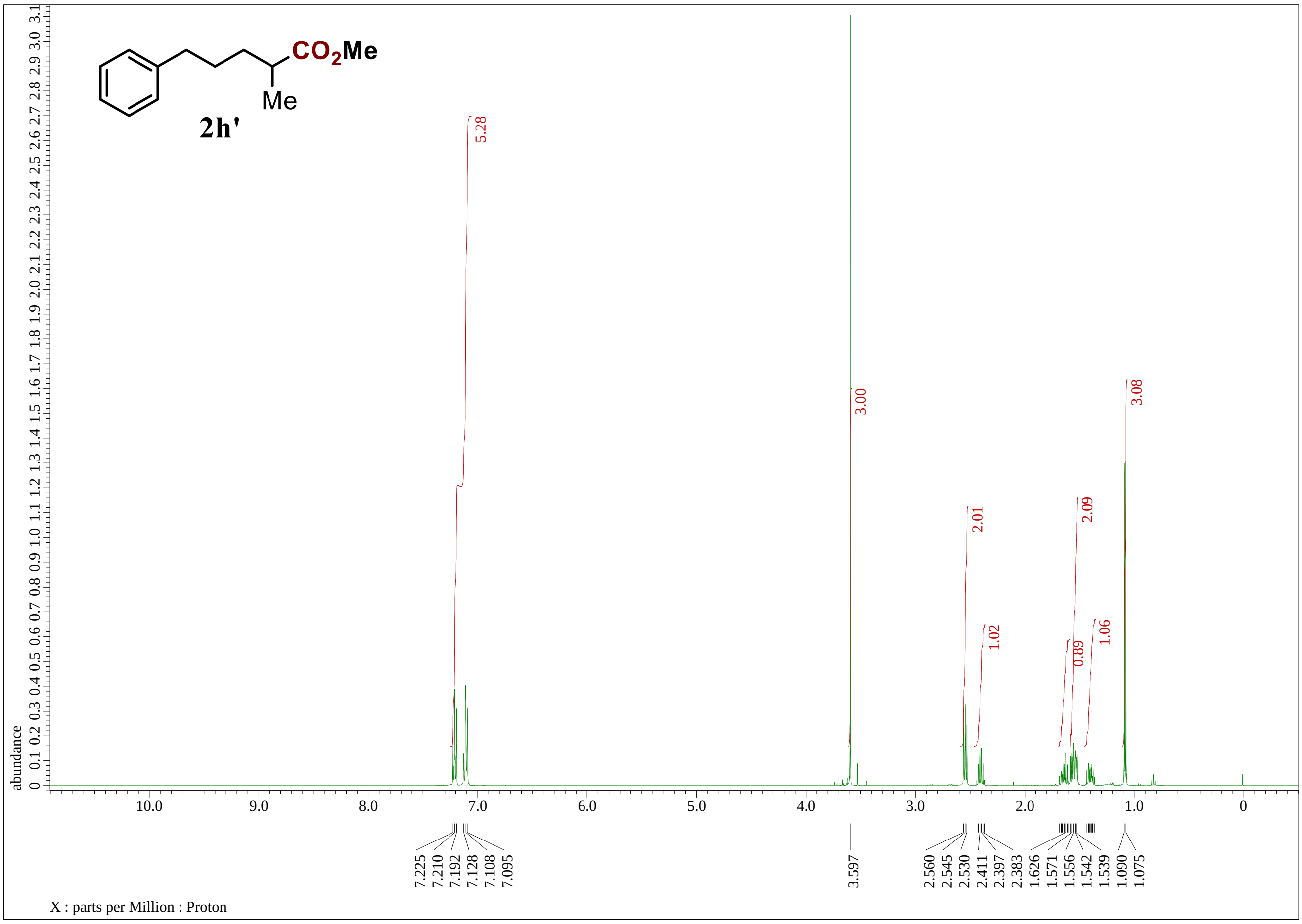
CO2Me
Me
2h'
abundance
5.28
3.00
2.01
1.02
0.89
2.09
1.06
3.08
7.225
7.210
7.192
7.128
7.108
7.095
3.597
2.560
2.545
2.530
2.411
2.397
2.383
1.626
1.571
1.556
1.542
1.539
1.090
1.075
X : parts per Million : Proton

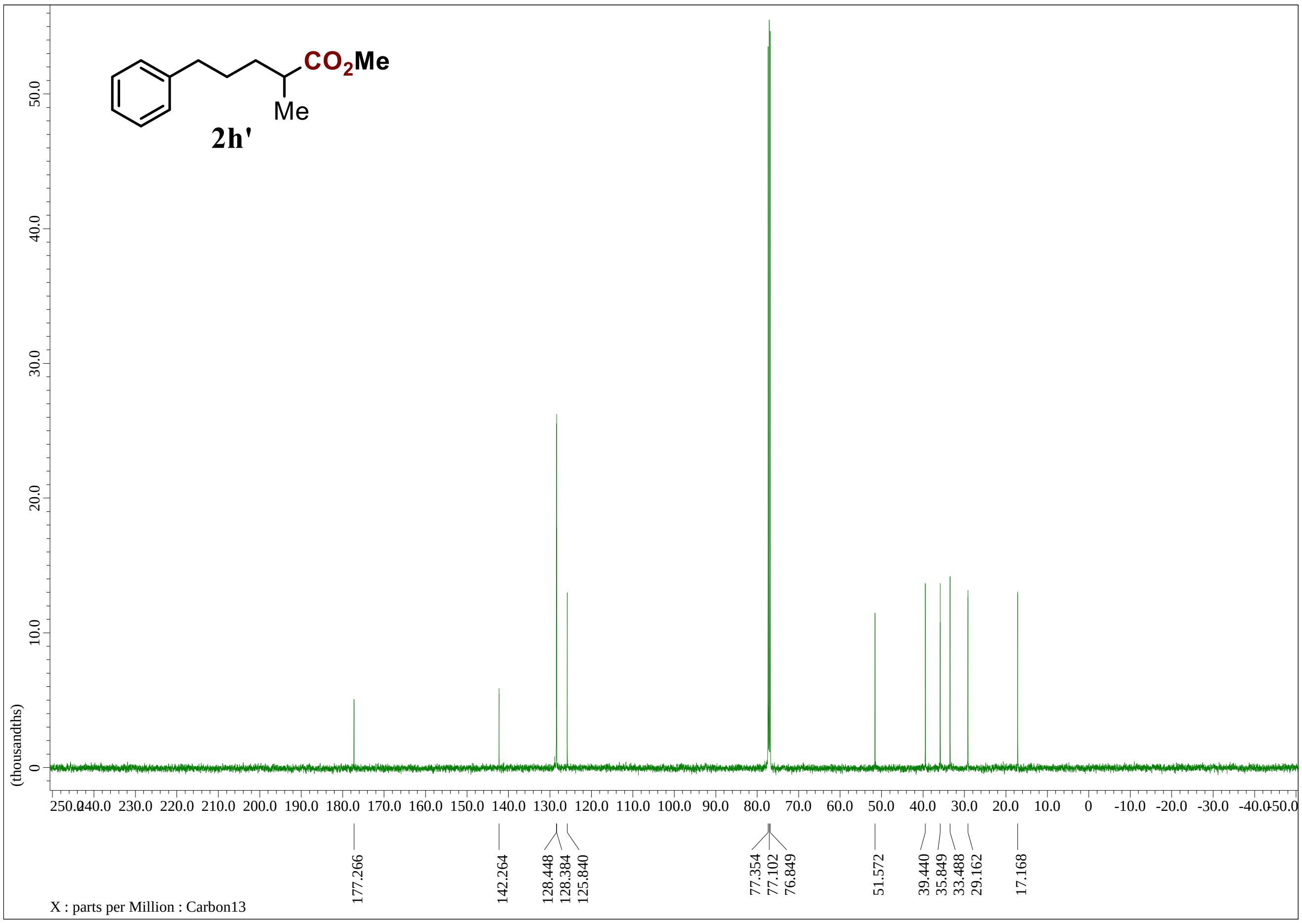
CO2Me
Me
2h'
(thousandths)
177.266
142.264
128.448
128.384
125.840
77.354
77.102
76.849
51.572
39.440
35.849
33.488
29.162
17.168
X : parts per Million : Carbon13

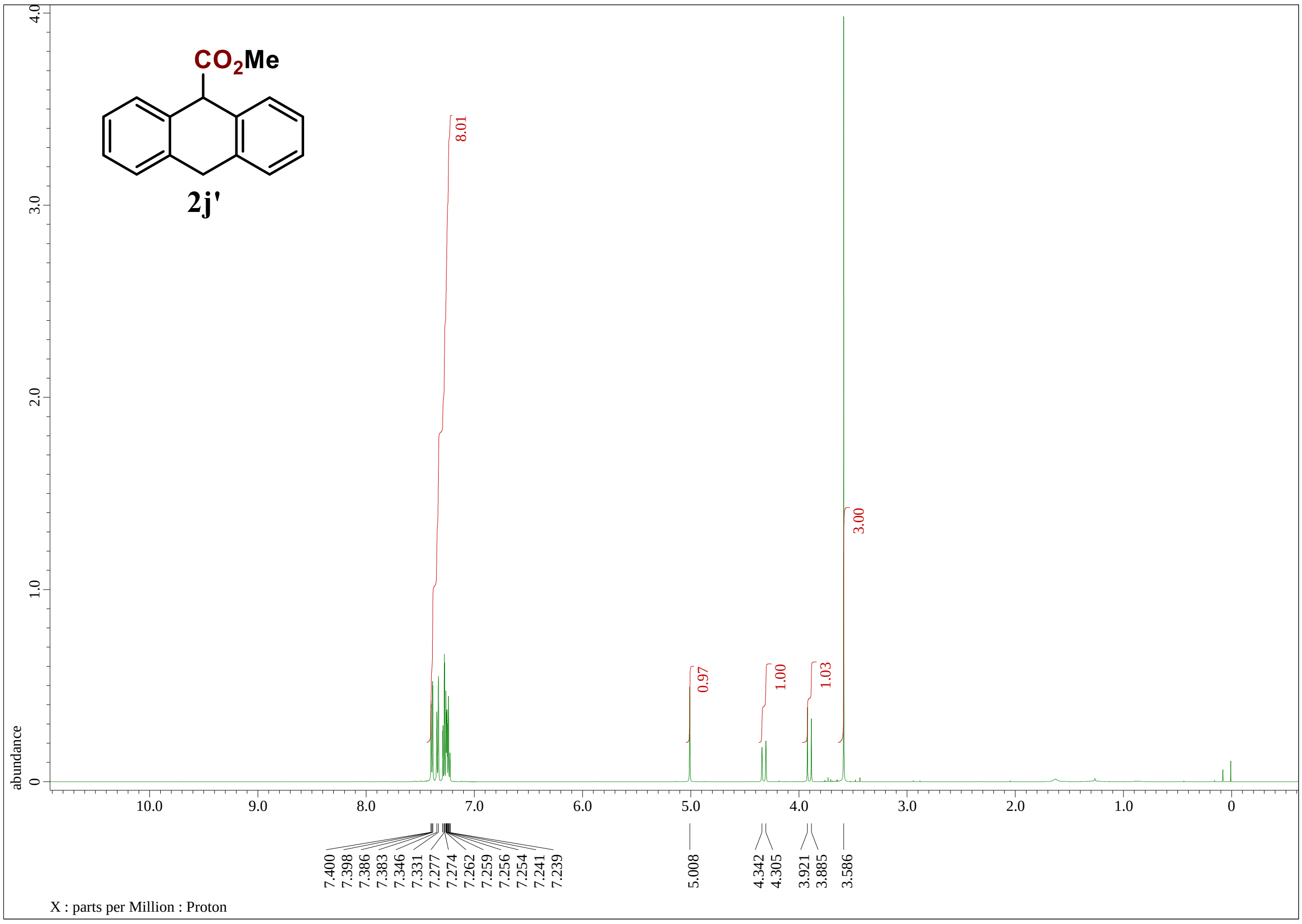
CO2Me
2j'
abundance
X : parts per Million : Proton
8.01
0.97
1.00
1.03
3.00
7.400
7.398
7.386
7.383
7.346
7.331
7.277
7.274
7.262
7.259
7.256
7.254
7.241
7.239
5.008
4.342
4.305
3.921
3.885
3.586

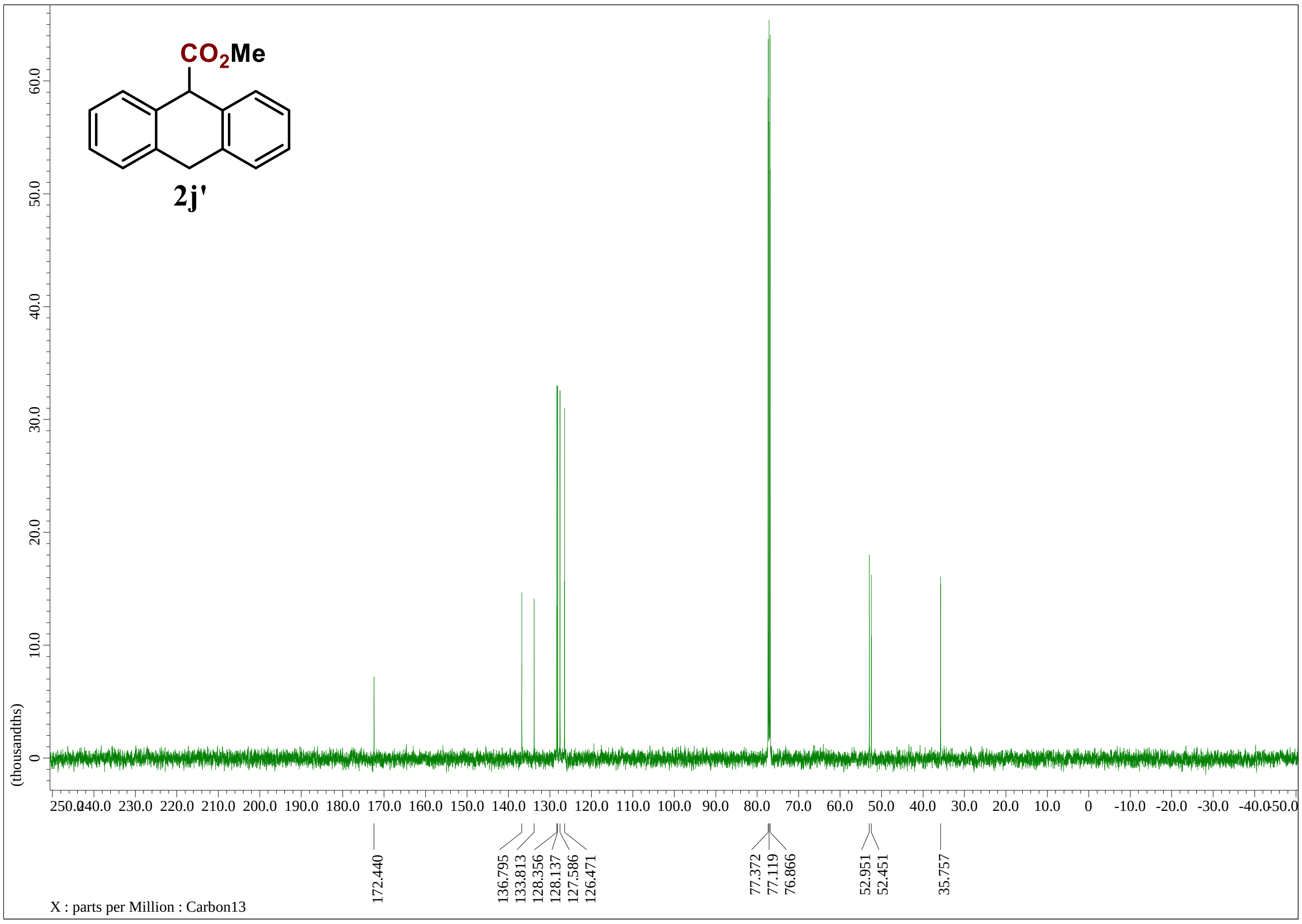
CO2Me
2j'
(thousandths)
X : parts per Million : Carbon13
172.440
136.795
133.813
128.356
128.137
127.586
126.471
77.372
77.119
76.866
52.951
52.451
35.757

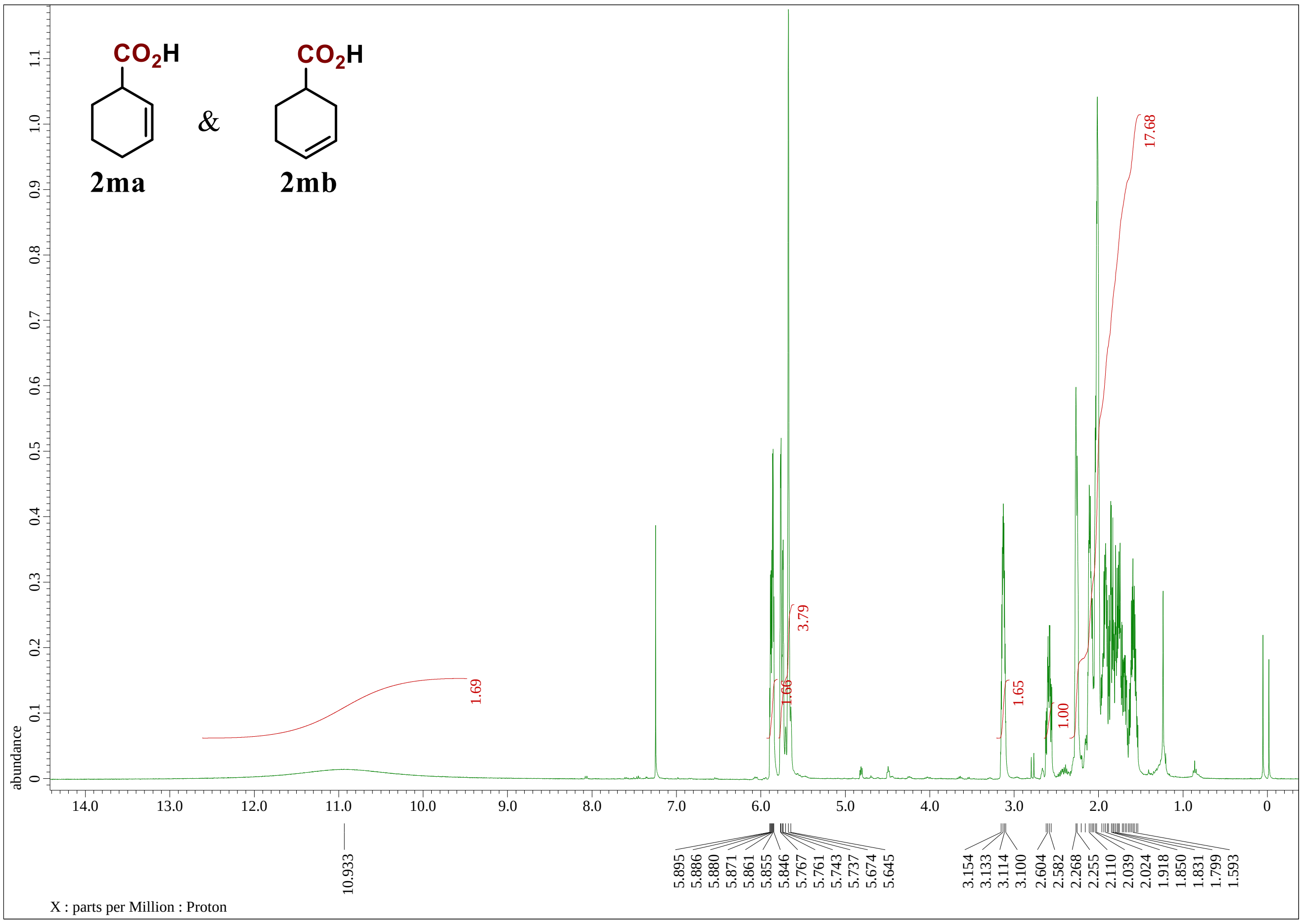

CO2H
&
CO2H
2ma
2mb
abundance
1.69
3.79
1.66
1.65
1.00
17.68
10.933
5.895 5.886 5.880 5.871 5.861 5.855 5.846 5.767 5.761 5.743 5.737 5.674 5.645
3.154 3.133 3.114 3.100 2.604 2.582 2.268 2.255 2.110 2.039 2.024 1.918 1.850 1.831 1.799 1.593
X : parts per Million : Proton

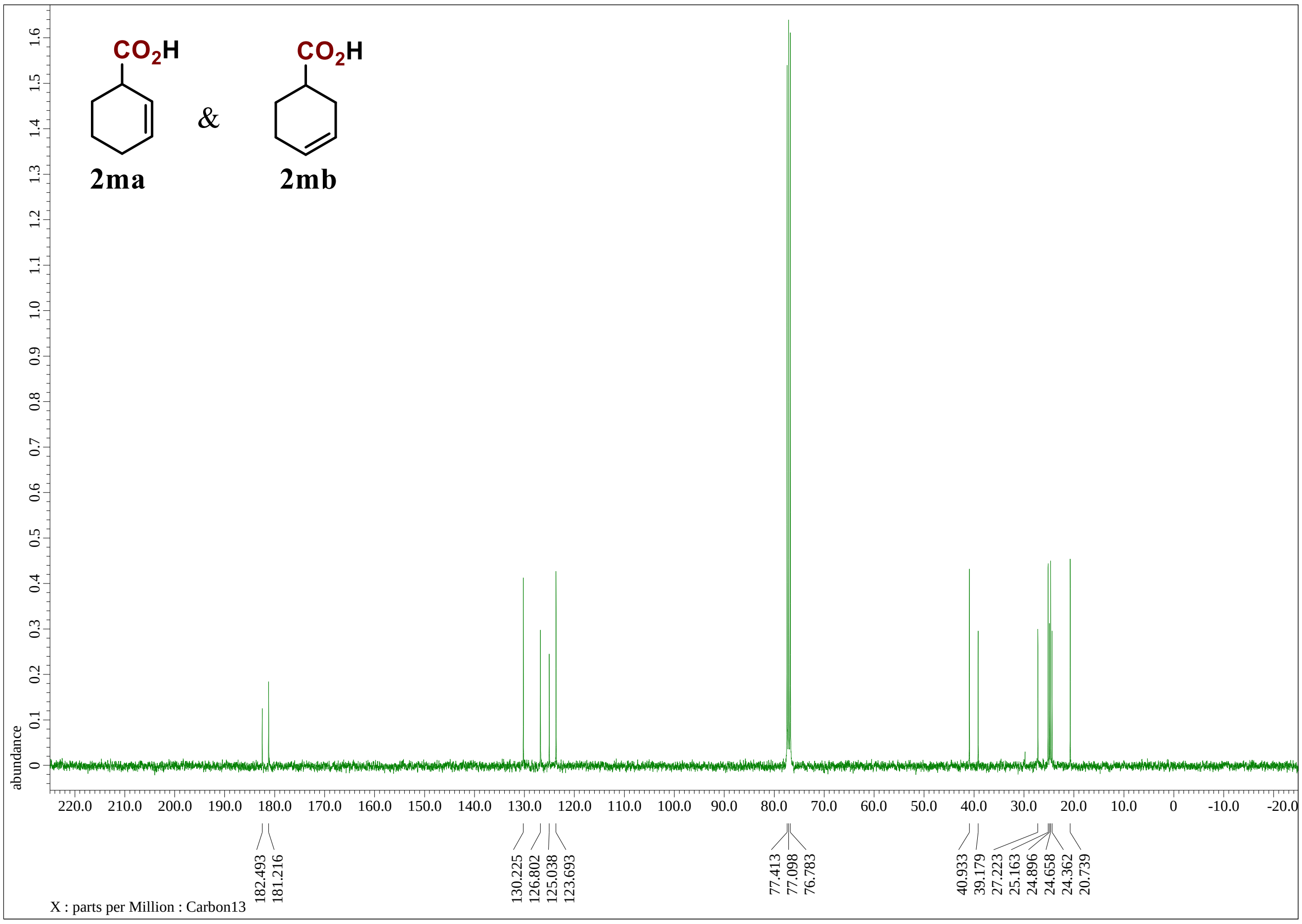

CO2H
&
CO2H
2ma
2mb
abundance
182.493 181.216
130.225 126.802 125.038 123.693
77.413 77.098 76.783
40.933 39.179 27.223 25.163 24.896 24.658 24.362 20.739
X : parts per Million : Carbon13

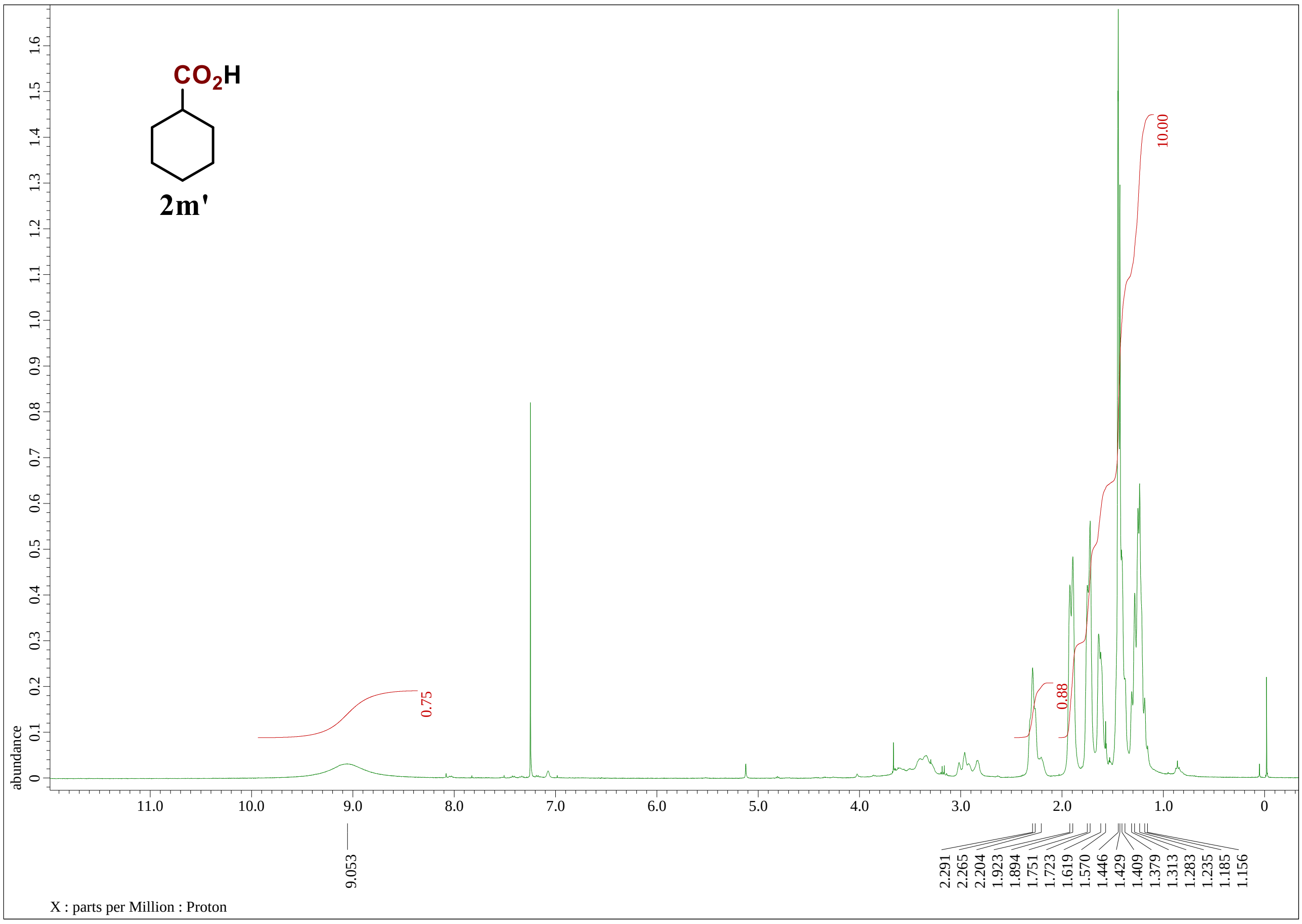
CO2H
2m'
abundance
0.75
0.88
10.00
9.053
2.291
2.265
2.204
1.923
1.894
1.751
1.723
1.619
1.570
1.446
1.429
1.409
1.379
1.313
1.283
1.235
1.185
1.156
X : parts per Million : Proton

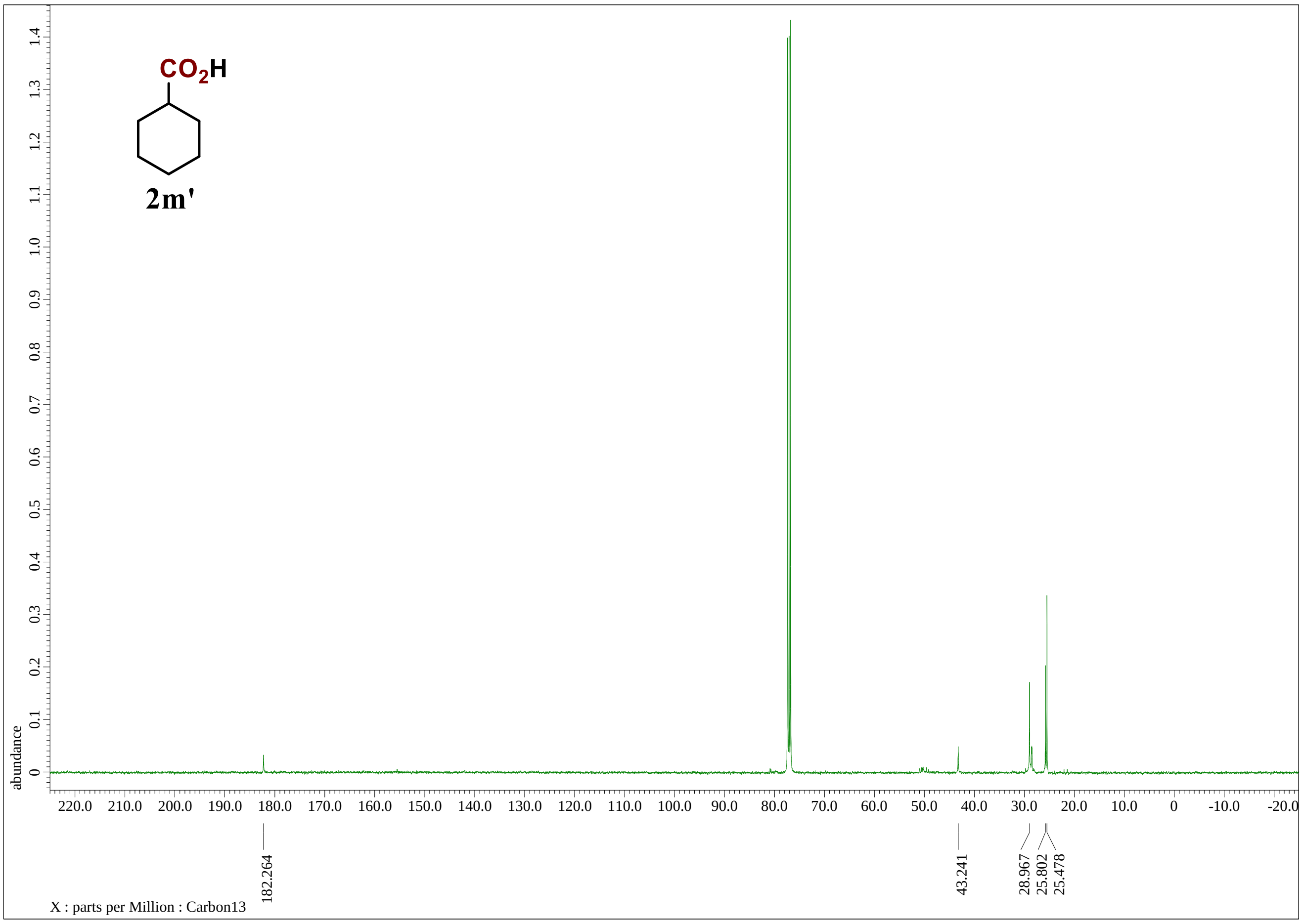
CO2H
2m'
abundance
182.264
43.241
28.967
25.802
25.478
X : parts per Million : Carbon13

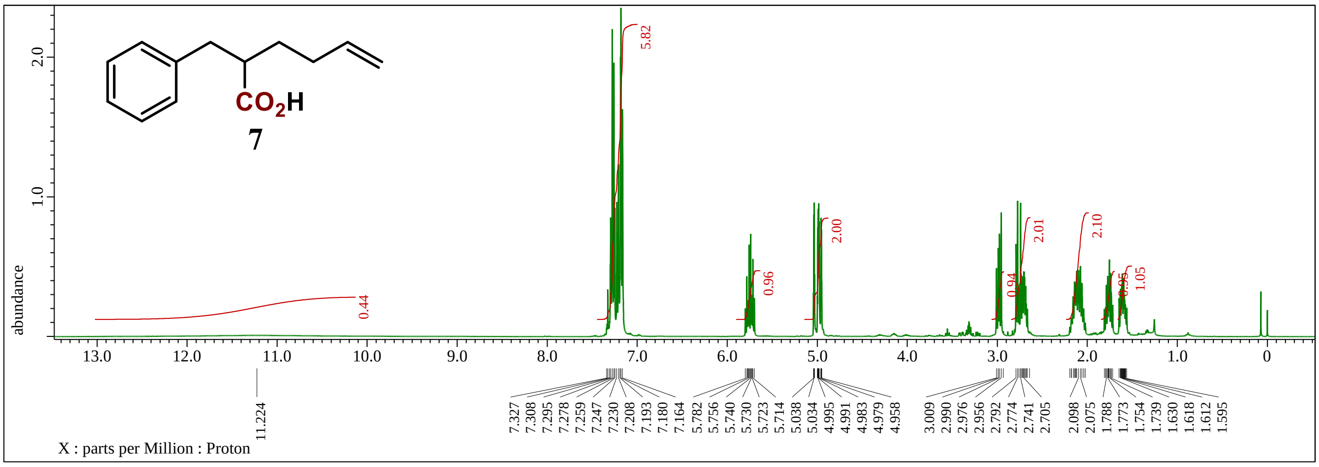
CO2H
7
abundance
5.82
0.96
2.00
0.94
2.01
2.10
0.95
1.05
0.44
11.224
7.327 7.308 7.295 7.278 7.259 7.247 7.230 7.208 7.193 7.180 7.164
5.782 5.756 5.740 5.730 5.723 5.714
5.038 5.034 4.995 4.991 4.983 4.979 4.958
3.009 2.990 2.976 2.956 2.792 2.774 2.741 2.705
2.098 2.075 1.788 1.773 1.754 1.739 1.630 1.618 1.612 1.595
X : parts per Million : Proton

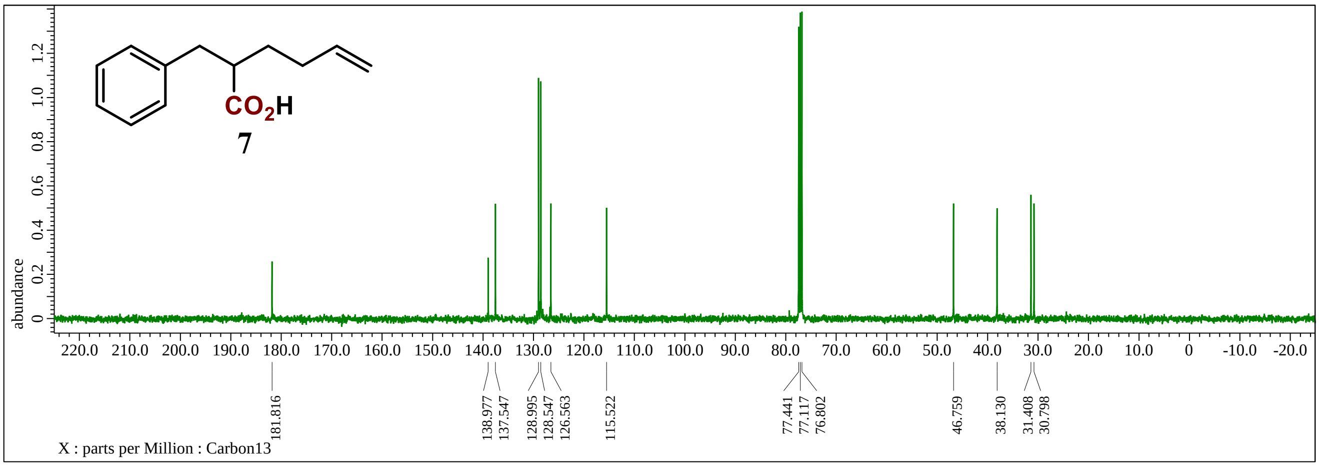
CO2H
7
abundance
181.816
138.977 137.547
128.995 128.547 126.563
115.522
77.441 77.117 76.802
46.759
38.130
31.408 30.798
X : parts per Million : Carbon13

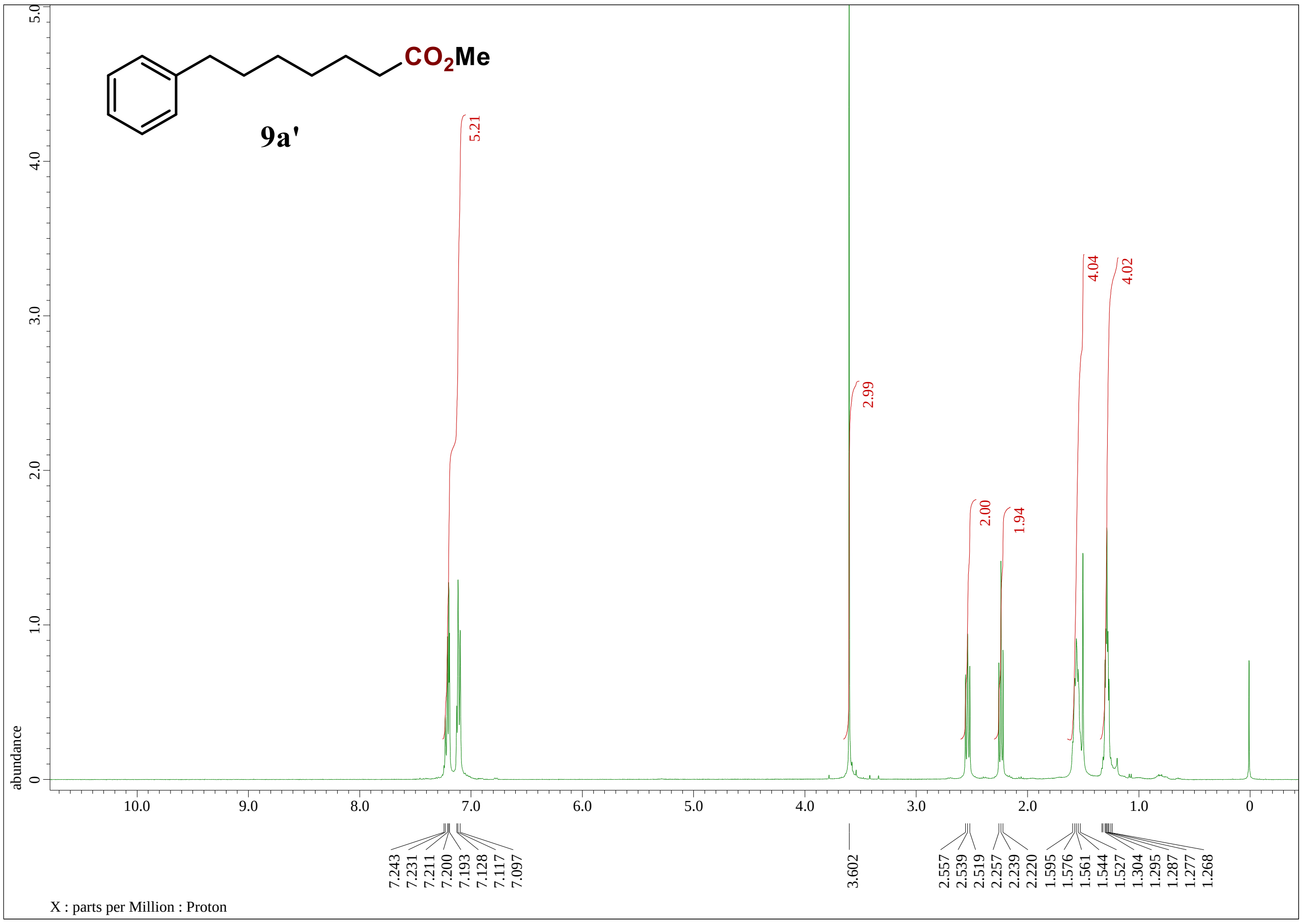
CO2Me
9a'
5.21
2.99
2.00
1.94
4.04
4.02
abundance
5.0
4.0
3.0
2.0
1.0
0
10.0
9.0
8.0
7.0
6.0
5.0
4.0
3.0
2.0
1.0
0
7.243
7.231
7.211
7.200
7.193
7.128
7.117
7.097
3.602
2.557
2.539
2.519
2.257
2.239
2.220
1.595
1.576
1.561
1.544
1.527
1.304
1.295
1.287
1.277
1.268
X : parts per Million : Proton

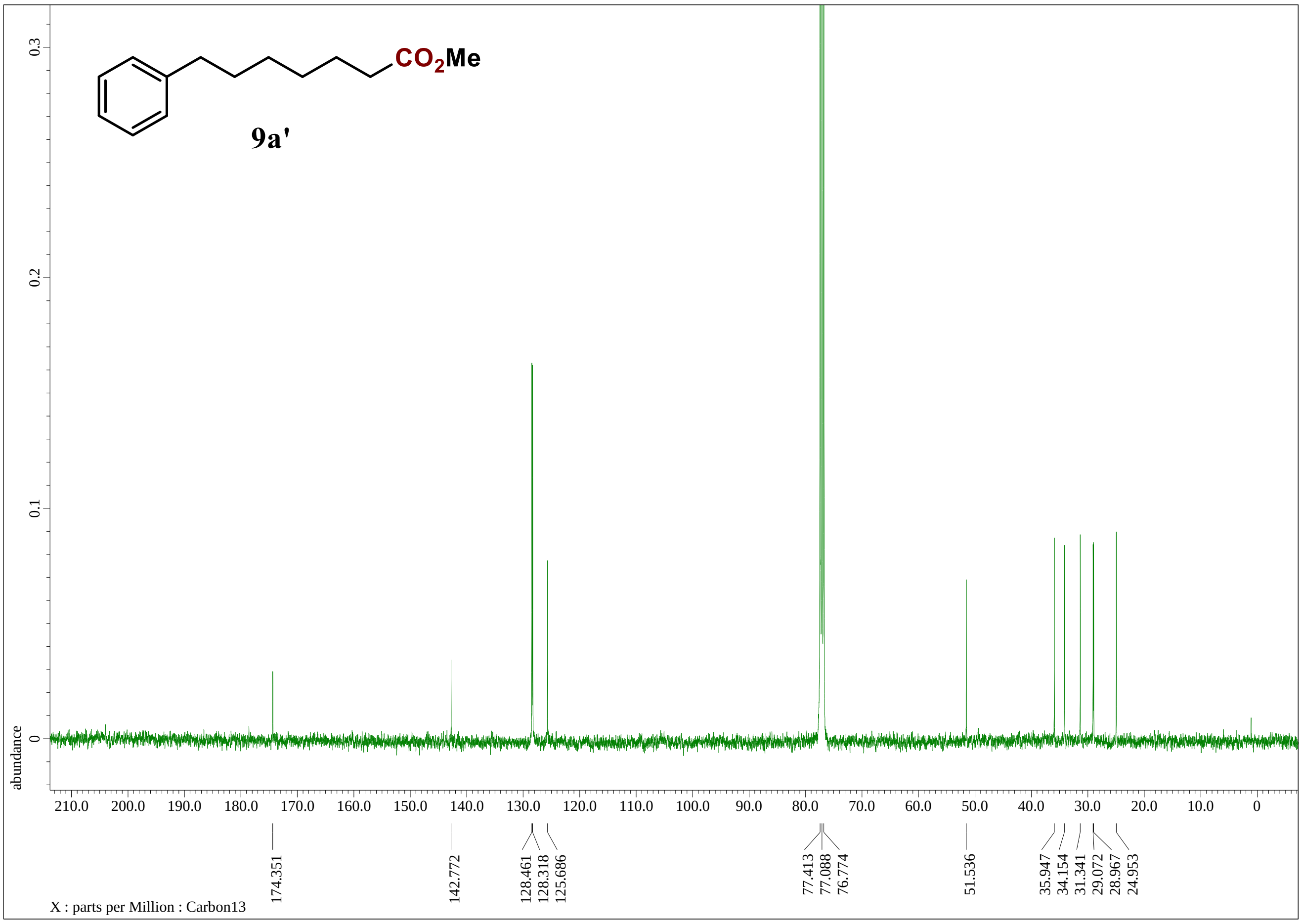
CO2Me
9a'
abundance
0.3
0.2
0.1
0
210.0
200.0
190.0
180.0
170.0
160.0
150.0
140.0
130.0
120.0
110.0
100.0
90.0
80.0
70.0
60.0
50.0
40.0
30.0
20.0
10.0
0
174.351
142.772
128.461
128.318
125.686
77.413
77.088
76.774
51.536
35.947
34.154
31.341
29.072
28.967
24.953
X : parts per Million : Carbon13

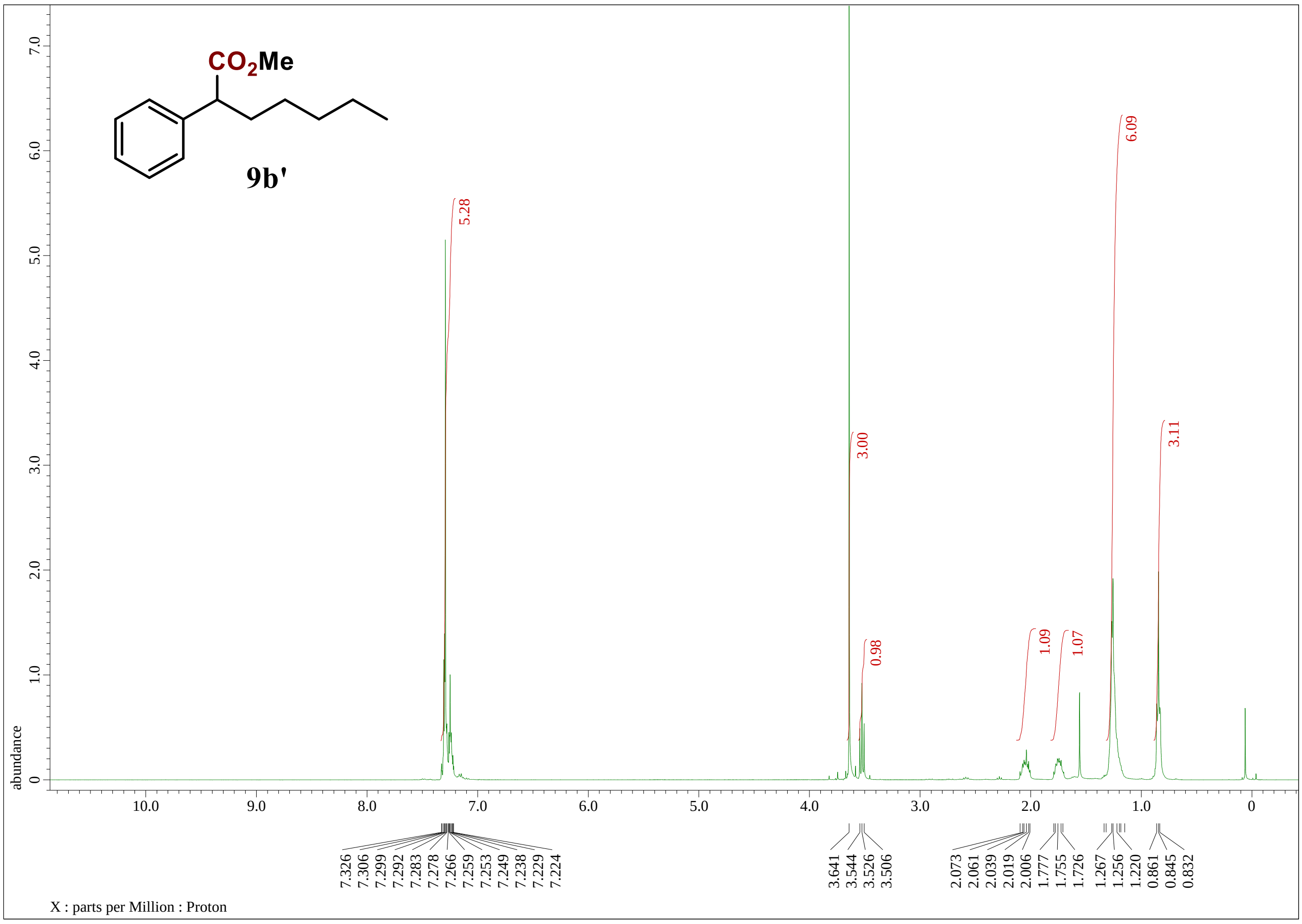
CO2Me
9b'
5.28
3.00
0.98
1.09
1.07
6.09
3.11
abundance
7.326 7.306 7.299 7.292 7.283 7.278 7.266 7.259 7.253 7.249 7.238 7.229 7.224
3.641 3.544 3.526 3.506
2.073 2.061 2.039 2.019 2.006 1.777 1.755 1.726
1.267 1.256 1.220 0.861 0.845 0.832
X : parts per Million : Proton

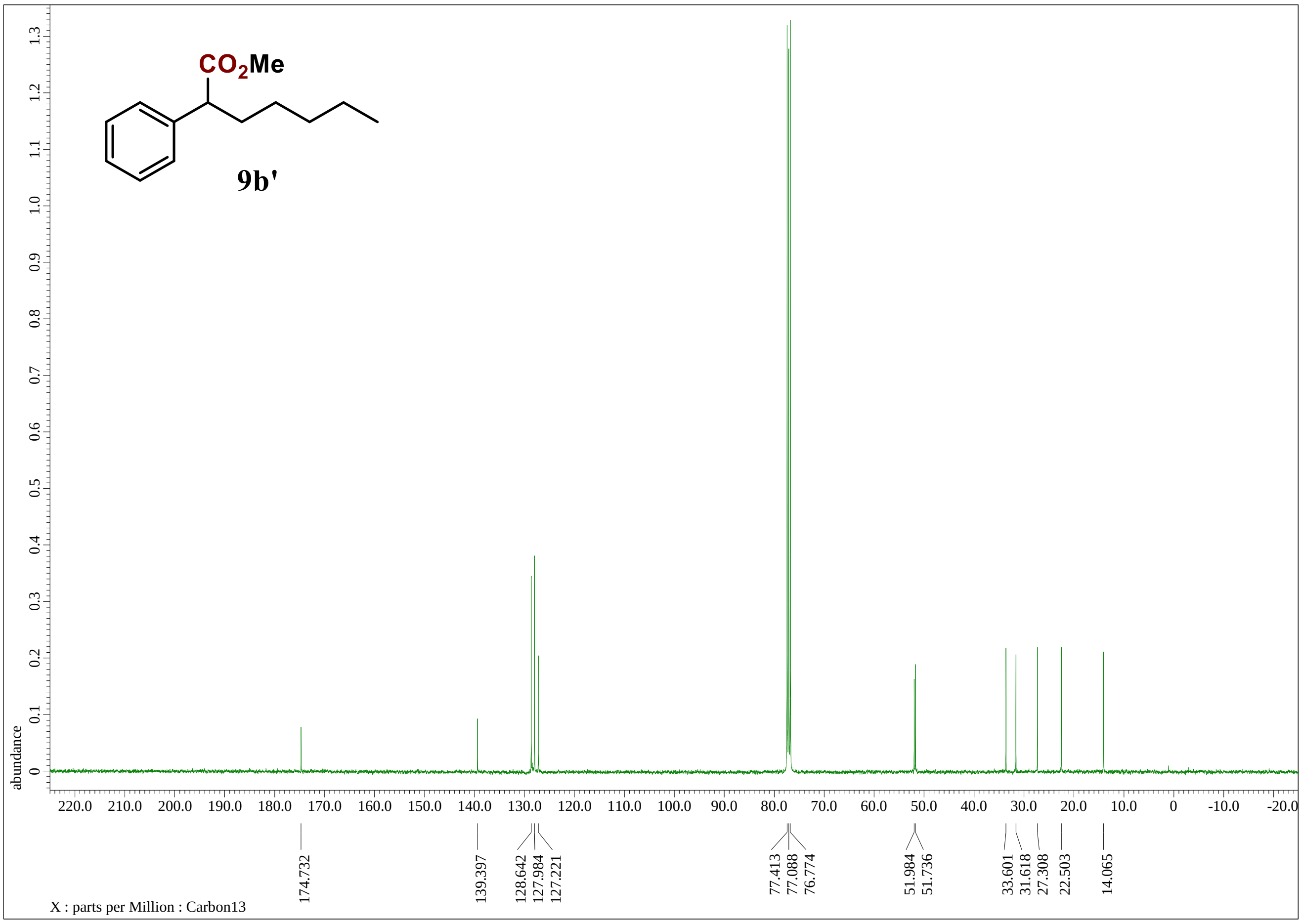
CO2Me
9b'
abundance
174.732
139.397
128.642 127.984 127.221
77.413 77.088 76.774
51.984 51.736
33.601 31.618 27.308 22.503
14.065
X : parts per Million : Carbon13
